# An Efficient Approach to Aromatic Aminomethylation Using Dichloromethane as Methylene Source

**DOI:** 10.3389/fchem.2019.00568

**Published:** 2019-08-13

**Authors:** Carmine Ostacolo, Veronica Di Sarno, Simona Musella, Tania Ciaglia, Vincenzo Vestuto, Giacomo Pepe, Fabrizio Merciai, Pietro Campiglia, Isabel M. Gomez Monterrey, Alessia Bertamino

**Affiliations:** ^1^Department of Pharmacy, University of Naples “Federico II”, Naples, Italy; ^2^Department of Pharmacy, University of Salerno, Fisciano, Italy; ^3^Fondazione EBRIS, Salerno, Italy

**Keywords:** aminomethylation, ultrasound assisted, dichloromethane, indole N-1 selectivity, aza-heterocyclic compounds

## Abstract

Ultrasound-promoted N-aminomethylation of indoles can be achieved in basic medium using sodium hydride and dichloromethane (DCM) as C1 donor source. This innovative amino methylation protocol results in good to excellent yields of multifunctional indole derivatives. The procedure is also applicable to other aza-heterocyclic compounds and, interestingly, affords direct access to aminomethyl-substituted aryl alcohols.

## Introduction

**CHART 1:**
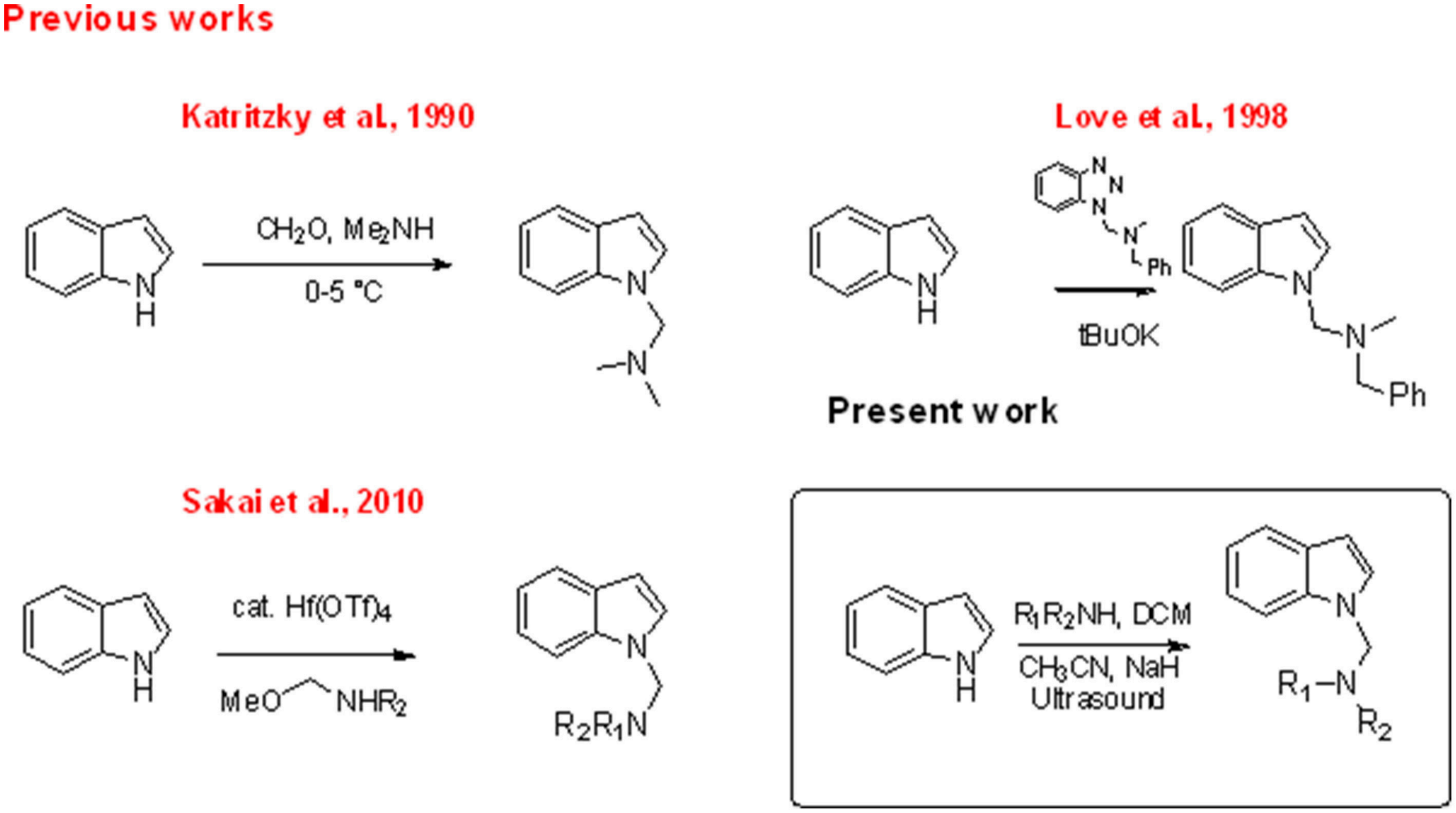
Aminomethylation of N-1 indole position.

The indole nucleus is present in a wide range of bioactive natural products and it is considered as privileged structure in the fields of pharmaceutical and material chemistry (Barden, 2010). Research of new synthetic metal- or organo-catalyzed methodologies for the rapid construction of functionalized indole has seen relevant progress in recent years (Patil and Yamamoto, 2008; Bandini and Eichholzer, 2009; Bartoli et al., 2010; Cacchi and Fabrizi, 2011; Dalpozzo, 2015; Leitch et al., 2017). Indole amino methylation, one of the most important methods for the direct formation of C–C and C–N bonds (Hwang and Uang, 2002; Ibrahem et al., 2004; Murai et al., 2012; Fujii et al., 2014; Nagae et al., 2015; Xu et al., 2015; Kim and Hong, 2017; Mastalir et al., 2017; Mondal et al., 2017), continues to be a challenge for chemists, especially indole aminomethylation at N-1 position. Mannich and Mannich-type Friedel–Crafts reactions, the later using imines, N,O acetals or N,N aminals in the presence of a Lewis acid, constitute the most commonly used chemical approaches for the construction of aminomethylated indoles (Chart 1; Swaminathan and Narasimhan, 1966; Katritzky et al., 1990; Matsumoto et al., 1993; Arend et al., 1998; Saaby et al., 2000; Speckamp and Moolenaar, 2000; Sakai et al., 2003, 2010, 2014; Jiang and Huang, 2005; Lindquist et al., 2006; Wang et al., 2006; Kang et al., 2007; Rowland et al., 2007; Alonso et al., 2008; Zou et al., 2015; Xie et al., 2018). However, both approaches are limited by the well-known regioselectivity toward the C-3 position when 1,3-non-substituted indoles are used (Sakai et al., 2014). The classic Mannich reaction at low temperatures (0–5°C) resulted in the high-yield synthesis of isogramines and derivatives (Katritzky et al., 1990). Under tBuOK-promoted basic conditions, Love and Nguyen (1998) and Love (2007) described the regioselective formation of the N-1 derivative using the reaction of unprotected indole with 1-(N,N'–dialkylaminomethyl) benzotriazoles as alkylating agents. Sakai et al. (2010) showed that, in the reaction of indoles with N,O acetals, the use of Hf(OTf)_4_ as Lewis acid regioselectively promoted N-aminomethylation. Mastalir et al. (2017) obtained an N1 derivative in basic medium by reaction of indole with a secondary amine using a manganese-based catalyst and methanol as C1 donor source. However, all these methods require highly controlled conditions or the presence of specific catalysts.

## Materials and Methods

### General Informations

Reagents, starting materials, and solvents were purchased from Sigma-Aldrich (Milan, Italy) and used as received. Reactions were carried out with magnetic stirring in 25 mL round-bottomed or in falcon tubes (10 mL). Ultrasonication was performed in a Bandelin Sonorex Digital 10P ultrasonic bath with a frequency of 60 Hz and power of 240 W. Microwave assisted closed vessel reactions were performed in a Biotage Initiator^+^ reactor, using 10 mL vials type and external temperature sensor. Analytical thin layer chromatography (TLC) was performed on pre-coated glass silica gel plates 60 (F254, 0.25 mm, VWR International). UHPLC analyses were performed on a Nexera UHPLC system (Shimadzu, Kyoto, Japan) consisting of a CBM-20A controller, two LC-30AD dual-plunger parallel-flow pumps, a DGU-20 AR5 degasser, an SPD-M20A photo diode array detector (equipped with a 2.5 μL detector flow cell volume), a CTO-20A column oven, a SIL-30AC autosampler. The chromatographic profile was obtained on a Kinetex™ C18 150 × 2.1 mm × 2.6 μm (100 Å) column (Phenomenex, Bologna, Italy). The optimal mobile phase consisted of 0.1% TFA/H_2_O v/v (A) and 0.1% TFA/ACN v/v (B). Analysis was performed in gradient elution as follows: 0–13.00 min, 5–65% B; 13–14.00 min, 65–95% B; 14–15.00 min, isocratic to 95% B; 15–15.01 min, 95–5% B; then 3 min for column re-equilibration. Flow rate was 0.5 mL min^−1^. Column oven temperature was set to 45°C. Injection volume was 2 μL of sample. The following PDA parameters were applied: sampling rate, 12.5 Hz; detector time constant, 0.160 s; cell temperature, 40°C. Data acquisition was set in the range 190–800 nm and chromatograms were monitored at 254 nm. For the quantification of main chromatographic peaks, indole was selected as external standard. Stock solution (1 mg mL^−1^) was prepared in methanol, the calibration curve was obtained in a concentration range of 250–10.0 μg mL^−1^ with six concentration levels and triplicate injection of each level were run. Peak areas of indole derivatives were plotted against corresponding concentrations (μg mL^−1^) and the linear regression was used to generate calibration curve (y = 0.00024x−1.39094) with R2 values was ≥ 0.9999. Purifications were conducted on the Biotage Isolera One flash purification system, using pre-packed KP-sil columns (Biotage, Uppsala, Sweden). 1D and 2D NMR spectra were recorded with Bruker Avance (400 MHz) spectrometer, at room temperature. Spectra were referenced to residual chloroform (7.24 ppm, 1H; 77.23 ppm, 13C) or methanol (3.31 ppm, 1H; 49.15 ppm, 13C). Chemical shifts are reported in δ values (ppm) relative to internal Me_4_Si, and J values are reported in hertz (Hz). The following abbreviations are used to describe peaks: s (singlet), d (doublet), dd (double doublet), t (triplet), bs (broad singlet), and m (multiplet). HR-MS experiments were performed by an LTQ-Orbitrap-XL-ETD mass spectrometer (Thermo Scientific, Bremen, Germany), using electrospray ionization. Elemental analysis was performed by the FlashSmart Elemental Analyzer (Thermo Fisher Scientific, Waltham, MA USA).

### Method Optimization

Indole (1 mmol), base (2 mmol), piperidine (1.5 mmol) were mixed in different solvents (5 mL) under the conditions reported in Table 1. After the time indicated in Table 1, the reaction was quenched with 5 mL of a 10% citric acid solution and the organic solvents were evaporated in vacuo. The crude was dissolved in DCM (20 mL) and extracted with water (3 × 20 mL). Compounds **2**, **3**, and **4** were obtained after flash chromatography, using 1/4 ethyl acetate/n-hexane as eluent mixture.

**Table 1:** Reaction of indole with piperidine using different approaches^a^.

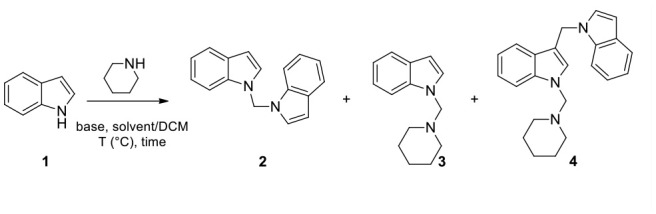					
**Entry**	**Time (min)**	**Reaction conditions**	**Yields (%)^**c**^**		
			**2**	**3**	**4**
1	20	US^b^ (50°C)	21	24	–
2	40	US (50°C)	30	39	–
3	80	US (50°C)	39	44	–
4	120	US (50°C)	40	51	–
5	180	US (50°C)	40	37	12
6	120	*T* = 25°C	31	16	2
7	120	*T* = 80°C	52	34	6
8	10	μW (100°C)	26	39	10

a *Reaction conditions: 1.0 mmol of indole, 1.5 mmol of piperidine, 2.0 mmol of base, 4 mL of DCM, 1 mL of DMF*.

b *Ultrasound irradiation*.

c *Yields were calculated with standardized HPLC method*.

### Application of the Optimized Procedure

Substrates (1 mmol) were dissolved in acetonitrile (5 mL) in a falcon tubes (10 mL) and sodium hydride (2 mmol), amines (1.5 mmol), and dichloromethane (3 mmol) were added. The mixture was introduced in an ultrasonic bath setting the temperature at 50°C and irradiating for 120 min. Then, the work up of the reaction and the purification of final compounds were performed as described above. The NMR spectra of synthesized compounds are depicted in Figures S1–S60.

#### Di(1H-Indol-1-yl)Methane (2)

R*f* = 0.70 (ethyl acetate/n-hexane 1/4). (Yield = 81.2 mg, 33%). ^1^H NMR (CDCl_3_, 400 MHz): δ: 6.37 (s, 2H, C*H*_2_); 6.57 (s 2H, aryl); 7.15–7.19 (m, 4H, aryl); 7.27 (t, 2H, aryl, *J* = 6.9 Hz); 7.49 (d, 2H, aryl, *J* = 8.2 Hz); 7.65 (d, 2H, aryl, *J* = 7.9 Hz). ^13^C NMR (CDCl_3_, 100 MHz) δ: 56.4; 103.4; 109.2; 120.3; 121.3; 122.5; 127.0; 129.1; 135.8. Elemental analysis calcd (%) for C_17_H_14_N_2_: C 82.90, H 5.73, N 11.37; found: C 83.06, H 5.70, N 11.31.

#### 1-(Piperidin-1-Ylmethyl)-1H-Indole (3)

R*f* = 0.40 (ethyl acetate/n-hexane 1/4). (Yield = 130.6 mg, 61%). ^1^H NMR (CDCl_3_, 400 MHz): δ: 1.29 (bs, 2H, C*H*_2_ piperidin); 1.48–1.51 (m, 4H, C*H*_2_ piperidin); 2.45 (t, 4H, C*H*_2_ piperidin, *J* = 4.7 Hz); 4.78 (s, 2H, C*H*_2_); 6.43 (d, 1H, aryl, *J* = 3.0 Hz); 7.02 (t, 1H, aryl, *J* = 7.1 Hz); 7.07 (d, 1H, aryl, *J* = 3.0 Hz); 7.13 (t, 1H, aryl, *J* = 7.1 Hz); 7.40 (d, 1H, aryl, *J* = 8.2 Hz); 7.55 (d, 1H, aryl, *J* = 7.8 Hz). ^13^C NMR (CDCl_3_, 100 MHz) δ: 23.9; 25.8; 51.8; 68.6; 101.3; 110.1; 119.5; 120.7; 121.6; 128.5; 128.8; 137.1. HR-MS *m/z*: calcd for C_14_H_19_N_2_, [(M+H)^+^]: 215.1543; found 215.1550. Elemental analysis calcd (%) for C_14_H_18_N_2_: C 78.46, H 8.47, N 13.07; found: C 78.54, H 8.40, N 13.11.

#### 3-((1H-Indol-1-yl)Methyl)-1-(Piperidin-1-Ylmethyl)-1H-Indole (4)

Obtained from indole and piperidine at 180 min. R*f* = 0.15 (ethyl acetate/n-hexane 1/4). (Yield = 41.2 mg, 12%). ^1^H NMR (CDCl_3_, 400 MHz): δ: 1.31–1.37 (m, 2H, C*H*_2_ piperidine); 1.51–1.57 (m, 4H, C*H*_2_ piperidine); 2.46 (bs, 4H, C*H*_2_ piperidine); 4.77 (s, 2H, C*H*_2_); 5.45 (s, 2H, C*H*_2_); 6.46 (d, 1H, aryl, *J* = 3.08 Hz); 7.02 (s, 1H, aryl); 7.04–7.13 (m, 3H, aryl); 7.16–7.21 (m, 2H, aryl); 7.42–7.46 (m, 3H, aryl): 7.62 (d, 1H, aryl, *J* = 7.8 Hz). ^13^C NMR (CDCl_3_, 100 MHz) δ: 23.9; 25.7; 41.9; 51.8; 68.5; 101.1; 109.6; 110.4; 118.8; 119.3; 119.8; 120.9; 121.3; 122.2; 127.0; 127.7; 127.8; 128.8; 137.7. HR-MS *m/z*: calcd for C_23_H_26_N_3_, [(M+H)^+^]: 344.2121; found 344.2130. Elemental analysis calcd (%) for C_23_H_25_N_3_: C 80.43, H 7.34, N 12.23; found: C 80.51, H 7.29, N 12.25.

#### 1,1′-(Piperidin-1-Ylmethylene)Bis(1H-Indole) (6)

R*f* = 0.55 (ethyl acetate/n-hexane 1/9). (Yield = 62.7 mg, 19%). ^1^H NMR (CDCl_3_, 400 MHz): δ: 1.52–1.54 (m, 2H, C*H*_2_ piperidin); 1.64 (t, 4H, C*H*_2_ piperidin, *J* = 5.0 Hz); 2.48 (t, 4H, C*H*_2_ piperidin, *J* = 5.0 Hz); 6.57 (d, 2H, aryl, *J* = 3.0 Hz); 6.85 (s, 1H, C*H*); 7.15 (t, 2H, aryl, *J* = 7.6 Hz); 7.23 (t, 2H, aryl, *J* = 7.6 Hz); 7.48 (d, 2H, aryl, *J* = 3.1 Hz); 7.53 (d, 2H, aryl, *J* = 8.2 Hz); 7.63 (d, 2H, aryl, *J* = 7.8 Hz). ^13^C NMR (CDCl_3_, 100 MHz) δ: 24.5; 25.7; 50.5; 82.4; 103.4; 109.9; 120.3; 121.1; 122.3; 124.9; 129.0; 135.9. HR-MS *m/z*: calcd for C_22_H_24_N_3_, [(M+H)^+^]: 330.1965; found 330.1977. Elemental analysis calcd (%) for C_22_H_23_N_3_: C 80.21, H 7.04, N 12.76; found: C 80.29, H 6.99, N 12.83.

#### 4-((1H-Indol-1-yl)Methyl)Morpholine (7)

Obtained from indole and morpholine. R*f* = 0.35 (ethyl acetate/n-hexane 1/4). (Yield = 147.0 mg, 68%). ^1^H NMR (CDCl_3_, 400 MHz): δ: 2.57 (t, 4H, C*H*_2_ morpholine, *J* = 4.3 Hz); 3.72 (t, 4H, C*H*_2_ morpholine, *J* = 4.3 Hz); 4.81 (s, 2H, C*H*_2_); 6.56 (d, 1H, aryl, *J* = 3.0 Hz); 7.14–7.18 (m, 2H, aryl); 7.27 (t, 1H, aryl, *J* = 8.4 Hz); 7.50 (d, 1H, aryl, *J* = 8.2 Hz); 7.67 (d, 1H, aryl, *J* = 7.7 Hz). ^13^C NMR (CDCl_3_, 100 MHz) δ: 50.9; 66.7; 68.3; 101.9; 110.1; 119.8; 120.9; 121.8; 128.4; 128.8; 135.2. HR-MS *m/z*: calcd for C_13_H_17_N_2_O, [(M+H)^+^]: 217.1335; found 217.1339. Elemental analysis calcd (%) for C_13_H_16_N_2_O: C 72.19, H 7.46, N 12.95, O 7.40; found: C 72.16, H 7.51, N 13.04, O 7.47.

#### N-((1H-Indol-1-yl)Methyl)-1-Phenylmethanamine (8)

Obtained from indole and benzylamine. R*f* = 0.25 (ethyl acetate/n-hexane 1/6). (Yield = 163.0 mg, 69%). ^1^H NMR (CDCl_3_, 400 MHz): δ: 3.59 (s, 2H, C*H*_2_ benzyl); 4.99 (s, 2H, C*H*_2_); 6.45 (d, 1H, aryl, *J* = 3.1 Hz); 7.04–7.08 (m, 2H, aryl); 7.12–7.31 (m, 7H, aryl); 7.59 (d, 1H, aryl, *J* = 7.8 Hz). ^13^C NMR (CDCl_3_, 100 MHz) δ: 50.0; 59.8; 101.5; 109.3; 119.7; 121.2; 121.7; 127.2; 127.9; 128.2; 128.5; 129.1; 135.8; 139.2. HR-MS *m/z*: calcd for C_17_H_19_N_2_, [(M+H)^+^]: 251.1543; found 251.1548. Elemental analysis calcd (%) for C_16_H_16_N_2_: C 81.32, H 6.82, N 11.85, found: C 81.41, H 6.90, N 11.87.

#### N-((1H-Indol-1-yl)Methyl)-2-Phenylethanamine (9)

Obtained from indole and phenethylamine. R*f* = 0.30 (ethyl acetate/n-hexane 1/4). (Yield = 177,6 mg, 71%). ^1^H NMR (CDCl_3_, 400 MHz): δ: 2.72 (t, 2H, C*H*_2_ ethyl, *J* = 6.8 Hz); 2.83 (t, 2H, C*H*_2_ ethyl, *J* = 6.8 Hz); 5.08 (s, 2H, C*H*_2_); 6.53 (d, 1H, aryl, *J* = 3.0 Hz); 7.11 (d, 2H, aryl, *J* = 7.2 Hz); 7.14–7.28 (m, 6H, aryl); 7.39 (d, 1H, aryl, *J* = 8.2 Hz); 7.68 (d, 1H, aryl, *J* = 8.0 Hz). ^13^C NMR (CDCl_3_, 100 MHz) δ: 36.2; 47.6; 60.7; 101.5; 109.2; 119.7; 121.1; 121.7; 126.3; 127.7; 128.5; 128.7; 129.0; 135.8; 139.4. HR-MS *m/z*: calcd for C_17_H_19_N_2_, [(M+H)^+^]: 251.1543; found 251.1548. Elemental analysis calcd (%) for C_17_H_18_N_2_: C 81.56, H 7.25, N 11.19; found: C 81.50, H 7.31, N 11.14.

#### N-((1H-Indol-1-yl)Methyl)-4-Methoxyaniline (10)

Obtained from indole and 4-methoxyaniline. R*f* = 0.50 (ethyl acetate/n-hexane 1/4). (Yield = 106.0 mg, 42%). ^1^H NMR (CDCl_3_, 400 MHz): δ: 3.75 (s, 3H, OC*H*_3_); 5.50 (s, 2H, C*H*_2_); 6.50 (d, 1H, aryl, *J* = 2.9 Hz); 6.66 (d, 2H, aryl, *J* = 8.8 Hz); 6.78 (d, 2H, aryl, *J* = 8.8 Hz); 7.17 (t, 1H, aryl, *J* = 7.3 Hz); 7.22 (d, 1H, aryl, *J* = 3.0 Hz); 7.27 (t, 1H, aryl, *J* = 7.6 Hz); 7.47 (d, 1H, aryl, *J* = 8.2 Hz); 7.66 (d, 1H, aryl, *J* = 7.8 Hz). ^13^C NMR (CDCl_3_, 100 MHz) δ: 55.7; 57.5; 101.8; 109.3; 115.0; 115.8; 119.8; 121.2; 121.8; 126.8; 129.2; 135.4; 139.7; 153.5. HR-MS *m/z*: calcd for C_16_H_17_N_2_O, [(M+H)^+^]: 253.1335; found 253.1341. Elemental analysis calcd (%) for C_16_H_16_N_2_O: C 76.16, H 6.39, N 11.10, O 6.34; found: C 76.18, H 6.42, N 11.03, O 6.40.

#### 5-Iodo-1-(Piperidin-1-Ylmethyl)-1H-Indole (11)

Obtained from 5-iodoindole and piperidine. R*f* = 0.45 (diethyl ether/n-hexane 1/1). (Yield = 200.6 mg, 59%). ^1^H NMR (CDCl_3_, 400 MHz): δ: 1.36–1.41 (m, 2H, C*H*_2_ piperidine); 1.56–1.61 (m, 4H, C*H*_2_ piperidine); 2.50 (t, 4H, C*H*_2_ piperidine, *J* = 4.6 Hz); 4.82 (s, 2H, C*H*_2_); 6.44 (d, 1H, aryl, *J* = 2.5 Hz); 7.13 (d, 1H, aryl, *J* = 3.1 Hz); 7.28 (d, 1H, aryl, *J* = 8.1 Hz); 7.46 (d, 1H, aryl, *J* = 8.6 Hz); 7.97 (s, 1H, aryl). ^13^C NMR (CDCl_3_, 100 MHz) δ: 23.9; 25.8; 51.8; 68.8; 83.0; 100.6; 112.2; 129.5; 129.9; 131.1; 136.5. HR-MS *m/z*: calcd for C_14_H_18_IN_2_, [(M+H)^+^]: 341.0509; found 341.0514. Elemental analysis calcd (%) for C_14_H_17_IN_2_: C 49.43, H 5.04, I 37.30, N 8.23, found: C 49.47, H 5.08, I 37.20, N 8.28.

#### 5-Methyl-1-(Piperidin-1-Ylmethyl)-1H-Indole (12)

Obtained from 5-methylindole and piperidine. R*f* = 0.55 (ethyl acetate/n-hexane 1/4). (Yield = 150.6 mg, 66%). ^1^H NMR (CDCl_3_, 400 MHz): δ: 1.37–1.42 (m, 2H, C*H*_2_ piperidine); 1.58–1.63 (m, 4H, C*H*_2_ piperidine); 2.50 (s, 3H, C*H*_3_); 2.55 (t, 4H, C*H*_2_ piperidine, *J* = 5.0 Hz); 4.86 (s, 2H, C*H*_2_); 6.47 (d, 1H, aryl, *J* = 3.0 Hz); 7.08 (d, 1H, aryl, *J* = 8.3 Hz); 7.15 (d, 1H, aryl, *J* = 3.0 Hz); 7.40 (d, 1H, aryl, *J* = 8.4 Hz); 7.45 (s, 1H, aryl). ^13^C NMR (CDCl_3_, 100 MHz) δ: 21.4; 23.9; 25.9; 51.8; 68.7; 100.7; 109.8; 120.4; 123.2; 128.7; 128.9; 135.6. HR-MS *m/z*: calcd for C_15_H_21_N_2_, [(M+H)^+^]: 229.1699; found 229.1705. Elemental analysis calcd (%) for C_15_H_20_N_2_: C 78.90, H 8.83, N 12.27; found: C 78.88, H 8.79, N 12.35.

#### 5-Methoxy-1-(Piperidin-1-Ylmethyl)-1H-Indole (13)

Obtained from 5-methoxyindole and piperidine. R*f* = 0.50 (acetate/n-hexane 2/3). (Yield = 183.0 mg, 75%). ^1^H NMR (CDCl_3_, 400 MHz): δ: 1.25–1.31 (m, 2H, C*H*_2_ piperidine); 1.46–1.52 (m, 4H, C*H*_2_ piperidine); 2.43 (t, 4H, C*H*_2_ piperidine, *J* = 5.2 Hz); 3.78 (s, 3H, C*H*_3_); 4.72 (s, 2H, C*H*_2_); 6.34 (d, 1H, aryl, *J* = 3.0 Hz); 6.79 (dd, 1H, aryl, *J*_1_ = 2,4 Hz; *J*_2_ = 6.5 Hz); 7.00 (d, 1H, aryl, *J* = 2.4 Hz); 7.03 (d, 1H, aryl, *J* = 3.0 Hz); 7.29 (d, 1H, aryl, *J* = 8.9 Hz). ^13^C NMR (CDCl_3_, 100 MHz) δ: 23.9; 25.8; 51.8; 55.8; 68.8; 100.8; 102.3; 110.9; 111.9; 128.9; 129.3; 132.4; 154.0. HR-MS *m/z*: calcd for C_15_H_21_N_2_O, [(M+H)^+^]: 245.1648; found 245.1655. Elemental analysis calcd (%) for C_15_H_20_N_2_O: C 73.74, H 8.25, N 11.47, O 6.55; found: C 73.77, H 8.19, N 11.53, O 6.59.

#### Tert-Butyl((1-(Piperidin-1-Ylmethyl)-1H-Indol-5-yl)Methyl)Carbamate (14)

Obtained from tert-butyl ((1H-indol-5-yl)methyl)carbamate and piperidine. R*f* = 0.35 (dichlorometane/acetate 9/). (Yield = 240.2 mg, 70%). ^1^H NMR (CDCl_3_, 400 MHz): δ: 1.28 (bs, 2H, C*H*_2_ piperidine); 1.40 (s, 9H, C*H*_3_); 1.49 (bs, 4H, C*H*_2_ piperidine); 2.43 (bs, 4H, C*H*_2_ piperidine); 4.33 (d, 2H, C*H*_2_, *J* = 2.9 Hz); 4.75 (s, 2H, C*H*_2_); 6.39 (bs, 1H, aryl); 7.05–7.07 (m, 2H, aryl); 7.35 (d, 1H, aryl, *J* = 8.2 Hz); 7.45 (s, 1H, aryl). ^13^C NMR (CDCl_3_, 100 MHz) δ: 23.9; 25.8; 28.4; 51.8; 68.7; 101.3; 110.4; 119.9; 121.8; 128.6; 129.4; 129.9; 136.5; 155.9. HR-MS *m/z*: calcd for C_20_H_30_N_3_O_2_, [(M+H)^+^]: 344.2333; found 344.2340. Elemental analysis calcd (%) for C_20_H_29_N_3_O_2_: C 69.94, H 8.51, N 12.23, O 9.32; found: C 69.99, H 8.45, N 12.18, O 9.37.

#### Tert-Butyl (2-(1-(Piperidin-1-Ylmethyl)-1H-Indol-3-yl)Ethyl)Carbamate (15)

Obtained from tert-butyl (2-(1H-indol-3-yl)ethyl)carbamate and piperidine. R*f* = 0.35 (ethyl acetate/n-hexane 2/1). (Yield% = 221.5 mg, 62%). ^1^H NMR (CDCl_3_, 400 MHz): δ: 1.37–1.40 (m, 2H, C*H*_2_ piperidine); 1.47 (s, 9H, C*H*_3_); 1.56–1.62 (m, 4H, C*H*_2_ piperidine); 2.54 (bs, 4H, C*H*_2_ piperidine); 2.97 (t, 2H, C*H*_2_, *J* = 6.2 Hz); 3.48 (bs, 2H, C*H*_2_); 4.64 (s, 1H, NH); 4.82 (s, 2H, C*H*_2_); 7.01 (s, 1H, aryl); 7.13 (t, 1H, aryl, *J* = 7.6 Hz); 7.24 (t, 1H, aryl, *J* = 8.0 Hz); 7.46 (d, 1H, aryl, *J* = 8.1 Hz); 7.61 (d, 1H, aryl. *J* = 7.7 Hz). ^13^C NMR (CDCl_3_, 100 MHz) δ: 23.9; 25.8; 28.4; 51.8; 68.5; 110.2; 111.9; 118.8; 119.1; 121.8; 126.8; 127.9; 137.6; 156.0. HR-MS *m/z*: calcd for C_21_H_31_N_3_O_2_, [(M+H)^+^]: 358.2489; found 358.2492. Elemental analysis calcd (%) for C_21_H_31_N_3_O_2_: C 70.55, H 8.74, N 11.75, O 8.95; found: C 70.43, H 8.69, N 11.74, O 9.01.

#### 2-Methyl-1-(Piperidin-1-Ylmethyl)-1H-Indole (16)

R*f* = 0.40 (dichloromethane). (Yield = 50.4 mg, 22%). ^1^H NMR (CDCl_3_, 400 MHz): δ: 1.35 (bs, 2H, C*H*_2_ piperidin); 1.44–1.45 (m, 4H, C*H*_2_ piperidin); 2.39 (bs, 7H, C*H*_2_ piperidin and C*H*_3_); 4.53 (s, 2H, C*H*_2_); 6.17 (s, 1H, aryl); 7.00 (t, 1H, aryl, *J* = 7.5 Hz); 7.04 (d, 1H, aryl, *J* = 7.0 Hz); 7.32 (d, 1H, aryl, *J* = 8.0 Hz); 7.42 (d, 1H, aryl, *J* = 7.6 Hz). ^13^C NMR (CDCl_3_, 100 MHz) δ: 13.0; 24.4; 25.7; 51.9; 65.7; 100.7; 109.8; 119.3; 119.4; 120.4; 128.0; 137.5; 138.0. HR-MS *m/z*: calcd for C_15_H_21_N_2_, [(M+H)^+^]: 229.1699; found 229.1708. Elemental analysis calcd (%) for C_14_H_18_N_2_: C 78.90, H 8.83, N 12.27; found: C 78.81, H 8.85, N 12.45.

#### Bis(2-Methyl-1H-Indol-1-yl)Methane (17)

R*f* = 0.70 (dichloromethane). (Yield = 85.0 mg, 31%). ^1^H NMR (CDCl_3_, 400 MHz): δ: 2.30 (s, 6H, C*H*_3_); 6.31–6.33 (m, 4H, C*H*_2_ and aryl); 7.10–7.12 (m, 4H, aryl); 7.19–7.21 (m, 2H, aryl); 7.54–7.56 (m, 2H, aryl). ^13^C NMR (CDCl_3_, 100 MHz) δ: 13.3; 52.7; 102.5; 109.2; 120.0; 121.4; 128.4; 136.4; 137.0. Elemental analysis calcd (%) for C_19_H_18_N_2_: C 83.18, H 6.61, N 10.21; found: C 83.256, H 6.48, N 10.55.

#### 1-((1H-Pyrrol-1-yl)Methyl)Piperidine (18)

Obtained from pyrrole and piperidine. R*f* = 0.40 (ethyl acetate/n-hexane 1/3). (Yield = 99.0 mg, 74%). ^1^H NMR (CDCl_3_, 400 MHz): δ: 1.38–1.41 (m, 2H, C*H*_2_ piperidine *J* = 4.1 Hz); 1.57–1.63 (m, 4H, C*H*_2_ piperidine); 2.49 (t, 4H, C*H*_2_ piperidine *J* = 4.8 Hz); 4.65 (s, 2H, C*H*_2_); 6.18 (bs, 2H, aryl); 6.70 (bs, 2H, aryl). ^13^C NMR (CDCl_3_, 100 MHz) δ: 23.8; 25.9; 51.3; 71.8; 107.8; 121.6. HR-MS *m/z*: calcd for C_10_H_17_N_2_, [(M+H)^+^]: 165.1386; found 165.1392. Elemental analysis calcd (%) for C_10_H_16_N_2_: C 73.13, H 9.82, N 17.06, O 8.95; found: C 73.08, H 9.90, N 17.10.

#### 9-(Piperidin-1-Ylmethyl)-9H-Carbazole (19)

Obtained from carbazole and piperidine. R*f* = 0.40 (ethyl acetate/n-hexane 1/3). (Yield = 235.1 mg, 89%). ^1^H NMR (CDCl_3_, 400 MHz): δ: 1.43 (bs, 2H, C*H*_2_ piperidine); 1.57–1.63 (m, 4H, C*H*_2_ piperidine); 2.64 (bs, 4H, C*H*_2_ piperidine); 4.97 (s, 2H, C*H*_2_); 7.28 (t, 2H, aryl, *J* = 7.2 Hz); 7.49 (t, 2H, aryl*, J* = 7.2 Hz); 7.59 (d, 2H, aryl, *J* = 8.2 Hz); 8.13 (d, 2H, aryl, *J* = 8.2 Hz). ^13^C NMR (CDCl_3_, 100 MHz) δ: 24.2; 25.8; 52.3; 66.1; 109.8; 119.2; 120.1; 123.1; 125.7; 141.5. HR-MS *m/z*: calcd for C_18_H_21_N_2_, [(M+H)^+^]: 265.1699; found 265.1706. Elemental analysis calcd (%) for C_18_H_20_N_2_: C 81.78, H 7.63, N 10.60; found: C 81.88, H 7.60, N 10.66.

#### Bis(1H-Benzo[d]Imidazol-1-yl)Methane (20)

Obtained from benzimidazole and piperidine. R*f* = 0.30 (ethyl acetate/methanol 5/1). (Yield = 94.3 mg, 38%). ^1^H NMR (CDCl_3_, 400 MHz): δ: 6.43 (s, 2H, C*H*_2_); 7.24–7.29 (m, 4H, aryl); 7.36–7.38 (m, 2H, aryl); 7.74–7.77 (m, 2H, aryl); 8.09 (s, 2H, aryl). ^13^C NMR (CDCl_3_,100 MHz) δ: 53.5; 109.2; 121.1; 123.4; 124.3; 132.7; 142.1; 143.9. Elemental analysis calcd (%) for C_15_H_12_N_4_: C 72.56, H 4.87, N 22.57; found: C 72.48, H 4.93, N 22.54.

#### 2-(Piperidin-1-Ylmethyl)Phenol (21)

Obtained from phenol and piperidine. R*f* = 0.40 (dichlorometane/methanol 9/1). (Yield = 105.0 mg, 55%). ^1^H NMR (CDCl_3_, 400 MHz): δ: 1.42 (bs, 2H, C*H*_2_ piperidine); 1.55–1.59 (m, 4H, C*H*_2_ piperidine); 2.43 (bs, 4H, C*H*_2_ piperidine); 3.59 (s, 2H, C*H*_2_); 6.69 (t, 1H, aryl, *J* = 7.4 Hz); 6.73 (d, 1H, aryl, *J* = 8.0 Hz); 6.88 (d, 1H, aryl, *J* = 7.2 Hz); 7.08 (t, 1H, aryl, *J* = 7.6 Hz). ^13^C NMR (CDCl_3_, 100 MHz) δ: 24.0; 25.9; 53.9; 62.2; 116.0; 118.9; 121.7; 128.4; 158.1. HR-MS *m/z*: calcd for C_12_H_18_NO, [(M+H)^+^]: 192.1383; found 192.1389. Elemental analysis calcd (%) for C_12_H_17_NO: C 75.35, H 8.96, N 7.32, O 8.36; found: C 75.40, H 9.02, N 7.27, O 8.30.

#### 2-Methyl-6-(Piperidin-1-Ylmethyl)Phenol (22)

Obtained from o-cresol and piperidine. R*f* = 0.40 (n-hexane/ethere 2/1). (Yield = 119.0 mg, 58%). ^1^H NMR (CD_3_OD, 400 MHz): δ: 1.46 (bs, 2H, C*H*_2_ piperidine); 1.57–1.61 (m, 4H, C*H*_2_ piperidine); 2.09 (s, 3H, C*H*_3_); 2.62 (bs, 4H, C*H*_2_ piperidine); 3.73 (s, 2H, C*H*_2_); 6.61 (t, 1H, aryl, *J* = 7.5 Hz); 6.82 (d, 1H, aryl, *J* = 7.3 Hz); 6.95 (d, 1H, aryl, *J* = 7.4 Hz). ^13^C NMR (CD_3_OD, 100 MHz) δ: 14.6; 23.0; 24.8; 53.2; 59.9; 118.8; 119.6; 124.4; 127.1; 130.3; 155.3. HR-MS *m/z*: calcd for C_13_H_20_NO, [(M+H)^+^]: 206.1539; found 206.1543. Elemental analysis calcd (%) for C_13_H_19_NO: C 76.06, H 9.33, N 6.82, O 7.79; found: C 76.00, H 9.39, N 6.75, O 7.87.

#### 4-Methyl-2-(Piperidin-1-Ylmethyl)Phenol (23)

Obtained from p-cresol and piperidine. R*f* = 0.40 (ethyl acetate/n-hexane 1/3). (Yield = 125.1 mg, 61%). ^1^H NMR (CDCl_3_, 400 MHz): δ: 1.42 (bs, 2H, C*H*_2_ piperidine); 1.54–1.58 (m, 4H, C*H*_2_ piperidine); 2.16 (s, 3H, C*H*_3_); 2.43 (bs, 4H, C*H*_2_ piperidine); 3.55 (s, 2H, C*H*_2_); 6.63 (d, 1H, aryl, *J* = 8.1 Hz); 6.69 (s, 1H, aryl); 6.88 (d, 1H, aryl, *J* = 8.0 Hz). ^13^C NMR (CDCl_3_, 100 MHz) δ: 20.4; 24.0; 25.9; 53.9; 62.2; 115.7; 121.3; 127.9; 128.9; 155.7. HR-MS *m/z*: calcd for C_13_H_20_NO, [(M+H)^+^]: 206.1539; found 206.1547. Elemental analysis calcd (%) for C_13_H_19_NO: C 76.06, H 9.33, N 6.82, O 7.79; found: C 76.14, H 9.36, N 6.87, O 7.70.

#### 2,4-Dichloro-6-(Piperidin-1-Ylmethyl)Phenol (24)

Obtained from 2,4-dichlorophenol and piperidine. R*f* = 0.50 (ethyl acetate/n-hexane 1/3). (Yield = 108.8 mg, 42%). ^1^H NMR (CD_3_OD, 400 MHz): δ: 1.46–1.50 (m, 2H, C*H*_2_ piperidine); 1.59–1.64 (m, 4H, C*H*_2_ piperidine); 2.62 (bs, 4H, C*H*_2_ piperidine); 3.75 (s, 2H, C*H*_2_); 6.91 (d, 1H, aryl, *J* = 2.5 Hz); 7.16 (d, 1H, aryl, *J* = 2.5 Hz). ^13^C NMR (CD_3_OD, 100 MHz) δ: 22.9; 24.8; 52.9; 60.0; 121.6; 123.0; 127.2; 128.3; 154.6. HR-MS *m/z*: calcd for C_12_H_16_Cl_2_NO, [(M+H)^+^]: 260.0603; found 260.0608. Elemental analysis calcd (%) for C_12_H_15_Cl_2_NO: C 55.40, H 5.81, Cl 27.25, N 5.38, O 6.15; found: C 55.44, H 5.74, Cl 27.30, N 5.43, O 6.11.

#### 2-(Tert-Butyl)-4-(Piperidin-1-Ylmethyl)Phenol (25)

Obtained from o-tert-butylphenol and piperidine. R*f* = 0.50 (ethyl acetate/n-hexane 1/3). (Yield% = 163.0 mg, 66%). ^1^H NMR (CD_3_OD, 400 MHz): δ: 1.29 (s, 9H, C*H*_3_); 1.44–1.50 (m, 2H, C*H*_2_ piperidine); 1.60–1.66 (m, 4H, C*H*_2_ piperidine); 2.73 (bs, 4H, C*H*_2_ piperidine); 3.72 (s, 2H, C*H*_2_); 6.65 (d, 1H, aryl, *J* = 8.1 Hz); 6.95 (d, 1H, aryl, *J* = 8.1 Hz); 7.15 (s, 1H, aryl). ^13^C NMR (CD_3_OD, 100 MHz) δ: 22.5; 23.7; 28.5; 34.2; 52.9; 61.8; 115.8; 122.1; 129.1; 136.2; 157.0. HR-MS *m/z*: calcd for C_16_H_26_NO, [(M+H)^+^]: 248.2009; found 248.2013. Elemental analysis calcd (%) for C_16_H_25_NO: C 77.68, H 10.19, N 5.66, O 6.47; found: C 77.72, H 10.22, N 5.63, O 6.50.

#### Bis(2-(Tert-Butyl)Phenoxy)Methane (26)

Obtained from o-tert-butylphenol and piperidine. R*f* = 0.65 (ethyl acetate/n-hexane 1/3). (Yield = 34.3 mg, 11%). ^1^H NMR (CDCl_3_, 400 MHz): δ: 1.47 (s, 18H, C*H*_3_); 5.87 (s, 2H, C*H*_2_); 7.00–7.04 (m, 2H, aryl); 7.22–7.26 (m, 2H, aryl); 7.32 (d, 2H, aryl, *J* = 7.2 Hz); 7.37 (dd, 2H, aryl, *J*_1_ = 6.4 Hz; *J*_2_ = 1.4 Hz). ^13^C NMR (CDCl_3_, 100 MHz) δ: 30.1; 34.9; 91.5; 114.8; 122.0; 126.9; 127.2; 138.6; 156.5. Elemental analysis calcd (%) for C_21_H_28_O_2_: C 80.73, H 9.03, O 10.24; found: C 80.59, H 9.12, O 10.28.

#### Bis(2,6-Diisopropylphenoxy)Methane (27)

Obtained from 2,6-diisopropylphenol and piperidine. R*f* = 0.55 (ethyl acetate/n-hexane 1/4). (Yield = 246.7 mg, 67%). ^1^H NMR (CDCl_3_, 400 MHz): δ: 1.24 (d, 24H, C*H*_3_, *J* = 6.8 Hz); 3.40–3.47 (m, 4H, C*H*); 5.26 (s, 2H, C*H*_2_); 7.18 (s, 6H, aryl). ^13^C NMR (CDCl_3_,100 MHz) δ: 23.9; 26.7; 100.8; 124.0; 125.2; 142.2; 151.0. Elemental analysis calcd (%) for C_25_H_36_O_2_: C 81.47, H 9.85, O 8.68; found: C 81.60, H 9.79, O 8.59.

#### 2-(Piperidin-1-Ylmethyl)Naphthalen-1-ol (28)

Obtained from 1-naphthol and piperidine. R*f* = 0.50 (ethyl acetate/n-hexane 1/5). (Yield = 217.0 mg, 90%). ^1^H NMR (CDCl_3_, 400 MHz): δ: 1.45 (bs, 2H, C*H*_2_ piperidine); 1.59–1.62 (m, 4H, C*H*_2_ piperidine); 2.50 (bs, 4H, C*H*_2_ piperidine); 3.74 (s, 2H, C*H*_2_); 6.98 (d, 1H, aryl, *J* = 8.2 Hz); 7.20 (d, 1H, aryl, *J* = 8.5 Hz); 7.35–7.37 (m, 2H, aryl); 7.67 (d, 1H, aryl, *J* = 5.2 Hz); 8.16 (d, 1H, aryl, *J* = 5.2 Hz). ^13^C NMR (CDCl_3_, 100 MHz) δ: 24.1; 25.9; 62.4; 113.9; 118.0; 122.0; 124.7; 125.0; 125.8; 126.5; 127.3; 133.8; 153.8. HR-MS *m/z*: calcd for C_16_H_20_NO, [(M+H)^+^]: 242.1539; found 242.1546. Elemental analysis calcd (%) for C_16_H_19_NO: C 79.63, H 7.94, N 5.80, O 6.63; found: C 79.70, H 8.03, N 5.72, O 6.59.

#### 1-(Piperidin-1-Ylmethyl)Naphthalen-2-ol (29)

Obtained from 2-naphthol and piperidine. R*f* = 0.50 (ethyl acetate/n-hexane 1/5). (Yield = 226.7 mg, 94%). ^1^H NMR (CDCl_3_, 400 MHz): δ: 1.46 (bs, 2H, C*H*_2_ piperidine); 1.61–1.65 (m, 4H, C*H*_2_ piperidine); 2.52 (bs, 4H, C*H*_2_ piperidine); 4.05 (s, 2H, C*H*_2_); 7.01 (d, 1H, aryl, *J* = 8.8 Hz); 7.21 (d, 1H, aryl, *J* = 7.8 Hz); 7.35 (t, 1H, aryl, *J* = 8.1 Hz); 7.60 (d, 1H, aryl, *J* = 8.8 Hz); 7.67 (d, 1H, aryl, *J* = 8.0 Hz); 7.72 (d, 1H, aryl, *J* = 8.6 Hz). ^13^C NMR (CDCl_3_, 100 MHz) δ: 23.9; 25.8; 57.1; 111.0; 119.3; 120.9; 122.3; 126.2; 128.4; 128.9; 129.1; 132.7; 156.8. HR-MS *m/z*: calcd for C_16_H_20_NO, [(M+H)^+^]: 242.1539; found 242.1548. Elemental analysis calcd (%) for C_16_H_19_NO: C 79.63, H 7.94, N 5.80, O 6.63; found: C 79.68, H 8.00, N 5.83, O 6.67.

#### 6-(Piperidin-1-Ylmethyl)Isoquinolin-5-ol (30)

Obtained from isoquinolin-5-ol and piperidine. R*f* = 0.40 (ethyl acetate/n-hexane 1/3). (Yield = 210.7 mg, 87%). ^1^H NMR (CDCl_3_, 400 MHz): δ: 1.47 (bs, 2H, C*H*_2_ piperidine); 1.61–1.63 (m, 4H, C*H*_2_ piperidine); 2.51 (bs, 4H, C*H*_2_ piperidine); 3.78 (s, 2H, C*H*_2_); 7.10 (d, 1H, aryl, *J* = 8.0 Hz); 7.31 (d, 1H, aryl, *J* = 8.0 Hz); 7.91 (d, 1H, aryl, *J* = 8.0 Hz); 8.41 (d, 1H, aryl, *J* = 8.0 Hz); 9.07 (s, 1H, aryl). ^13^C NMR (CDCl_3_, 100 MHz) δ: 23.9; 25.8; 62.3; 115.1; 117.4; 118.1; 127.6; 127.8; 128.9; 142.0; 151.8; 153.2. HR-MS *m/z*: calcd for C_15_H_19_N_2_O, [(M+H)^+^]: 243.1492; found 243.1500. Elemental analysis calcd (%) for C_15_H_18_N_2_O: C 74.35, H 7.49, N 11.56, O 6.60; found: C 74.41, H 7.62, N 11.48, O 6.65.

#### Bis(Phenylthio)Methane (31)

Obtained from thiophenol and piperidine. R*f* = 0.40 (n-hexane). (Yield% = 92.8 mg, 40%). ^1^H NMR (CDCl_3_, 400 MHz): δ: 4.38 (s, 2H, C*H*_2_); 7.28 (t, 2H, aryl, *J* = 7.1 Hz); 7.33 (t, 4H, aryl, *J* = 7.2 Hz); 7.46 (d, 4H, aryl, *J* = 7.5 Hz). ^13^C NMR (CDCl_3_,100 MHz) δ: 40.7; 127.2; 129.0; 130.8; 135.0. Elemental analysis calcd (%) for C_13_H_12_S_2_: C 67.20, H 5.21, S 27.60; found: C 67.33, H 5.18, S 27.79.

## Results and Discussion

When we performed N-methylation reactions of non-substituted indole using CH_3_I in sodium hydride/DCM/DMF solution assisted by ultrasound irradiation (US), we observed the almost exclusive formation of 1-diindolylmethane (86% of yield), which suggested that DCM is a bridging agent in the formation of this N-aminomethylated compound (Mills et al., 1987, 2009; Matsumoto et al., 1993; Souquet et al., 2006; Rudine et al., 2010; Zhou et al., 2011). In an attempt to capitalize on DCM behavior, we introduced a secondary amine, specifically piperidine, in the reaction. In this case, we observed the formation of 1-diindolylmethane **2** and -(piperidin-1-ylmethyl)-1H-indole **3**, which were isolated in yields of 40 and 51%, respectively. Here we describe an efficient approach to the synthesis of 1-indolyl methanamines, starting from different indole substrates and amines under basic conditions.

An initial analysis of time, temperature, and irradiation conditions (Table 1) for the reaction of indole with piperidine, in DMF/DCM (1/4; 5 mL) using 2.0 equiv. of NaH, confirmed the efficacy of operating ultrasound (Baig and Varma, 2012; Cravotto et al., 2013) as reaction catalyst, and 2 h at 50°C as optimal time reaction and temperature conditions (Table 1). However, under these conditions we did not observe any selectivity vs. the formation of **3** (**2/3** ratio in the range 1–2, entries 1–8, Table 1 and entry 1, Table 2). When we increased the reaction time to 3 h (entry 5, Table 1) we observed the progressive formation of the 3-((1H-indol-1-yl)methyl)-1-(piperidin-1-ylmethyl)-1H-indole (**4**), probably as a result of the attack of 1-methylene-1H-indol-1-ium at C-3 position of compound **3** (entry 5, Table 1). An increase of temperature in batch and μW irradiation also favored the formation of **4** (entries 6–8, Table 1).

**Table 2:** Optimization of the conditions for the reaction of **1** with piperidine^a^.

**Entry**	**Solvent**	**Base**	**Yields (%)^**b**^**	
			**2**	**3**
1	DMF^c^	NaH	42	51
2	DMF^c^	n-Bu-Li	–	–
3	DMF^c^	DBU	–	7
4	THF^d^	KHMDS	–	2
5	H_2_O^d^	NaOH/TBAB	5	11
6	CH_3_CN^d^	NaH	37	55
7	CH_3_CN^e^	NaH	33	61
8	CH_3_CN^d^	–	–	16
9	CH_3_CN^d^	Cs_2_CO_3_	–	29
10	CH_3_${\text{COCH}}_{3}^{\text{d}}$	Cs_2_CO_3_	–	23

a *Reaction conditions: indole (1.0 mmol), piperidine (1.5 mmol, 1.5 equiv.), base (2.0 mmol, 2.0 equiv.), 5 mL of solvents, US irradiation, 120 min, 50°C*.

b *Yields were calculated by HPLC*.

c *DMF/DCM ratio = 1/4 (v/v)*.

d *Solvent/DCM ratio = 1/1 (v/v)*.

e *DCM = 3 mmol (3 equiv., 0.192 mL)*.

As shown in Table 2 (entries 2–10), the substitution of NaH by different bases (entries 2–5, 9, and 10) resulted in a strong decrease of **3** yields, while acetonitrile was the solvent of choice (entries 6 and 7). Interestingly, the absence of NaH in CH_3_CN (entry 8) resulted in the aminomethylation of N-1 in a yield of 16%, while Cs_2_CO_3_ did not improve the reaction performance in terms of either CH_3_CN or acetone (entries 9 and 10).

**Scheme 1:**
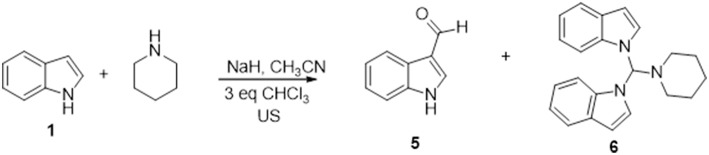
Reaction of 1 with piperidine in the presence of CHCl_3_.

We also found that the 3-(piperidin-1-ylmethyl)-1H-indole regioisomer was not formed under any of the conditions used, which suggests that 1-methylene-1H-indol-1-ium, but not 1-methylenepiperidine-1-ium, is the active intermediate of this reaction (Mills et al., 1987). In fact, 1-methylindole was recovered unchanged when it replaced indole as starting material. The greater reactivity of N-1 vs. C-3 in indoles under the above reaction conditions has been also observed replacing dichloromethane with chloroform. In fact, the reaction of indole in CH_3_CN/3 eq. CHCl_3_ with piperidine, gives both indole-3-aldheyde (**5**) and 1,1'-(piperidin-1-ylmethylene) bis(1H-indole) (**6**) in yields of 11 and 19%, respectively (Scheme 1).

The formation of these products can be explained considering the dichlorocarbene generated from chloroform in basic conditions as electrophilic species (Hine et al., 1953; Saunders and Murray, 1960; Kirmse et al., 1990; Wynberg and Meijer, 2005). The addition of the dichlorocarbene to positions 2 and 3 of indole leads to the well-known Reimer-Tiemann (Wynberg and Meijer, 2005) formylated derivative **5**, while, according to literature (Hine et al., 1953; Saunders and Murray, 1960; Kirmse et al., 1990), compound **6** could be obtained from a halogenated alkyl adduct, which quickly undergoes β-elimination leading to a reactive chloromethylene indolinium intermediate, as shown in Scheme S1. Addition of a nucleophile and regeneration of the indolium species followed by a second nucleophilic attack leads to the major compound **6**.

**CHART 2:**
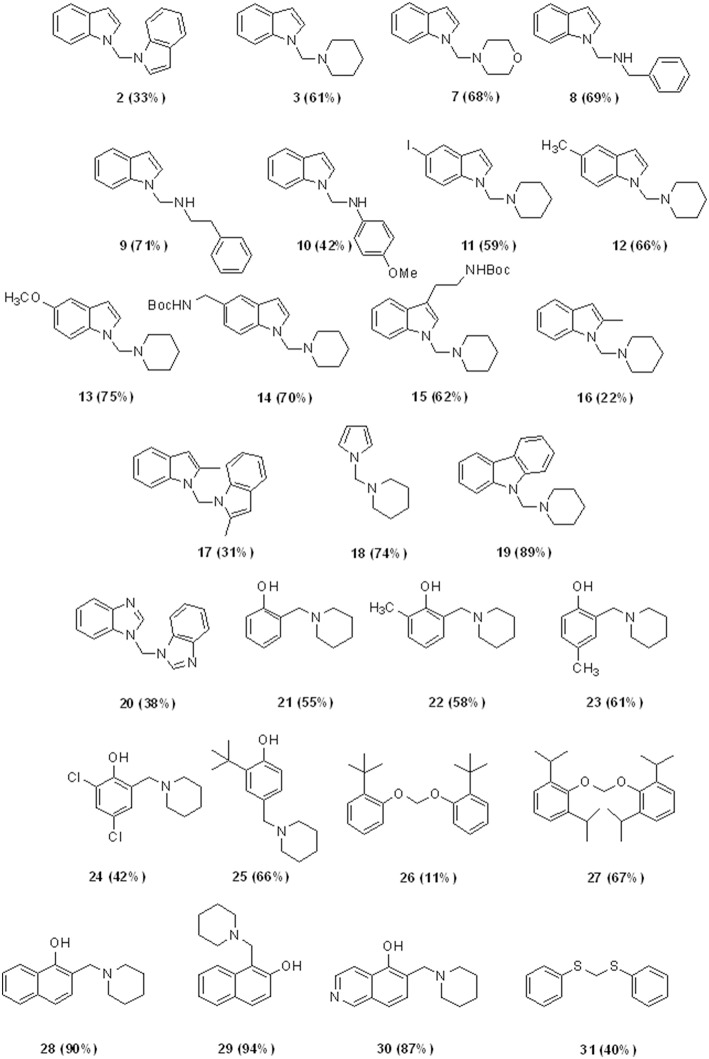
Aminomethylation of Aromatic Compounds with DCM and Amines^a,b^. ^a^Reaction conditions: 3.0 mmol of CH_2_Cl_2_, 1.0 mmol of substrate, 1.5 mmol of amine, 2.0 mmol of NaH, 5 mL of CH_3_CN, under ultrasound irradiation at room temperature for 120 min. ^b^Isolated yield.

Besides these results confirm the halogenated solvents as appropriate C1 sources, the low yields obtained using CHCl_3_ discouraged further investigations. Therefore, we next explored the scope of the reaction using DCM as C1 source under the optimized reaction conditions (entry 7, Table 2), by varying the amine partners, using alkyl, and aryl amines as the second reaction component. Given the incidence of nitrogen heterocycles in chemistry and pharmaceuticals (Vitaku et al., 2014; Blakemore et al., 2018), we used various substituted indoles and other N-heterocycles in combination with piperidine (Chart 2). The reactions of indole with another secondary amine, morpholine, or with primary alkyl and aryl amines such as benzyl and phenylethyl amines resulted in N-((1H-indol-1-yl)methyl) derivatives **7**–**9** in high yields (68–71%, Chart 2).

However, the reaction with anilines can only be performed with anilines containing an electron donor group. Therefore, using 4-methoxy aniline, we obtained the amino methylene derivative **10** in a yield of 42%. Biologically relevant 3- or 5- substituted indoles (Bertamino et al., 2016; Musella et al., 2016) reacted with piperidine to provide the N-aminomethyl derivatives **11–15** in a yield range of 59–75% and high selectivity, especially in the case of indoles substituted with electron donor groups (Table 3).

**Table 3:** Selectivity of the aminomethylation reaction.

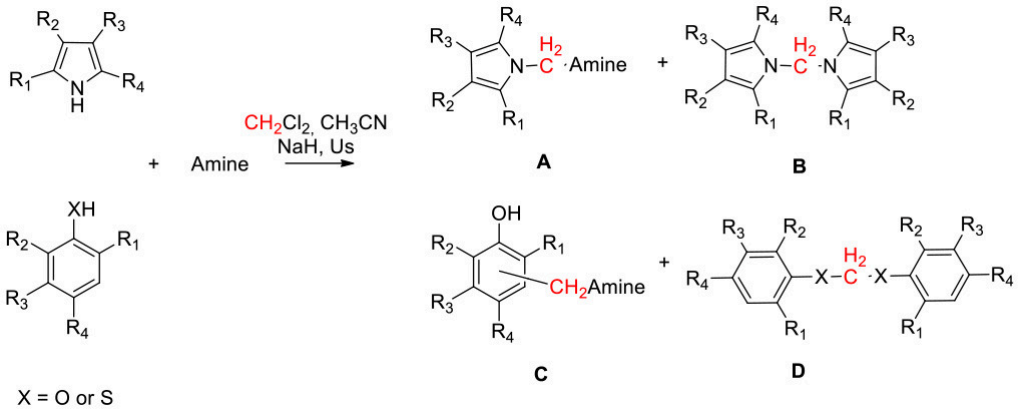			
**Compound**	**A/B or C/D ratio**^**a**^	**Compound**	**A/B or C/D ratio**^**a**^
**7**	9/1	18	1/−
**8**	7/3	19	1/−
**9**	2/1	21	1/−
**10**	7/3	22	1/−
**11**	9/1	23	1/−
**12**	1/−	24	1/−
**13**	9/1	25	9/1
**14**	9/1	28	1/−
**15**	1/−	29	1/−
**16**	2/3	30	1/−

a *The A/B or C/D ratio was calculated as isolated yields*.

Compounds **14** and **15** containing a Boc-protecting group are stable under classical acid deprotection conditions, thus becoming effective intermediates in the synthesis of more complex derivatives. Using 2-methyl indole as starting material, we also obtained the aminomhetylated product (**16**) in 22% of yield, and its related dimeric compound (**17**) in 31% of yield. Next, the reaction of piperidine with pyrrole and carbazole generated a high yield of the corresponding aminomethyl derivatives **18** (74%) and **19** (89%), which were also obtained with high selectivity (Table 3). However, benzoimidazole yielded only bis-benzoimidazolylmethane (**20**, 38%) while pyridine and pyridinol derivatives did not react in our conditions (Mastalir et al., 2017).

Given the chemist community's interest in the chemistry of phenol and its derivatives, in particular for the activation of C-H bonds to generate new C-C bonds (Nair et al., 1994; Joshi et al., 2004; Roman, 2015; Dai et al., 2017; Mastalir et al., 2017), we applied the above described methodology to phenols as well as to other heterocycles namely, 1- and 2-napthol, 5-hydroxyisoquinoline, thiophene, and thiophenol, again using piperidine and DCM as the other two reaction components (Chart 2).

Phenol, 2- and 2,4- electron-donating substituted phenols selectively produced the 2-(piperidin-1-ylmethyl) phenol derivatives **21–23** in a yield range of 55–61%. Reaction with 2,4-dichlorophenol led to 42% of 2,4-dichloro-6-(piperidin-1-ylmethyl)phenol (**24**, Table 3 for the selectivity ratio). Reaction from bulky substituted phenol orto t-butyl phenol gave the expected 2-(tert-butyl)-6-(piperidin-1-ylmethyl)phenol **25** in a yield of 66% and also bis(2-(tert-butyl)phenoxy)methane **26** in a yield of 11%, while with 2,6-di-isopropylphenol the diphenoxymethane derivative **27** was exclusively obtained (Bauerle and Brodbelt, 1995). 1- or 2-naphtol, and 5-isoquinolinol produced selectively the 2-(piperidin-1-ylmethyl)naphthalen-1-ol (**28**), 1-(piperidin-1-ylmethyl)naphthalen-2-ol (**29**), and 8-(piperidin-1-ylmethyl)isoquinolin-5-ol (**30**), in very high yields (90, 94, and 87%, respectively). As we expected, the reaction with thiophene led to degradation products, while the same conditions applied to thiophenol led to bis(phenylthio)methane **31** in a yield of 40%.

## Conclusions

In conclusion, we have developed a practical and sustainable three-component aminomethylation method using different N-heterocycles in combination with a wide range of amines and DCM as C1 source. Thanks to the full N- vs. C- regioselectivity observed in this reaction, this method is an attractive alternative approach to the synthesis of 1-aminomethyl indole derivatives, a class of compounds hitherto poorly accessible. This atom-efficient reaction exploits the potential of ultrasound waves to provide new highly functionalized indoles that are stable both over time and in common synthetic transformations thereby increasing the molecular diversity of this important template. The methodology may also be suitable for other aza-heterocycles, phenols, and some of its derivatives as aryl alcohols, which suggests its potential in the chemistry of materials and medicines, as well as in the life sciences.

## Data Availability

The raw data supporting the conclusions of this manuscript will be made available by the authors, without undue reservation, to any qualified researcher.

## Author Contributions

CO and VD synthesized and characterized compounds. SM, TC, and VV contributed to synthesis and data analysis. GP and FM performed HPLC analysis. PC analyzed data and contributed in conceptualization and writing. IG and AB designed the experiments, analyzed and organized data, and wrote the article.

### Conflict of Interest Statement

The authors declare that the research was conducted in the absence of any commercial or financial relationships that could be construed as a potential conflict of interest.
