# Supplementary material for: An Efficient Approach to Aromatic Aminomethylation Using Dichloromethane as Methylene Source
Source: Front Chem. 2019 Aug 13;7:568. doi: 10.3389/fchem.2019.00568 (PMC6700257; doi:10.3389/fchem.2019.00568)
Supplement: Supplementary file 1 [file Data_Sheet_1.docx]

**SUPPORTING INFORMATIONS**

**An Efficient Approach to Aromatic Aminomethylation Using Dichloromethane as Methylene Source**

Carmine Ostacolo^1,#^, Veronica Di Sarno ^2,#^, Simona Musella^3^, Tania Ciaglia^2^, Vincenzo Vestuto^2^, Giacomo Pepe^2^, Fabrizio Merciai^2^, Pietro Campiglia^2,3^, Isabel M. Gomez Monterrey^1,*^, Alessia Bertamino^2,*^

^1^ Department of Pharmacy, University of Naples “Federico II”, Napoli (NA), Italy

^2^ Department of Pharmacy, University of Salerno, Fisciano (SA), Italy

^3^ Fondazione EBRIS, Salerno (SA), Italy

**1. Scheme S1: Proposed reaction mechanism for the use of CH_2_Cl_2_ (A) and CHCl_3_ (B) as C1 source**

**2. NMR spectra of synthesized compounds**

**Scheme S1:** Proposed reaction mechanism for the reaction involving CH_2_Cl_2_ (**A**) and CHCl_3_ (**B**) as C1 source

**2. NMR spectra**


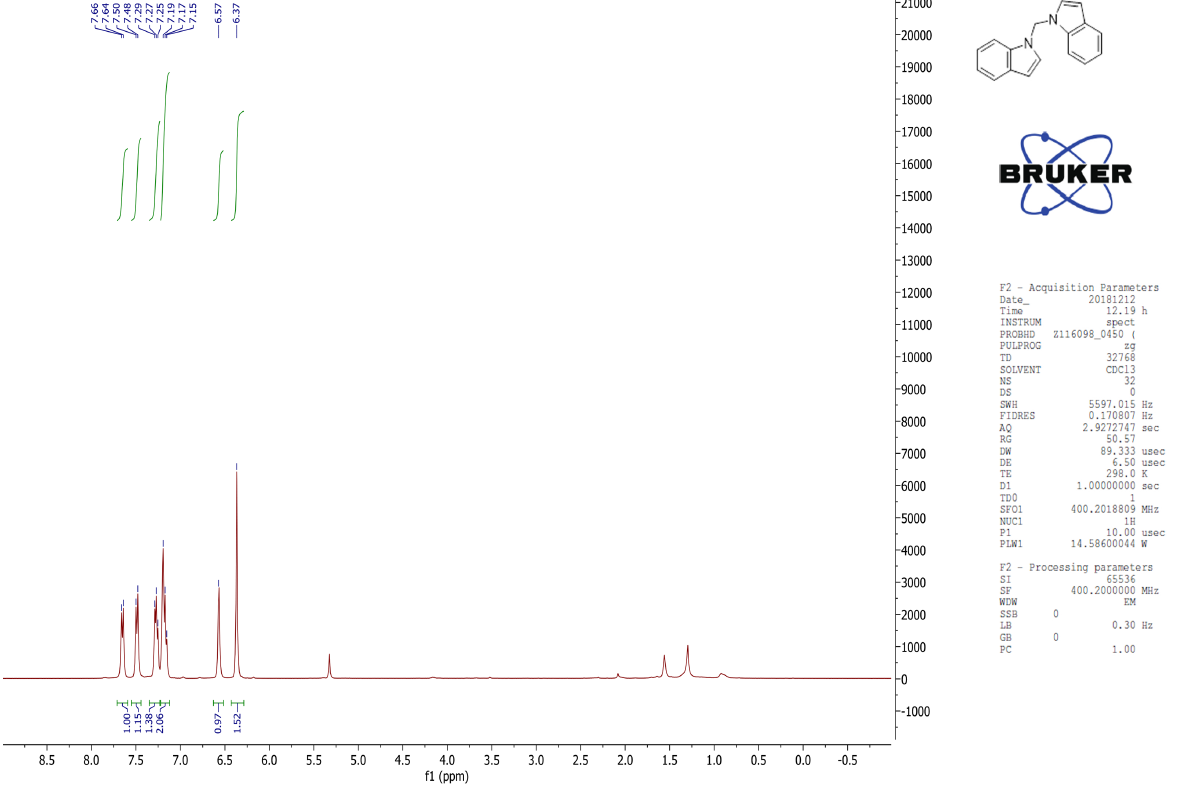


**Figure S1.** ^1^H NMR spectra of **2**


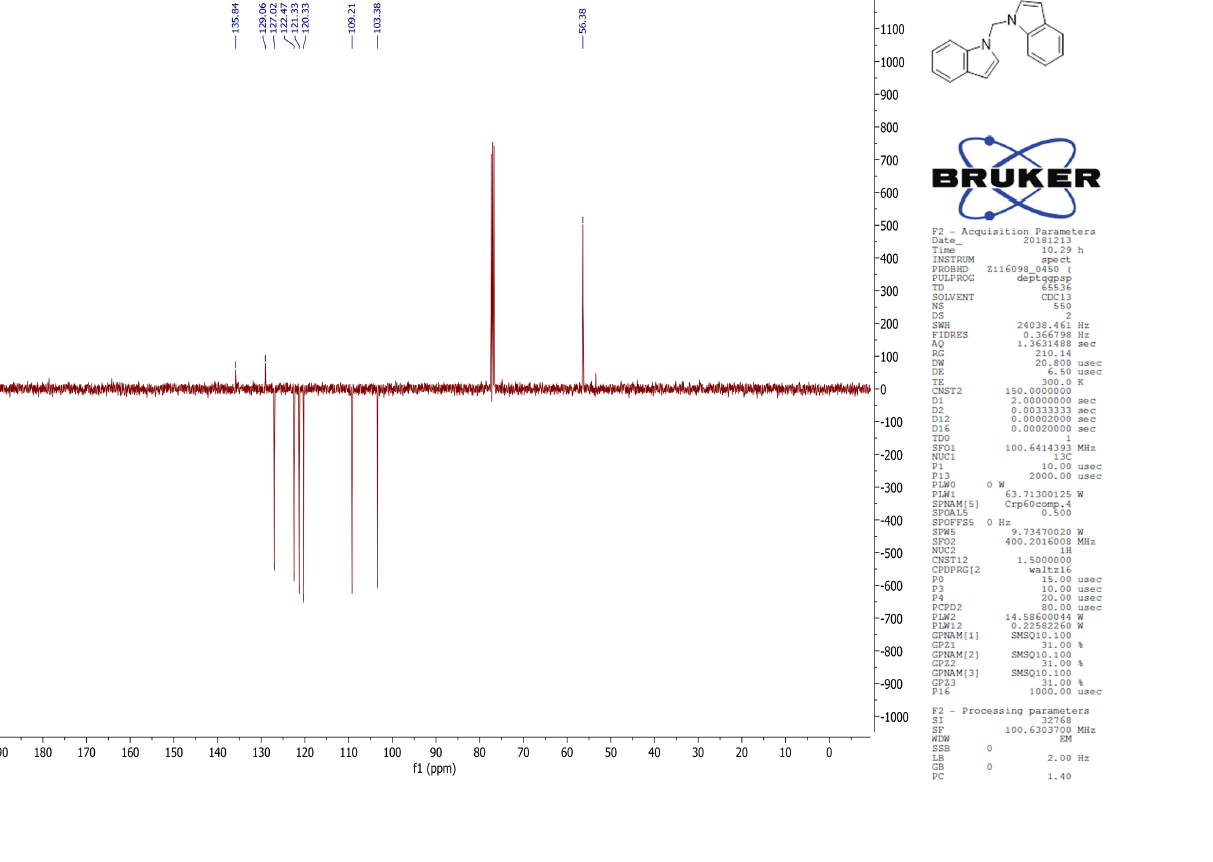


**Figure S2.** qDEPT spectra of **2**


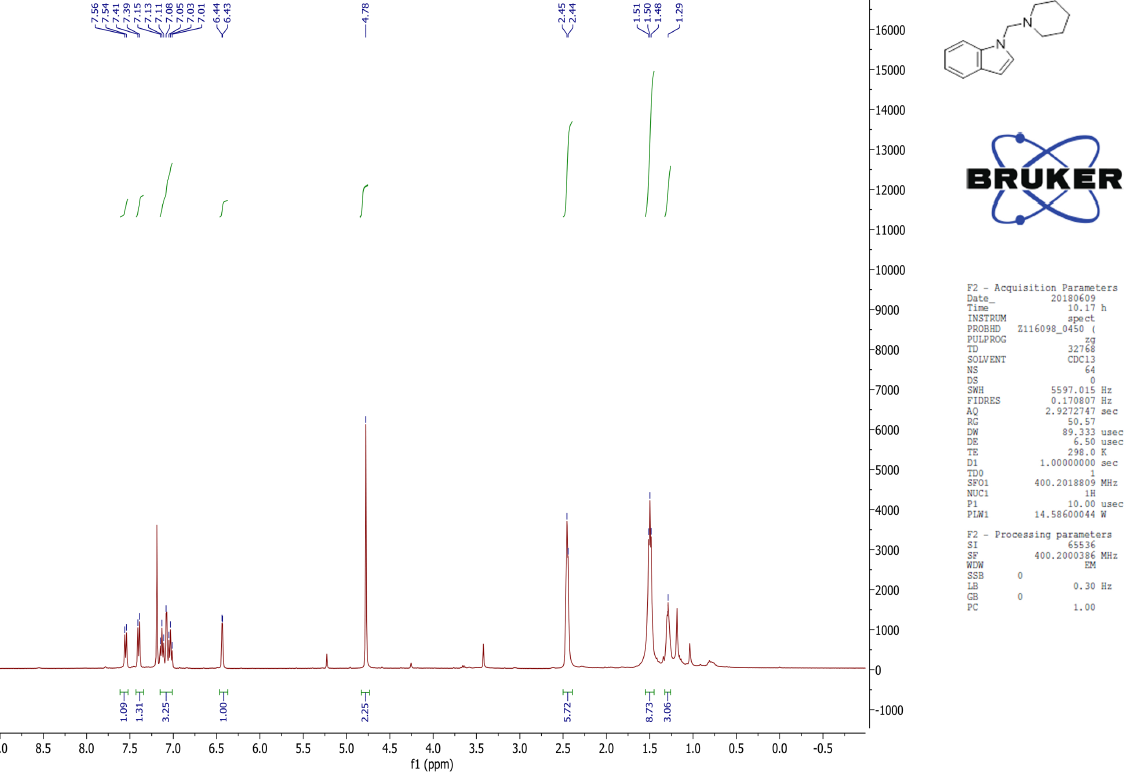


**Figure S3.** ^1^H spectra of **3**


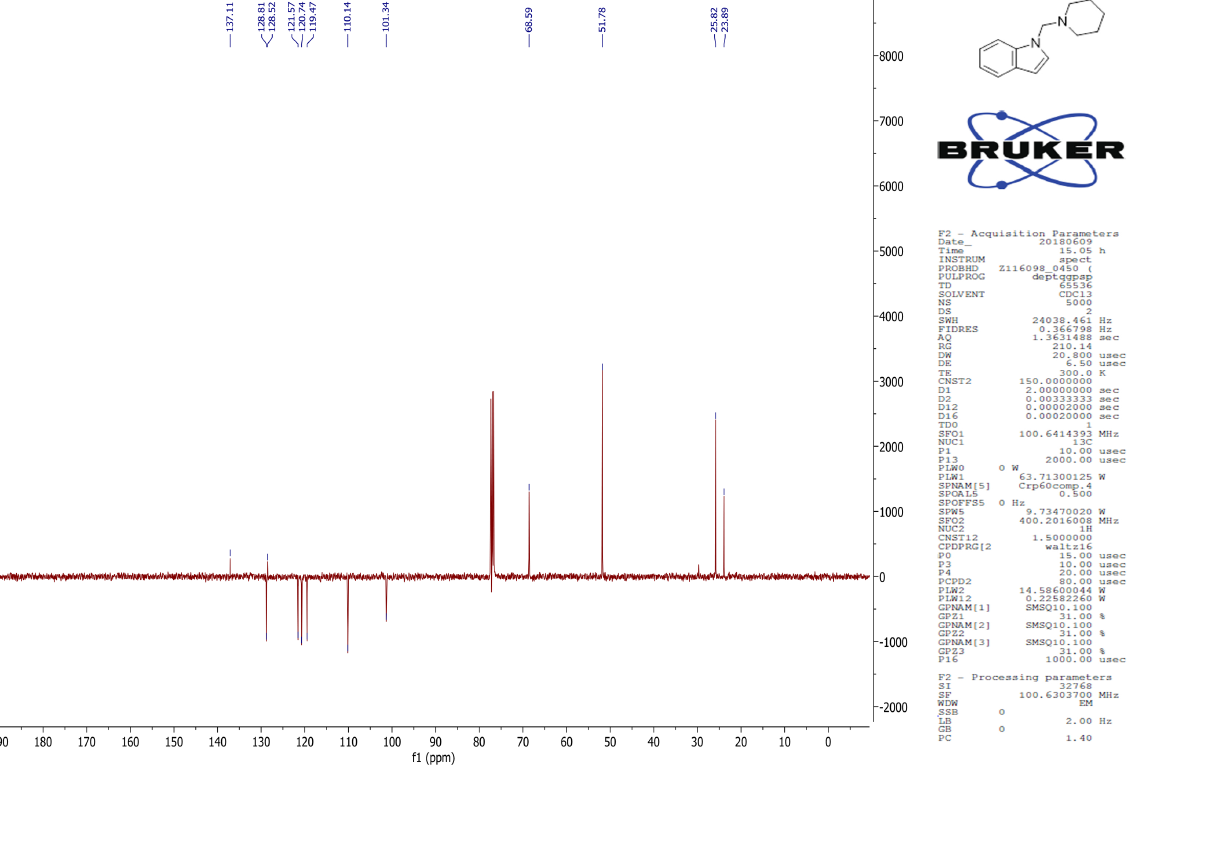


**Figure S4.** qDEPT spectra of **3**


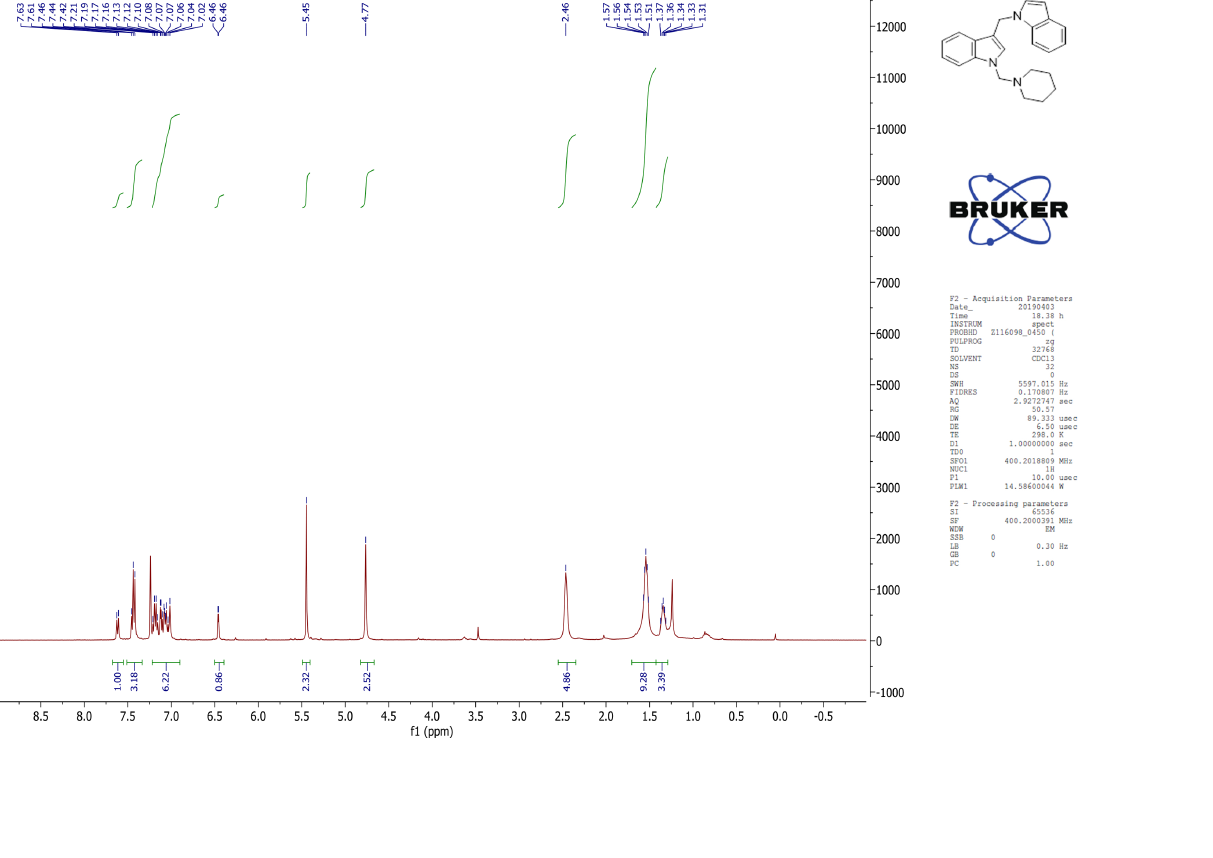


**Figure S5.** ^1^H spectra of **4**


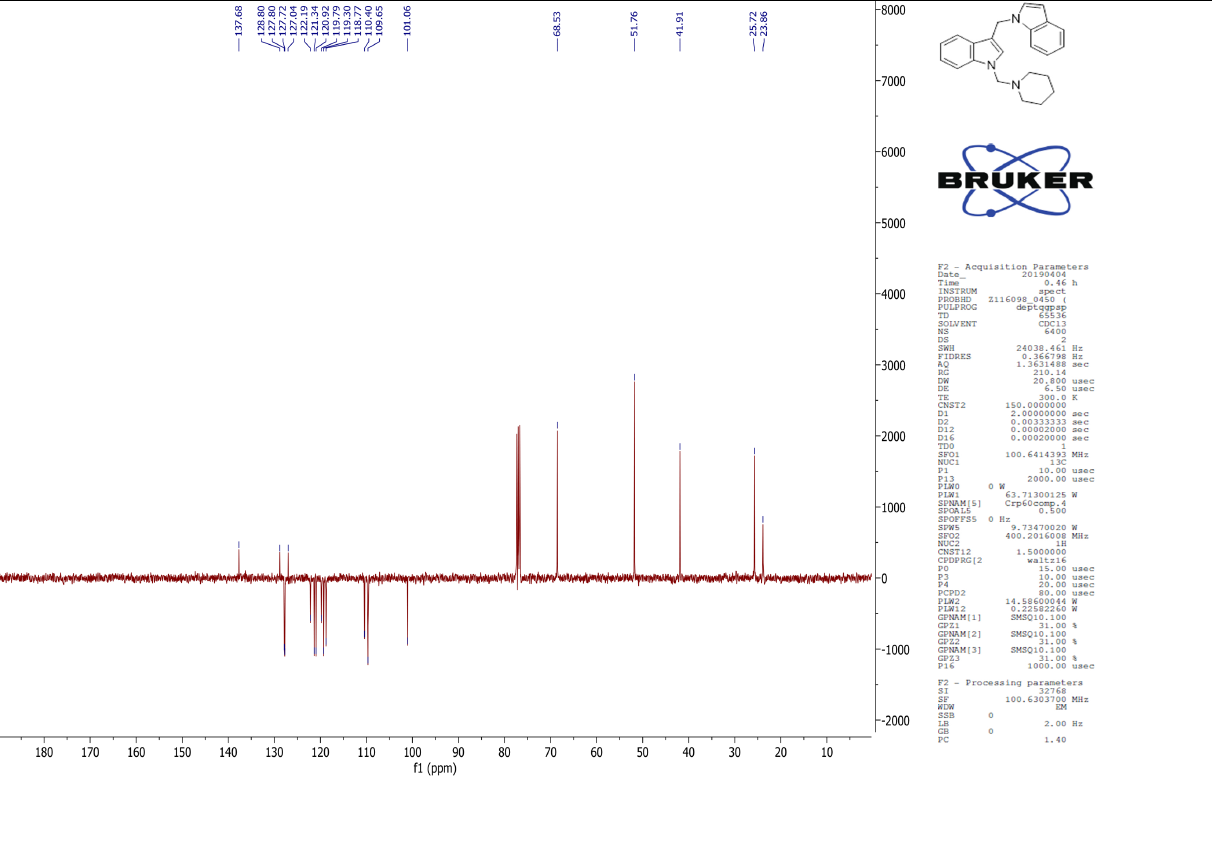


**Figure S6.** qDEPT spectra of **4**


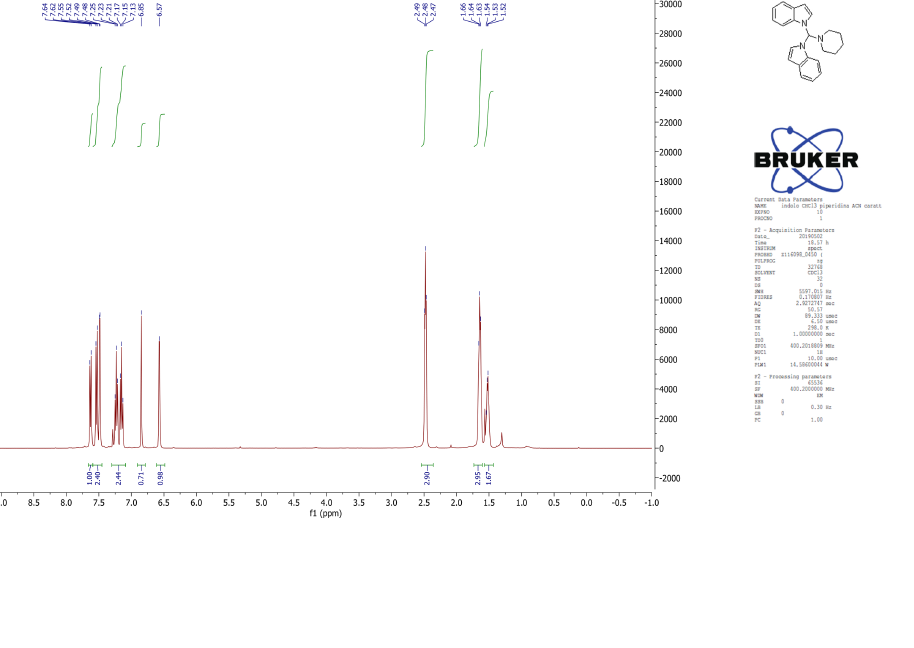


**Figure S7.** ^1^H spectra of **6**


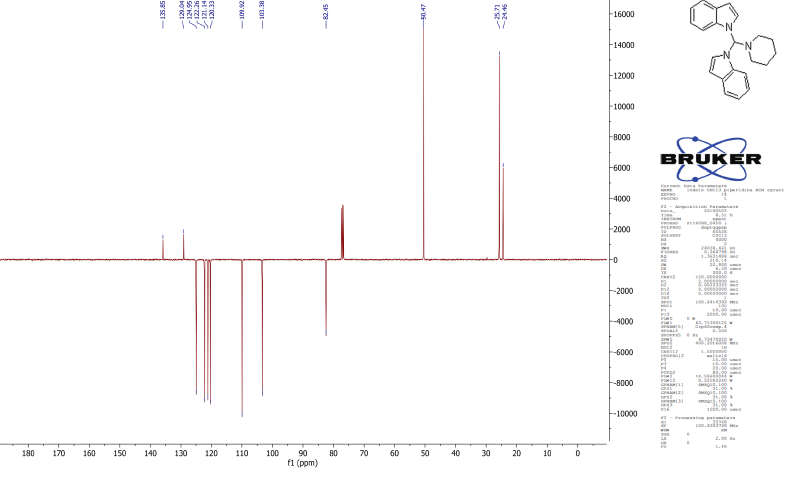


**Figure S8.** qDEPT spectra of **6**

**Figure S9.** HSQC spectra of **6**


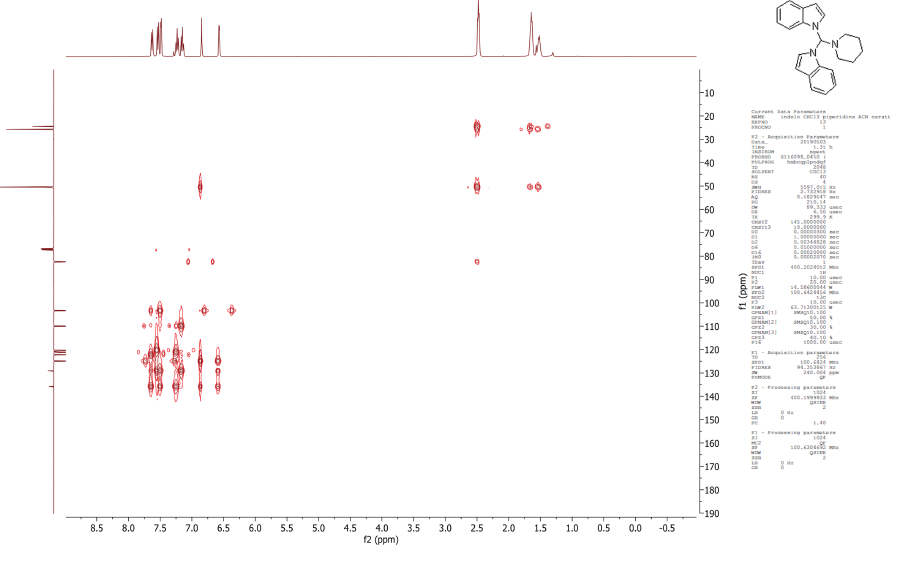


**Figure S10.** HMBC spectra of **6**


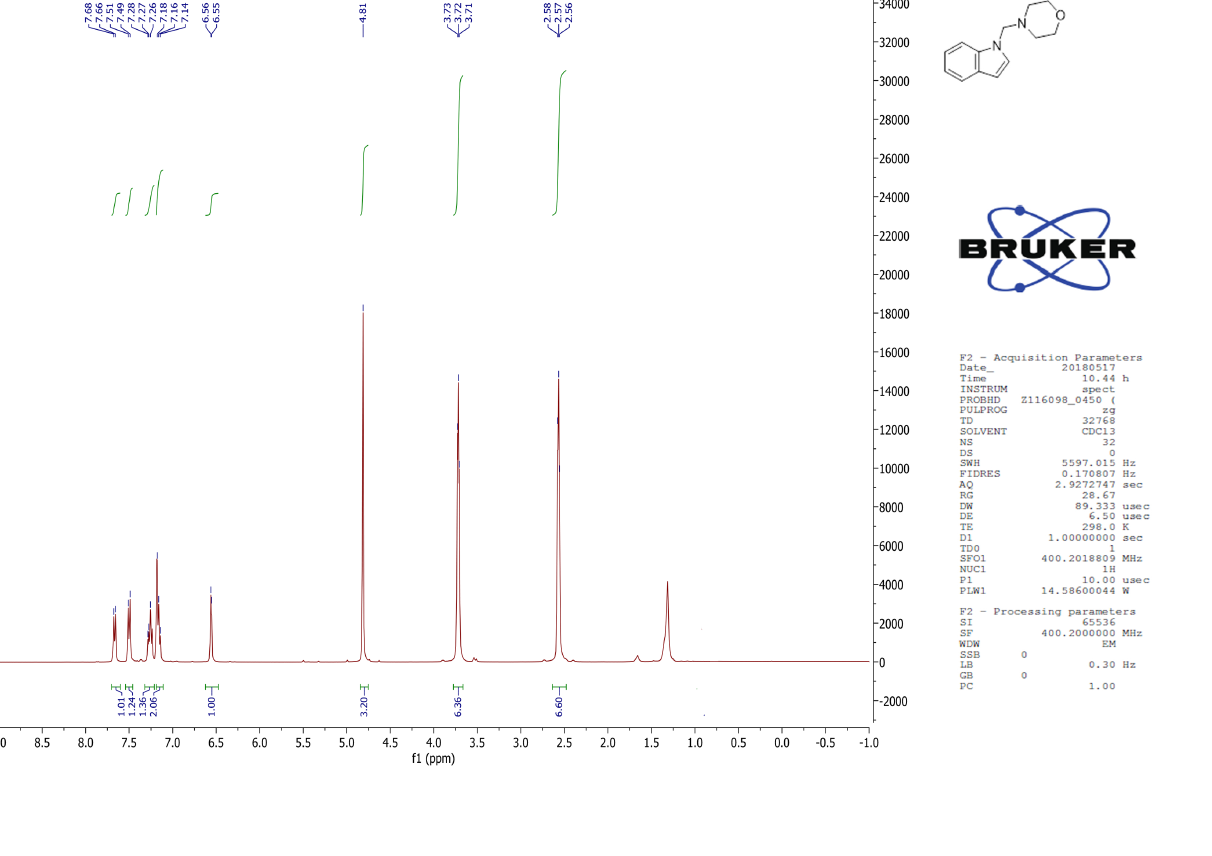


**Figure S11.** ^1^H spectra of **7**


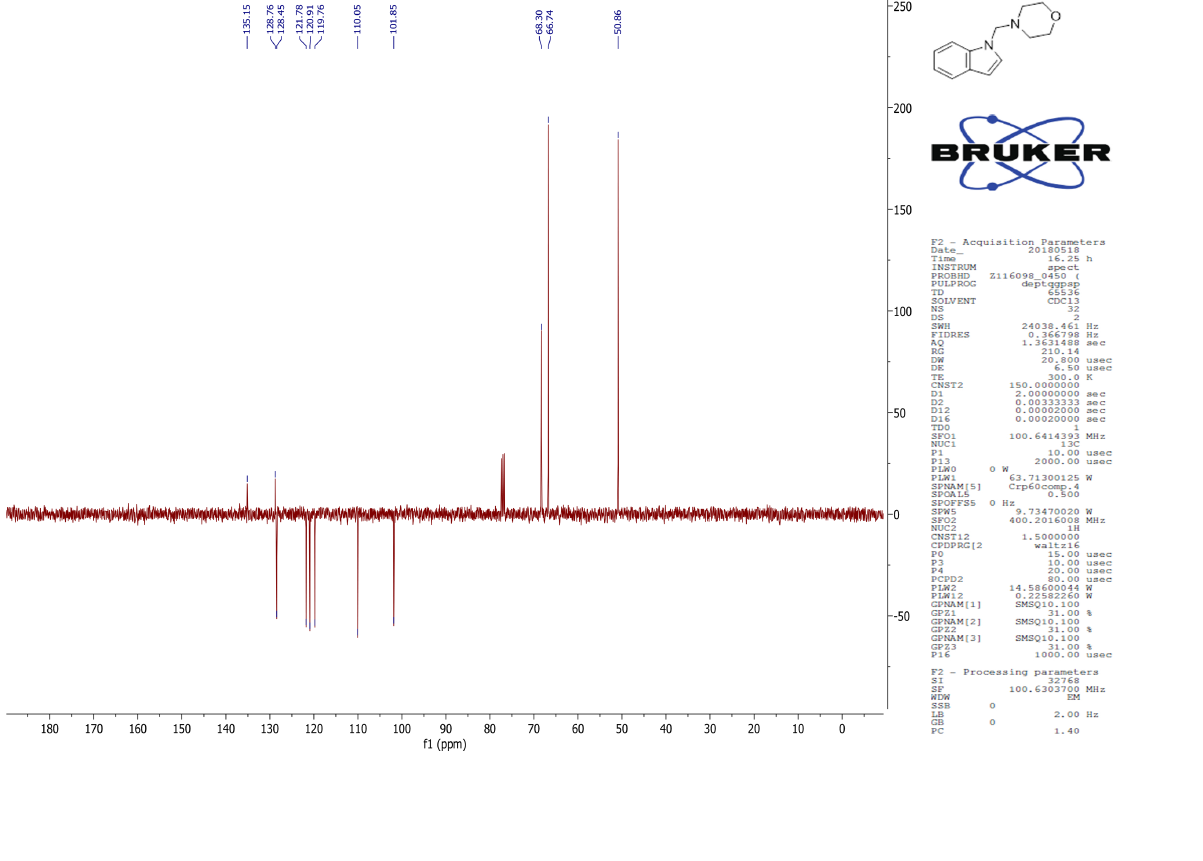


**Figure S12.** qDEPT spectra of **7**


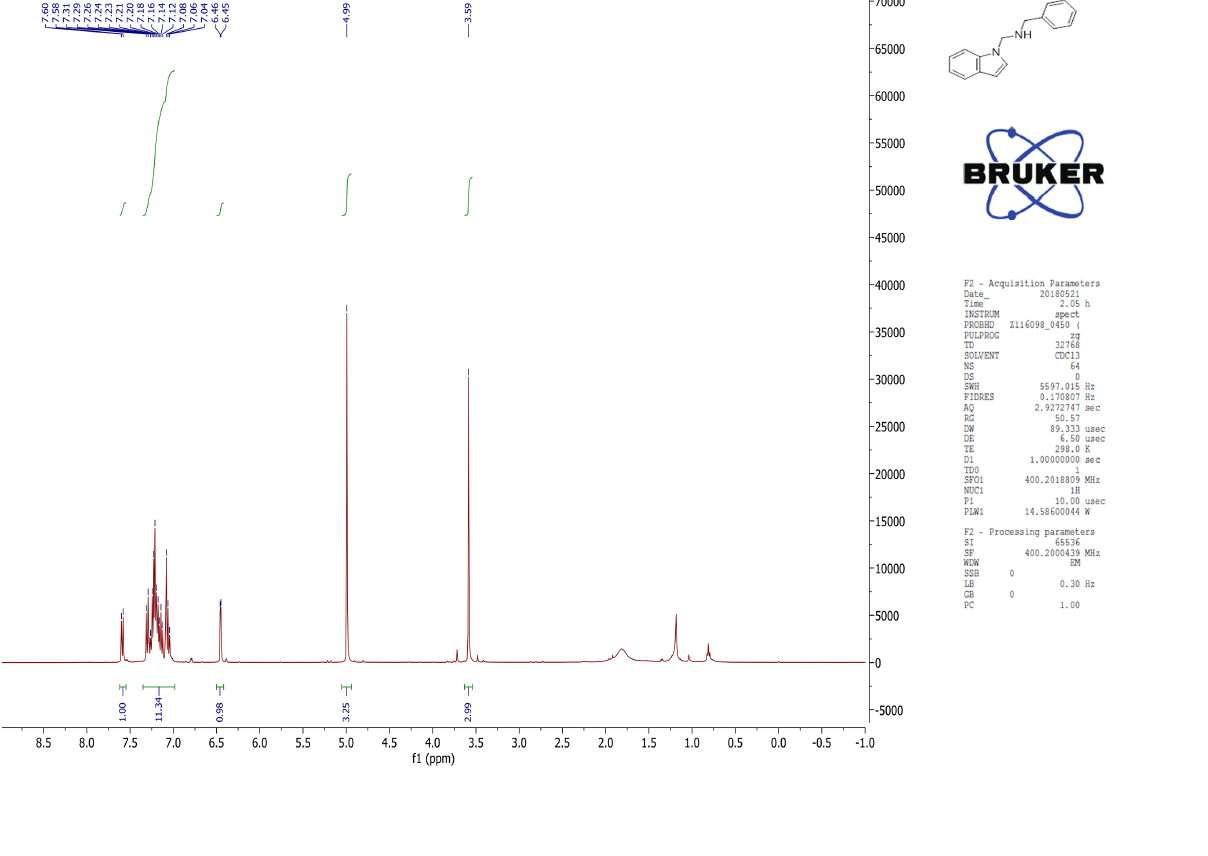


**Figure S13.** ^1^H spectra of **8**


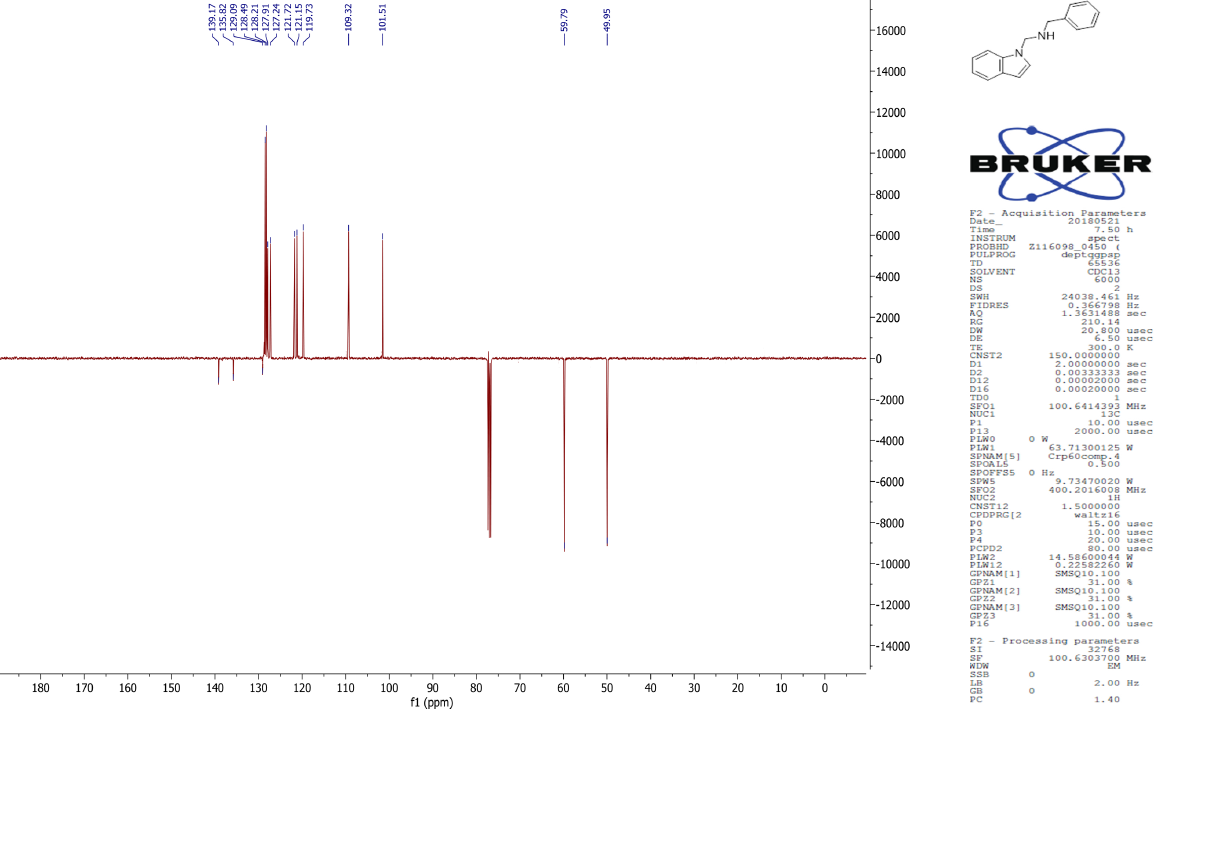


**Figure S14.** qDEPT spectra of **8**


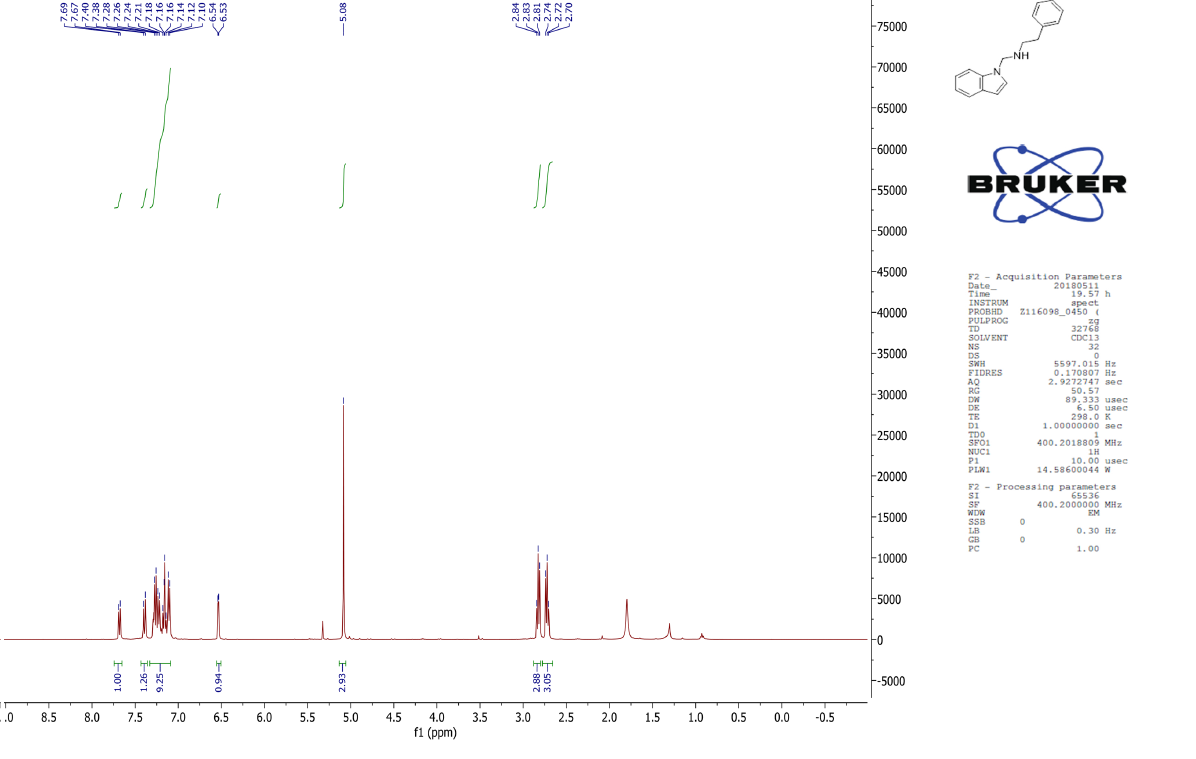


**Figure S15.** ^1^H spectra of **9**


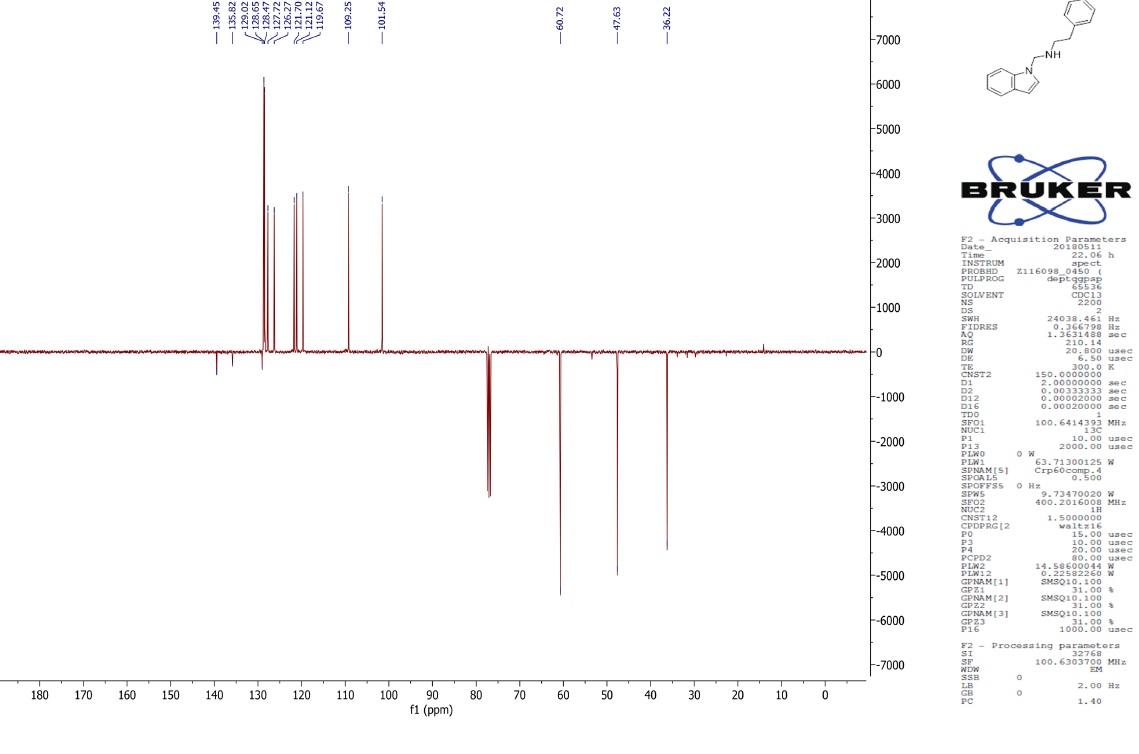


**Figure S16.** qDEPT spectra of **9**


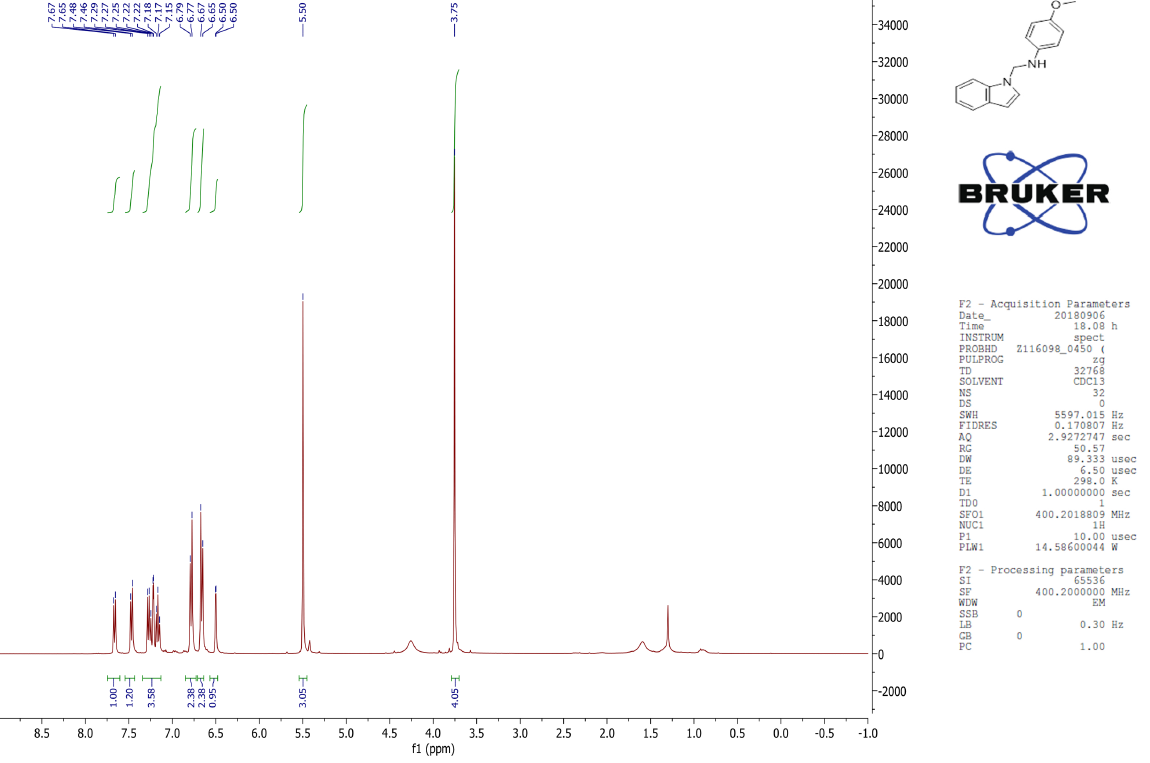


**Figure S17.** ^1^H spectra of **10**


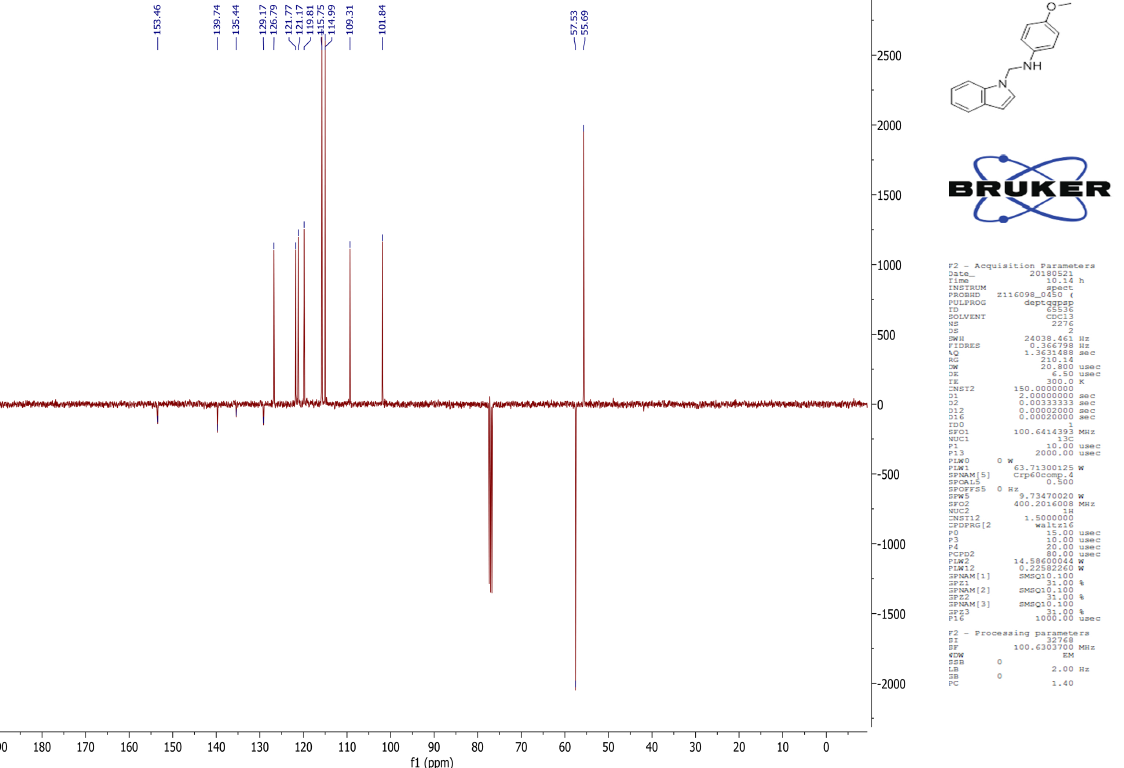


**Figure S18.** qDEPT spectra of **10**


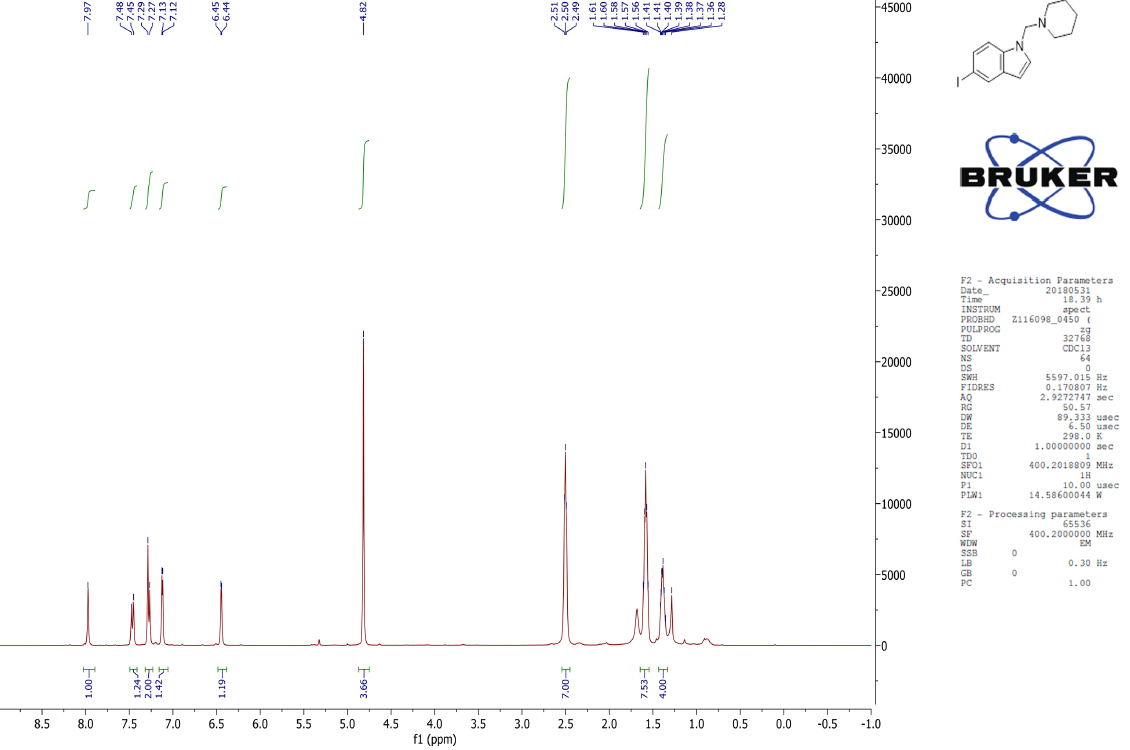


**Figure S19.** ^1^H spectra of **11**


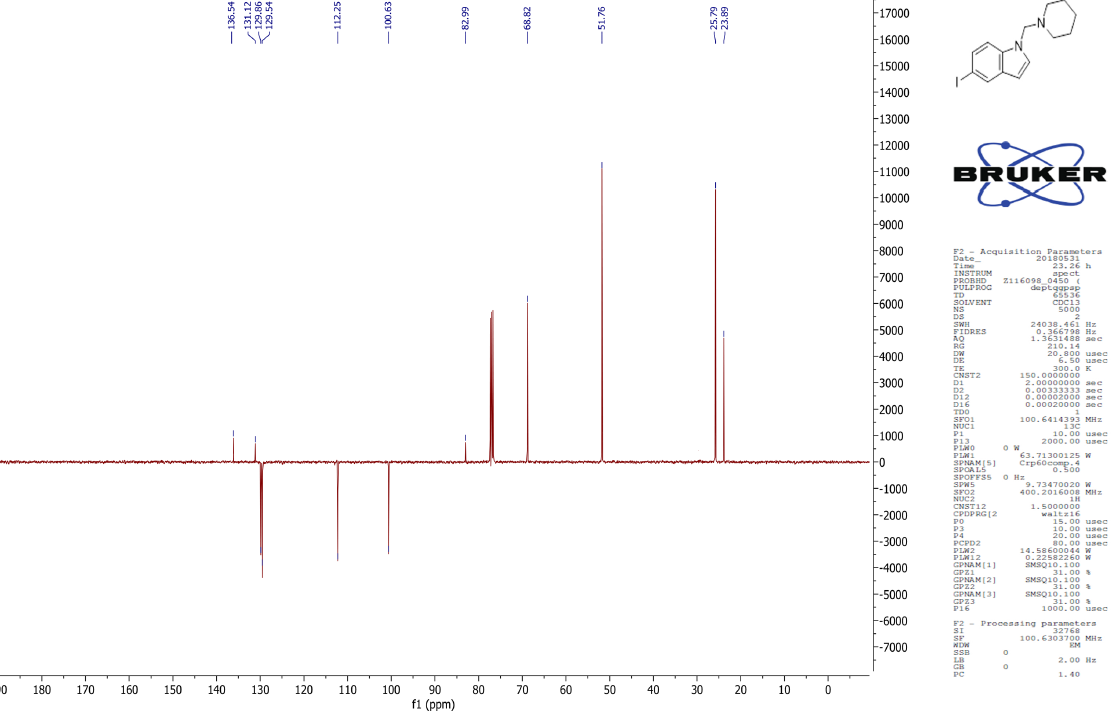


**Figure S20.** qDEPT spectra of **11**


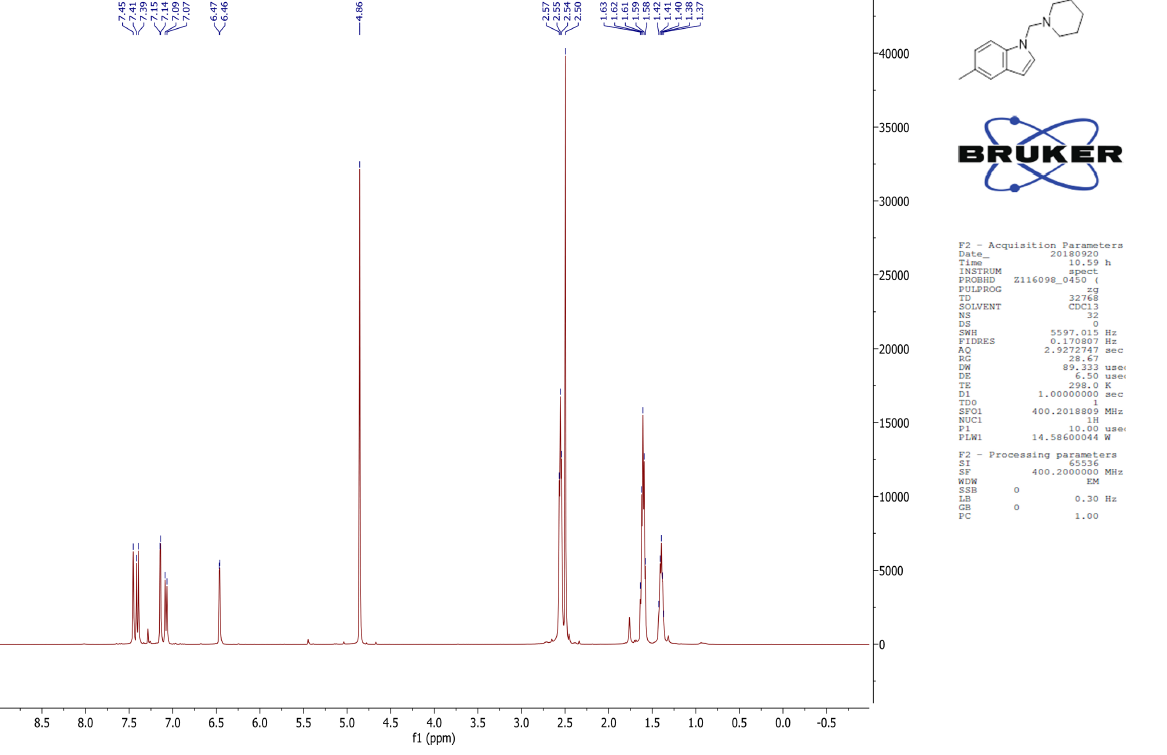


**Figure S21.** ^1^H spectra of **12**


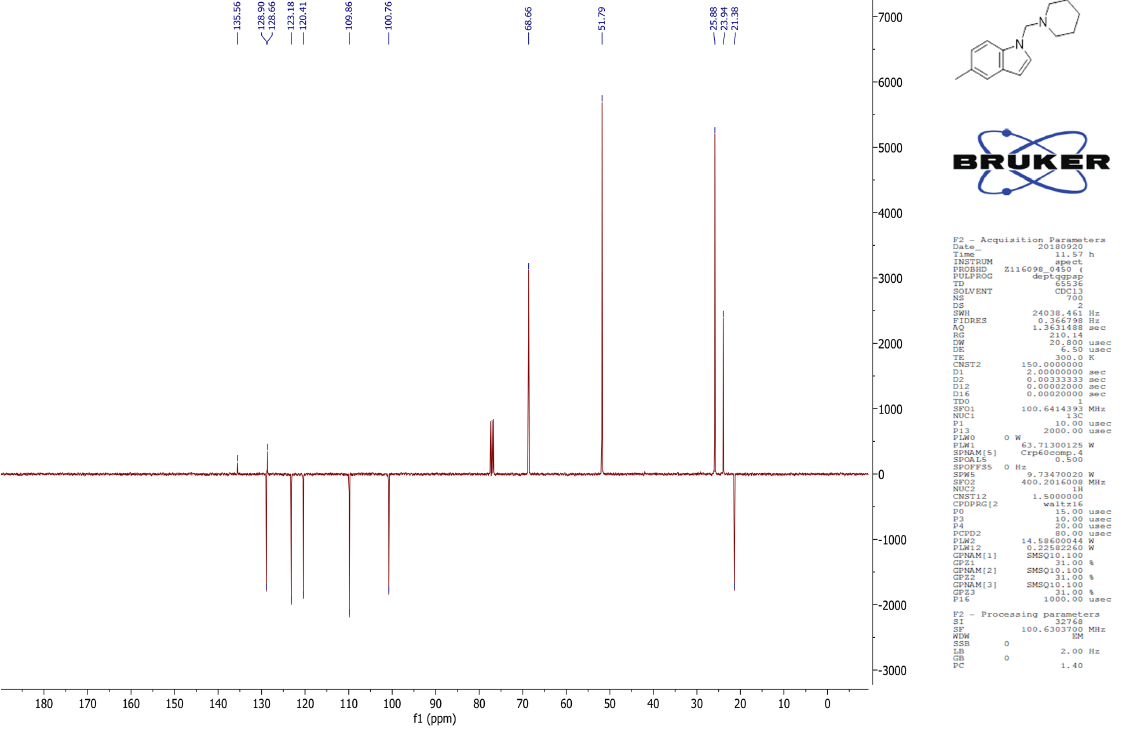


**Figure S22.** qDEPT spectra of **12**


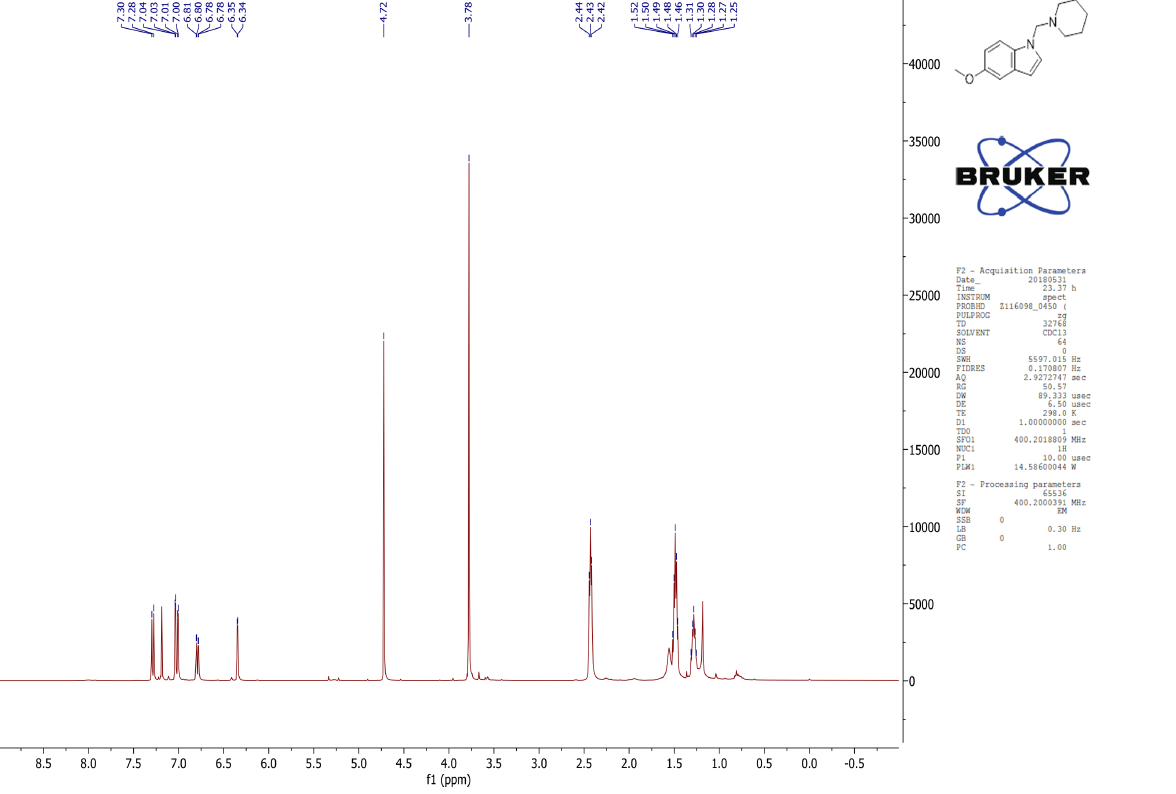


**Figure S23.** ^1^H spectra of **13**


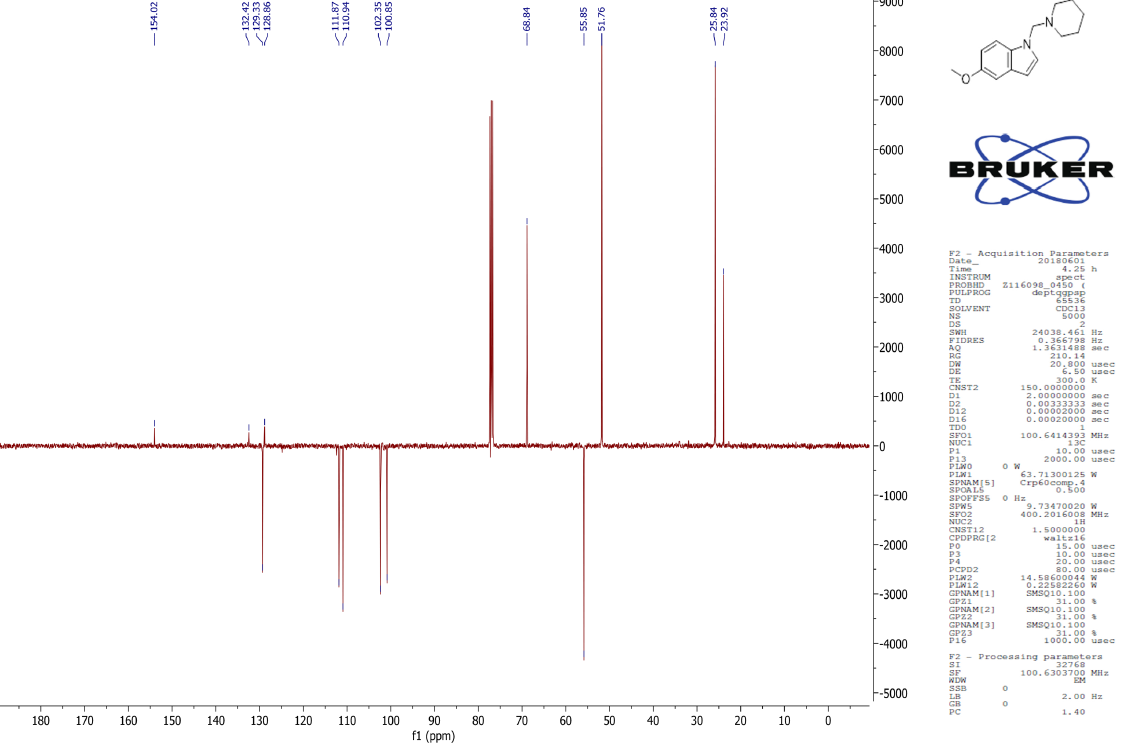


**Figure S24.** qDEPT spectra of **13**


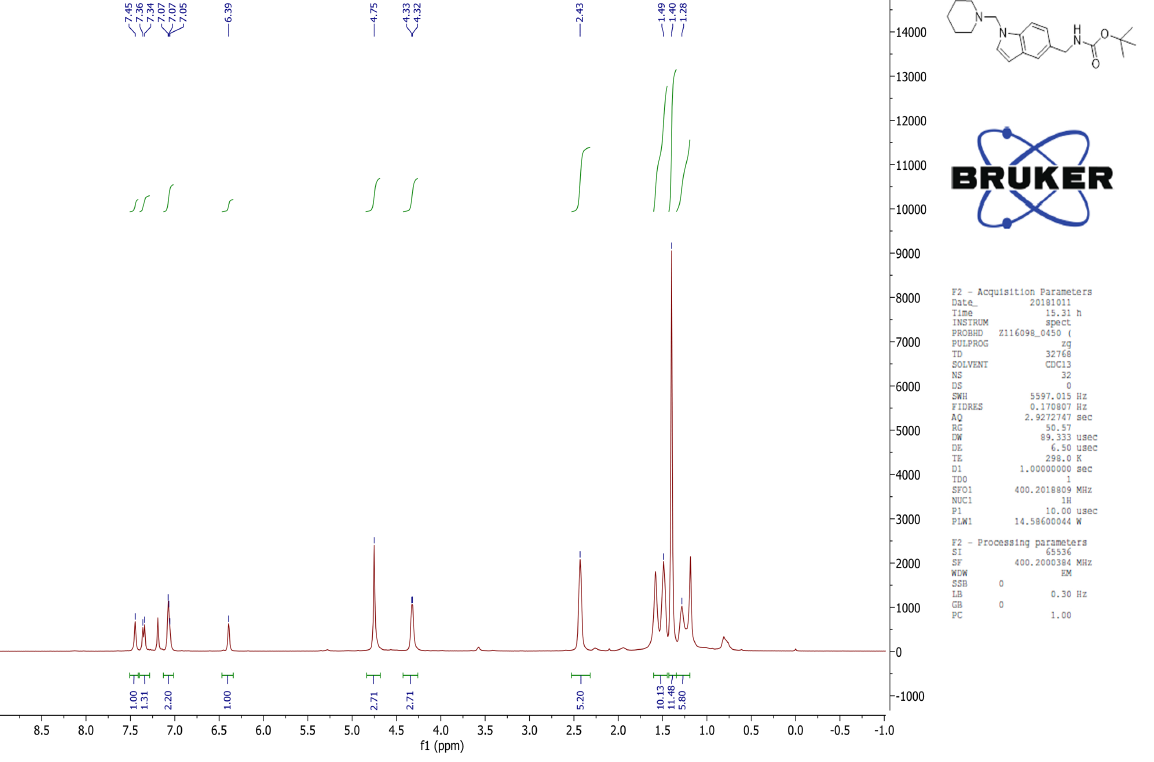


**Figure S25.** ^1^H spectra of **14**


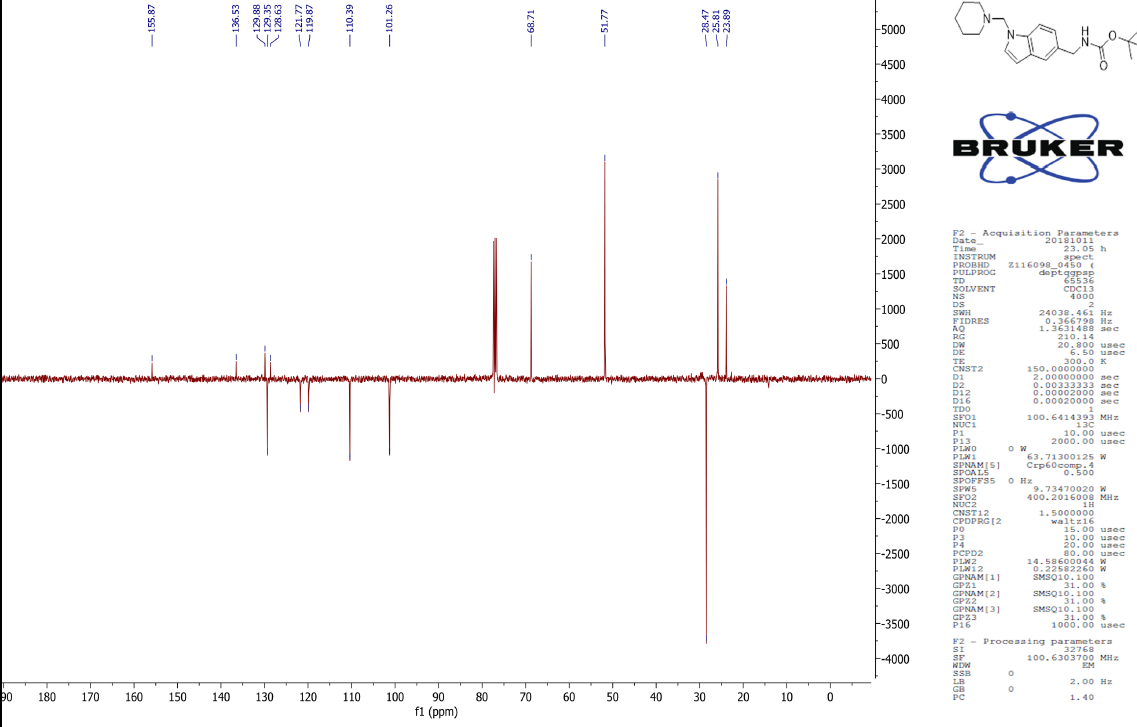


**Figure S26.** qDEPT spectra of **14**


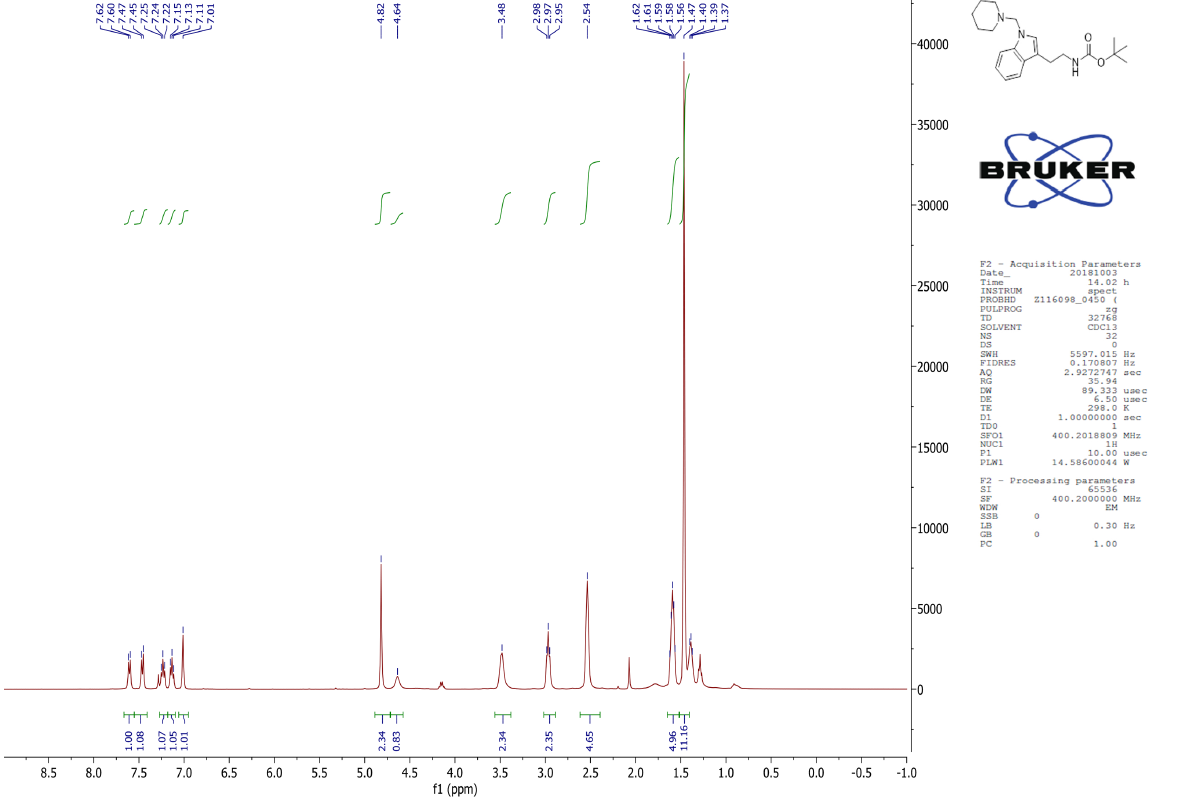


**Figure S27.** ^1^H spectra of **15**


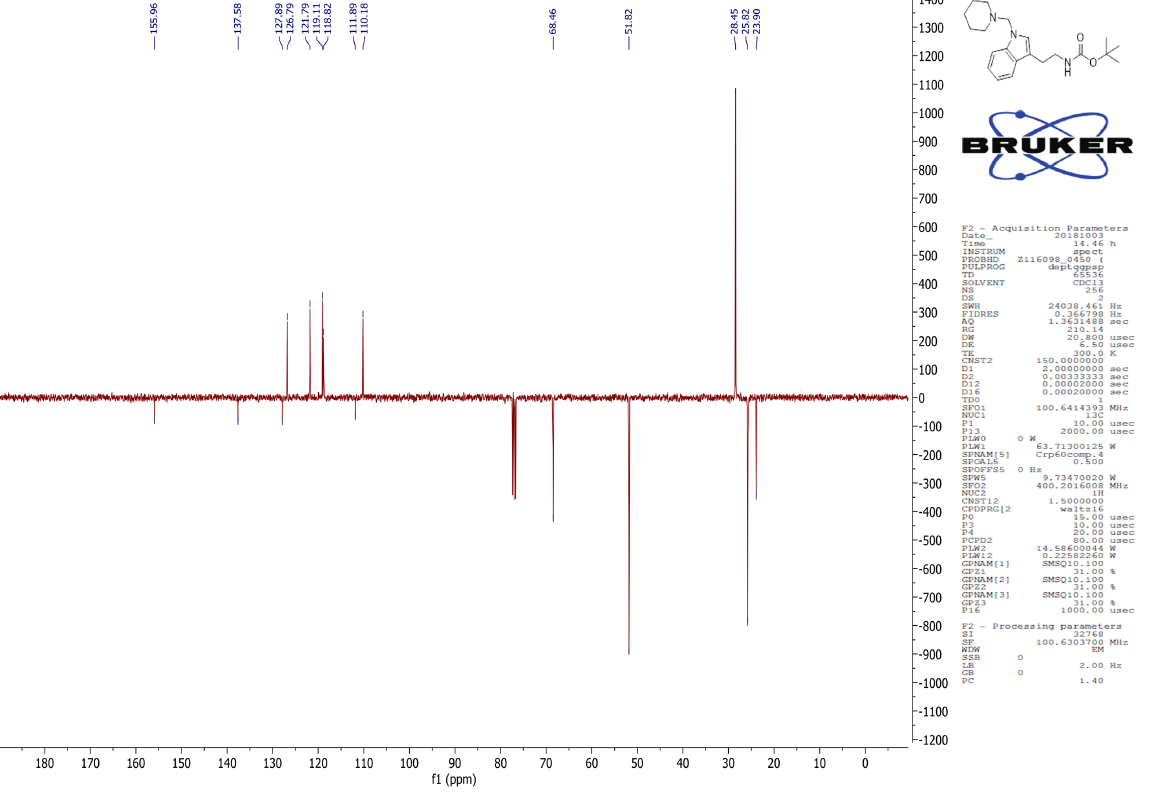


**Figure S28.** qDEPT spectra of **15**


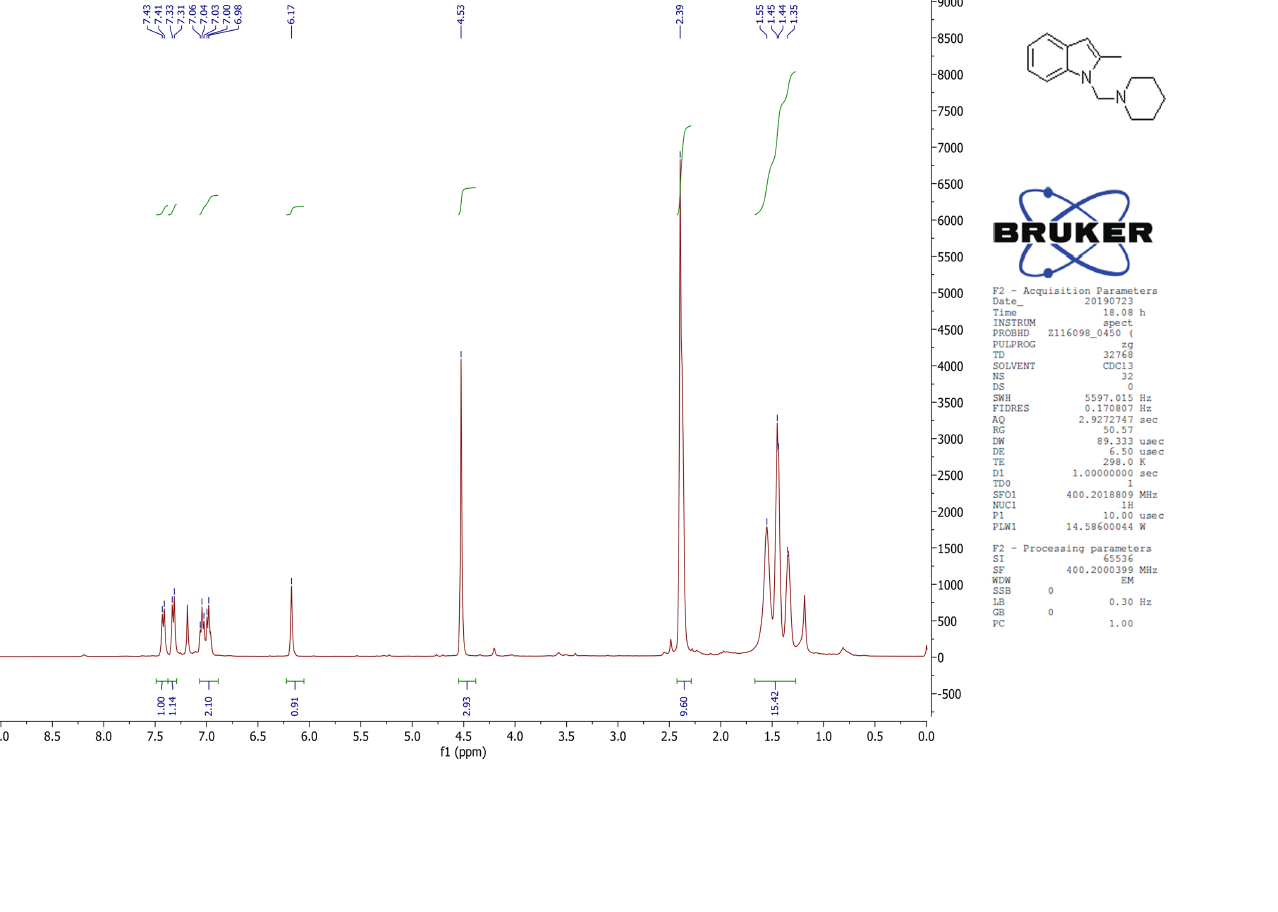


**Figure S29.** ^1^H spectra of **16**


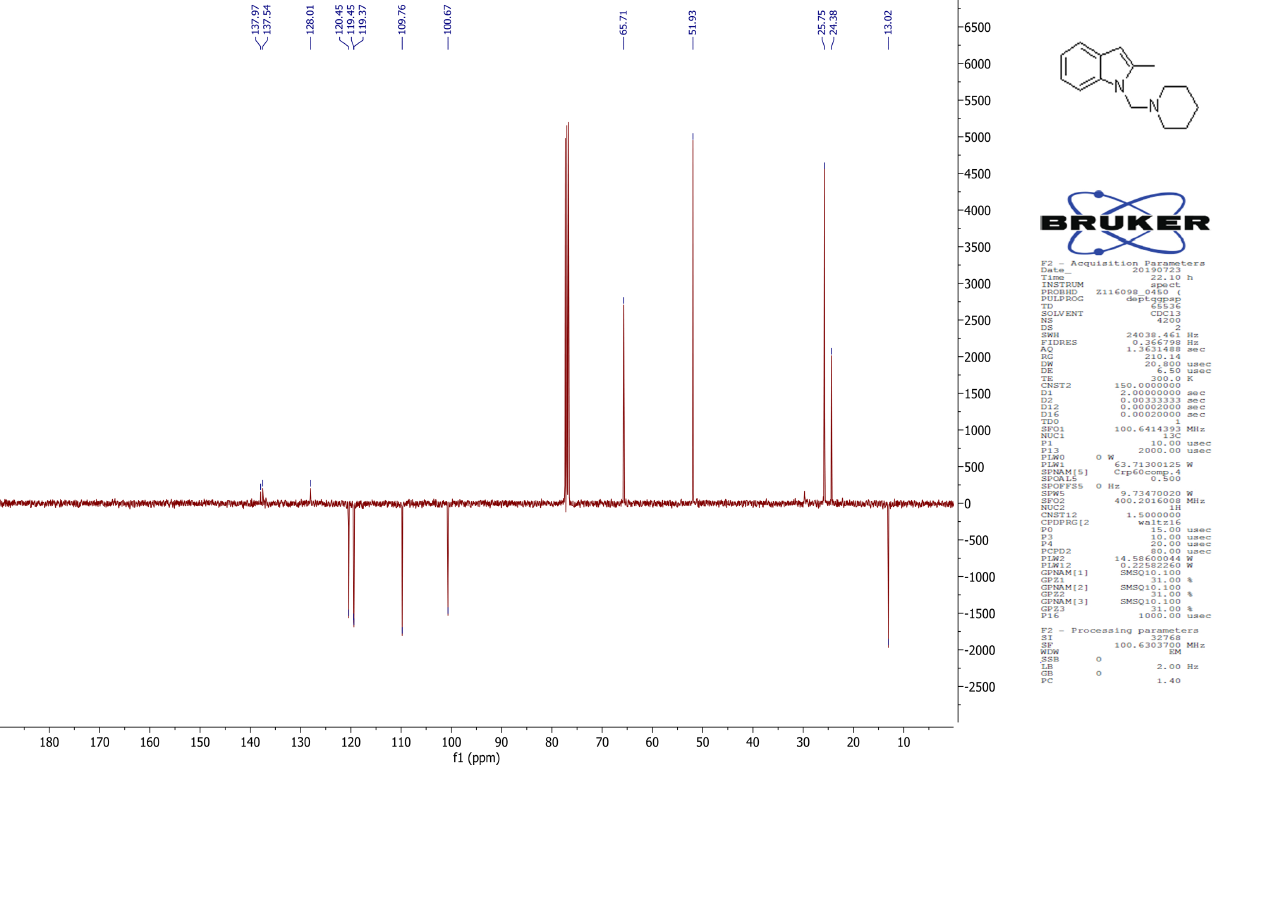


**Figure S30.** qDEPT spectra of **16**


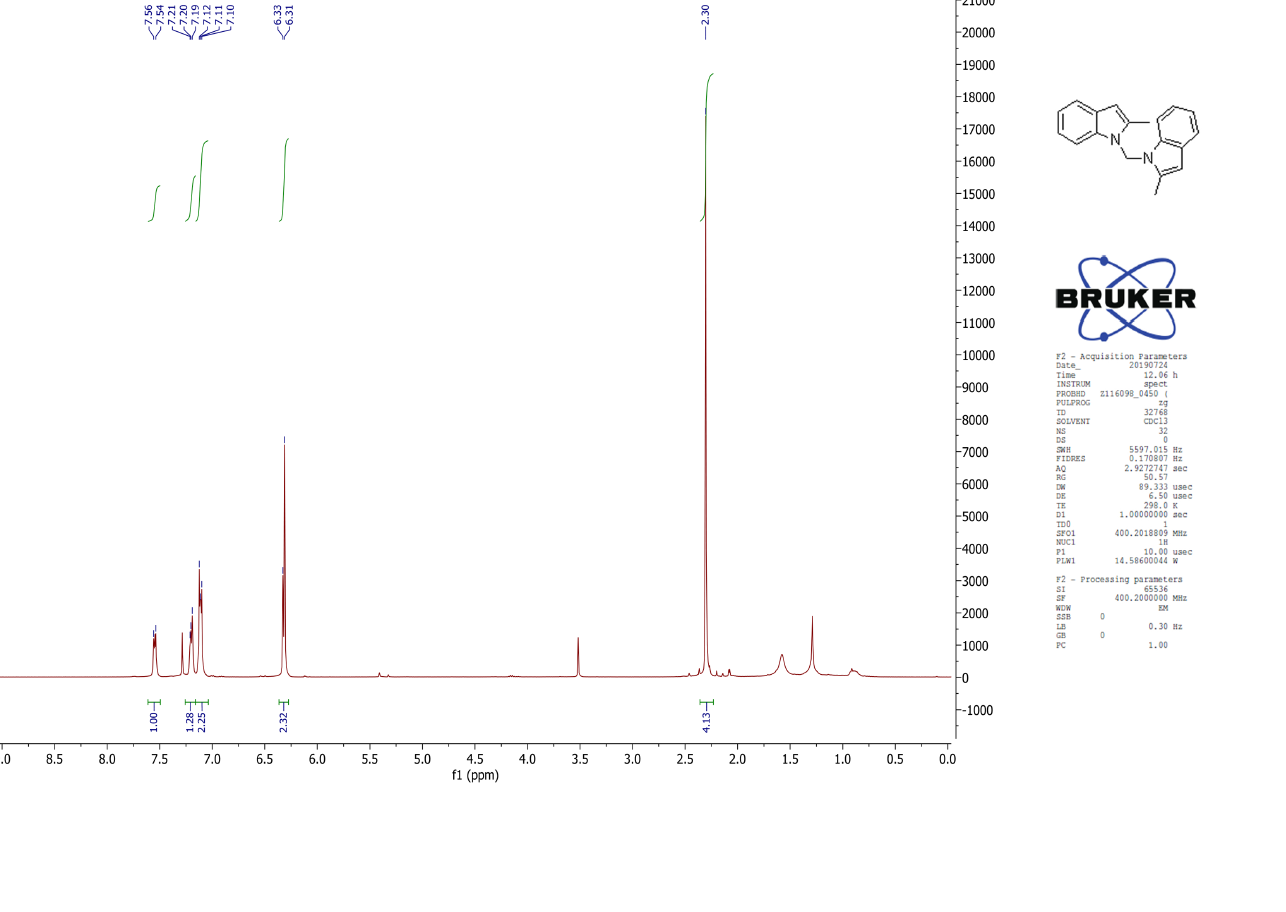


**Figure S31.** ^1^H spectra of **17**


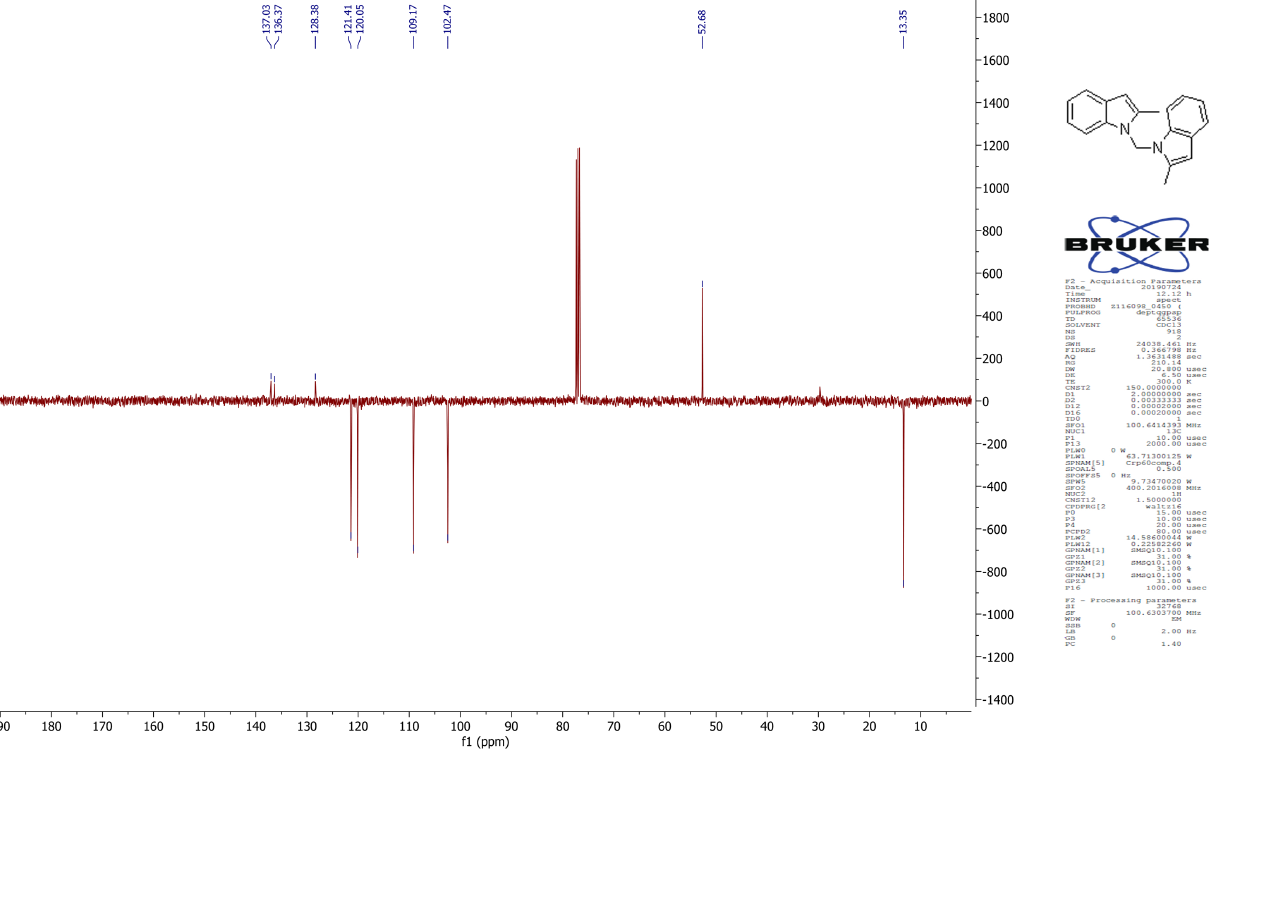


**Figure S32.** qDEPT spectra of **17**


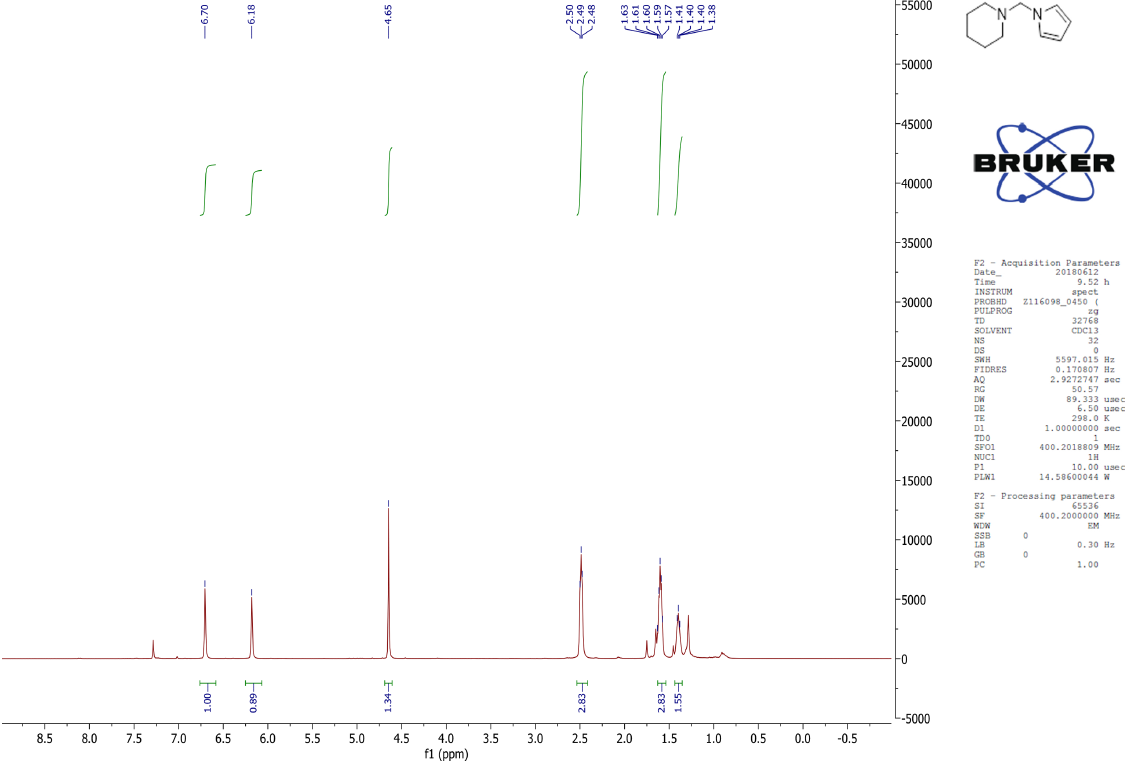


**Figure S33.** ^1^H spectra of **18**


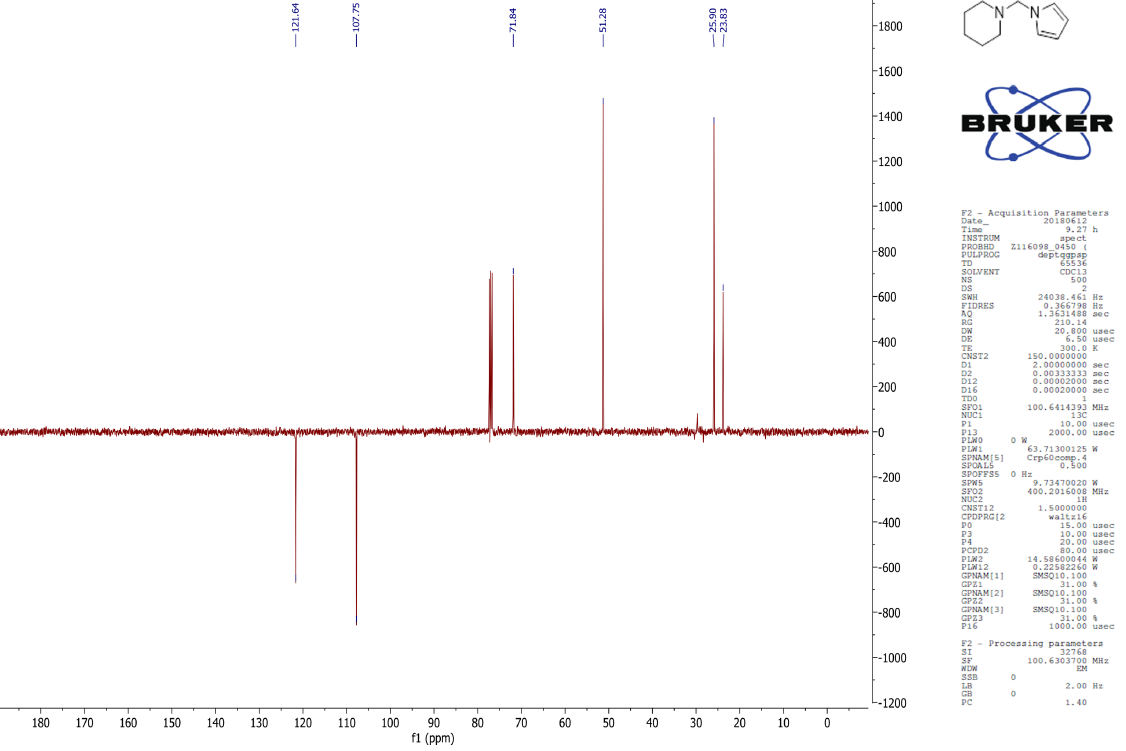


**Figure S34.** qDEPT spectra of **18**


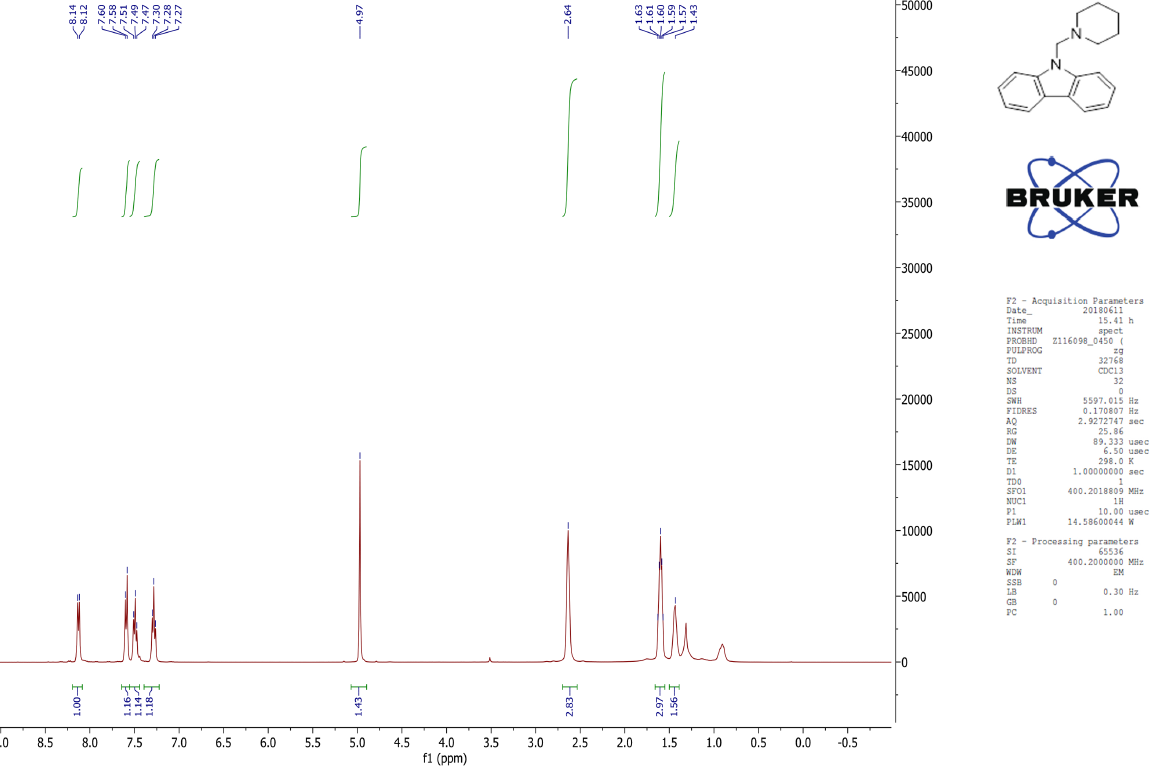


**Figure S35.** ^1^H spectra of **19**


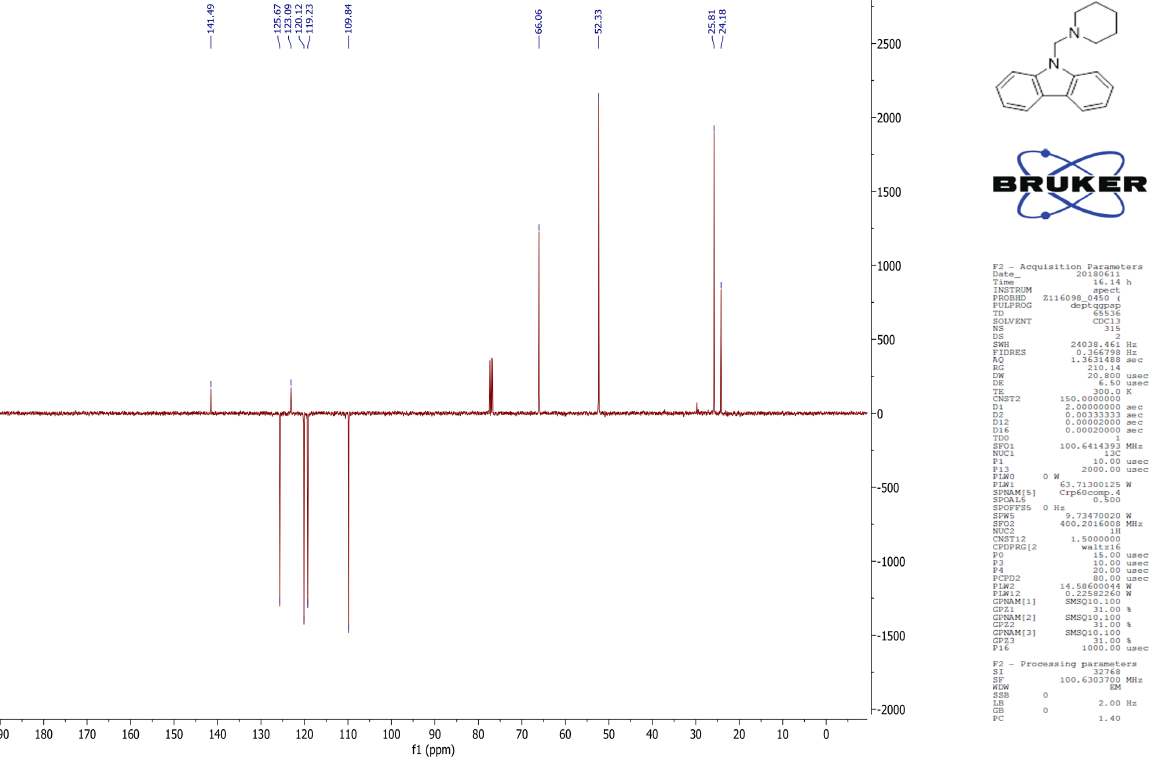


**Figure S36.** qDEPT spectra of **19**


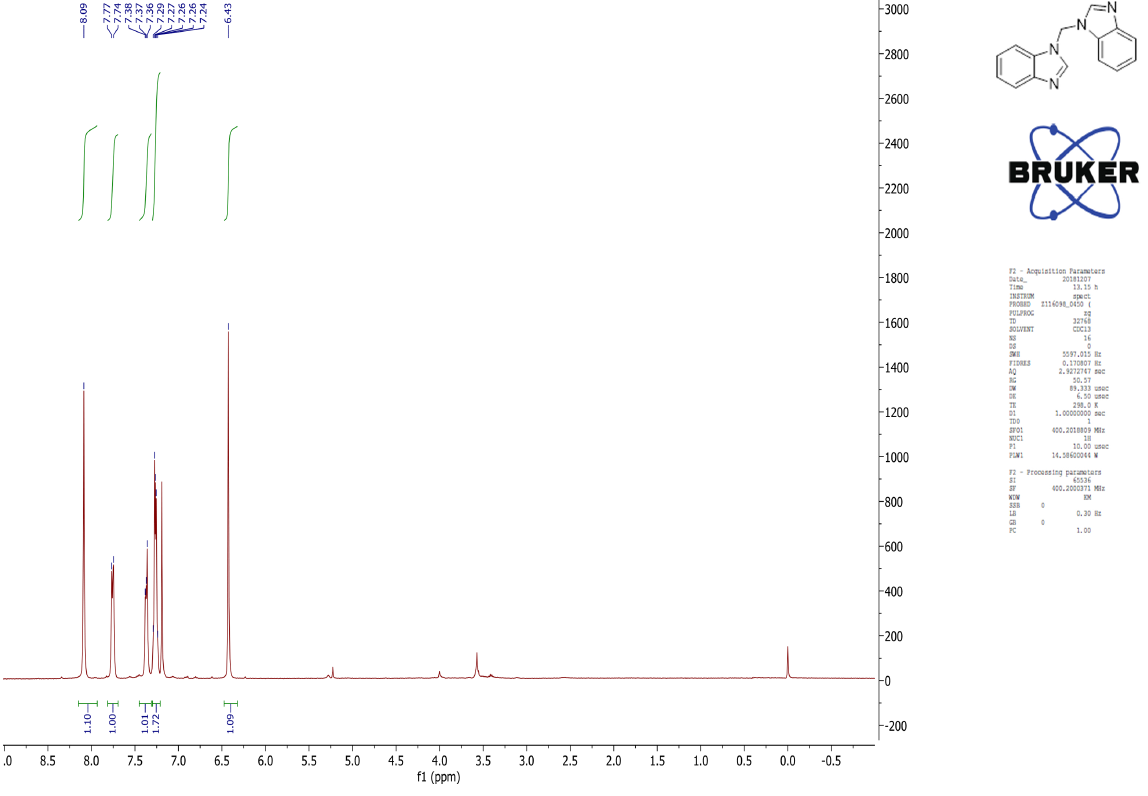


**Figure S37.** ^1^H spectra of **20**


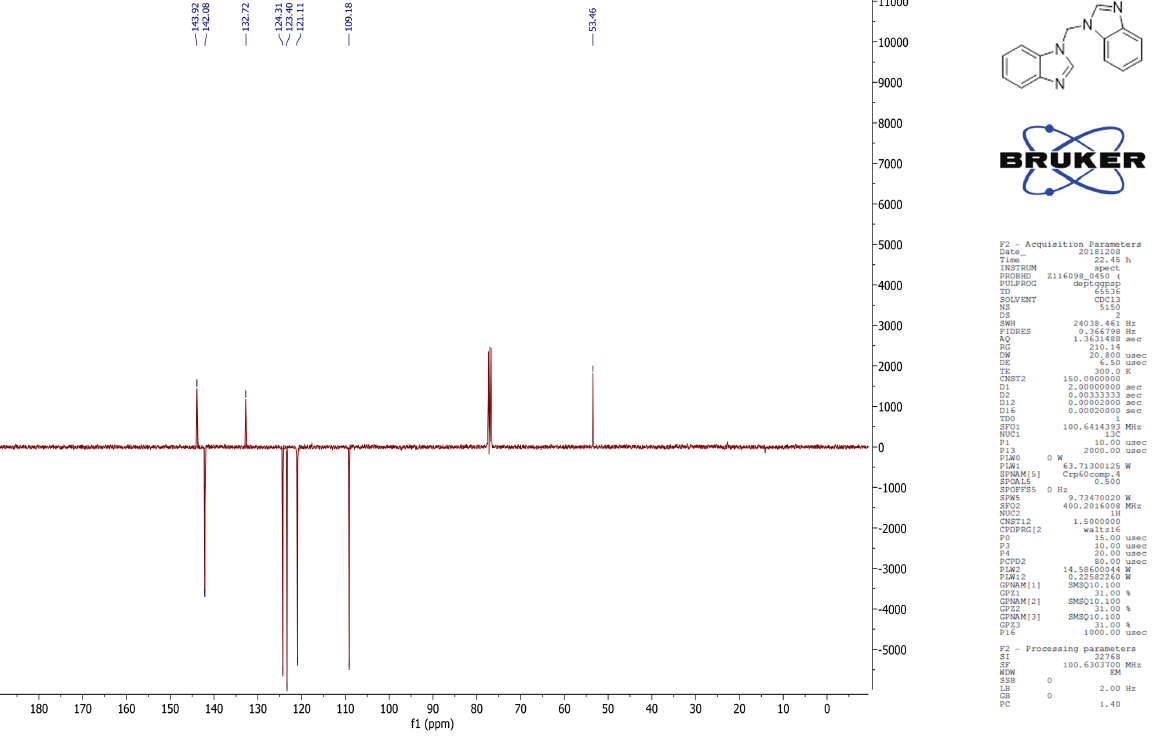


**Figure S38.** qDEPT spectra of **20**


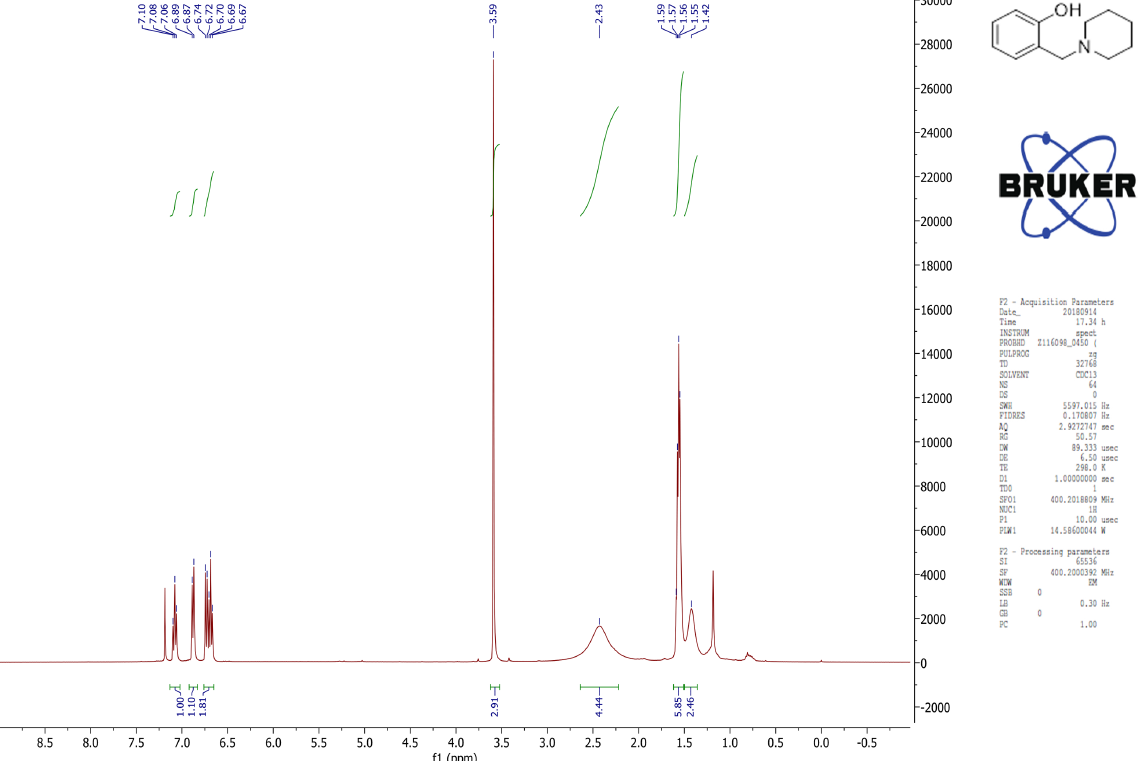


**Figure S39.** ^1^H spectra of **21**


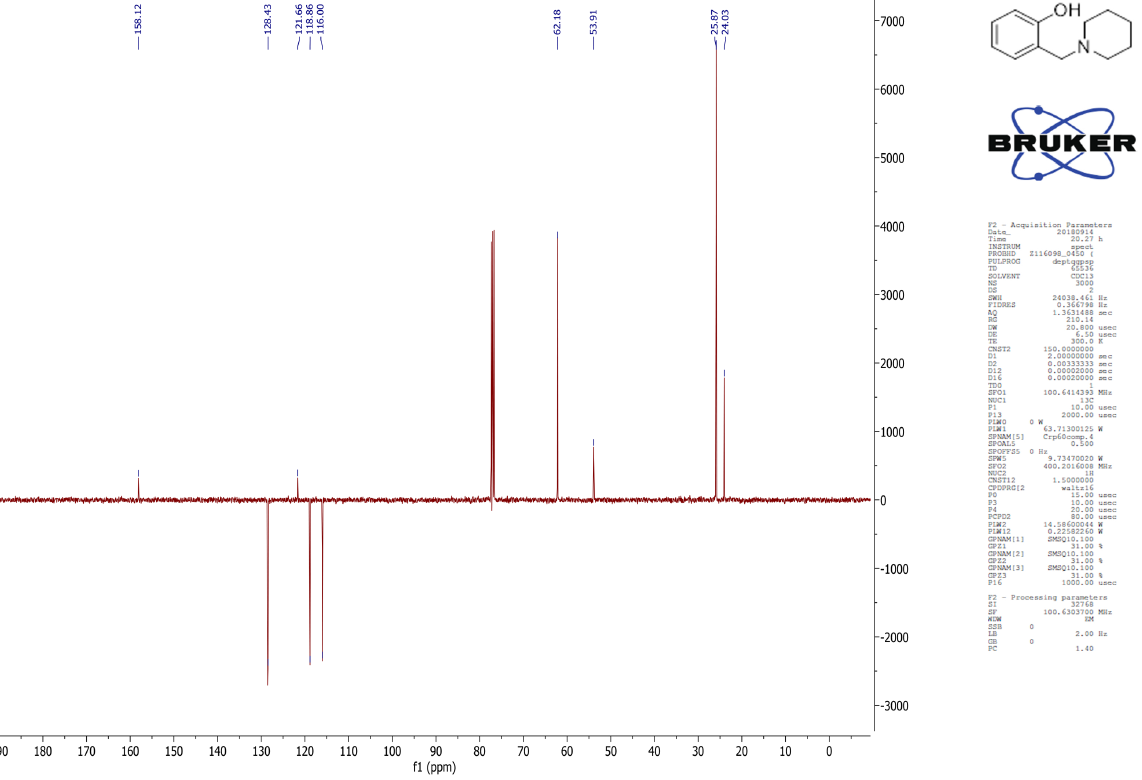


**Figure S40.** qDEPT spectra of **21**


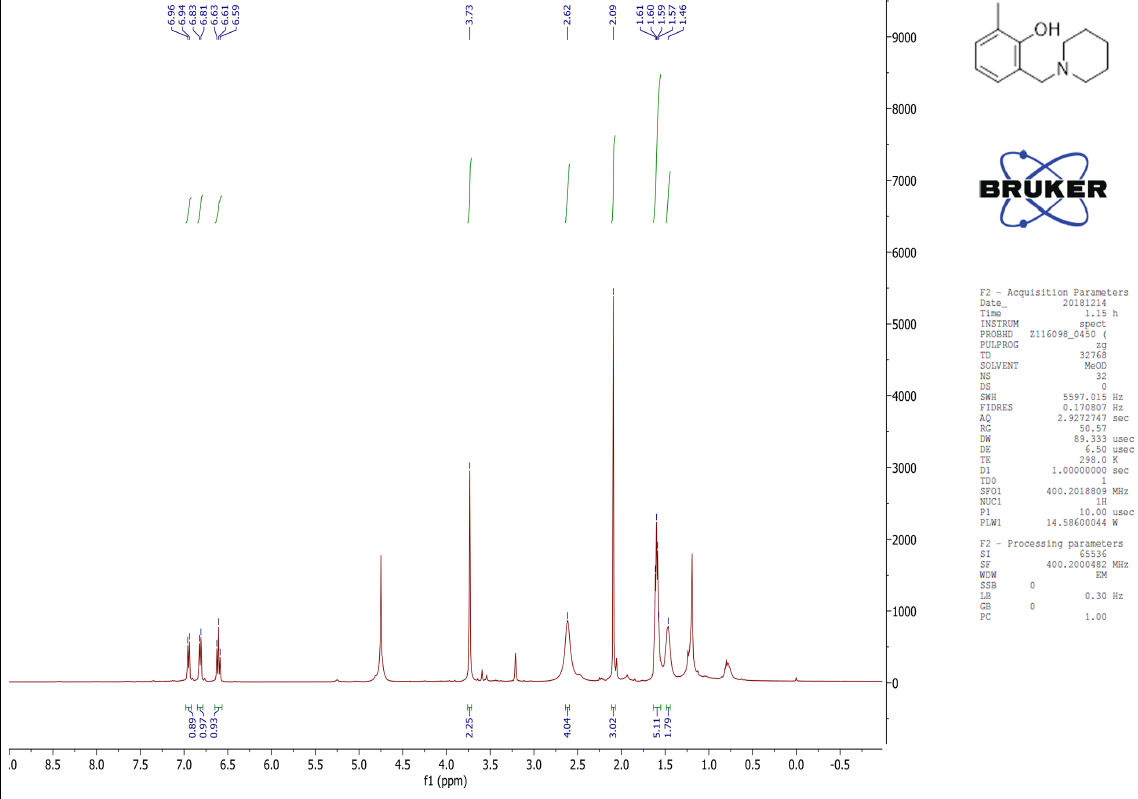


**Figure S41.** ^1^H spectra of **22**


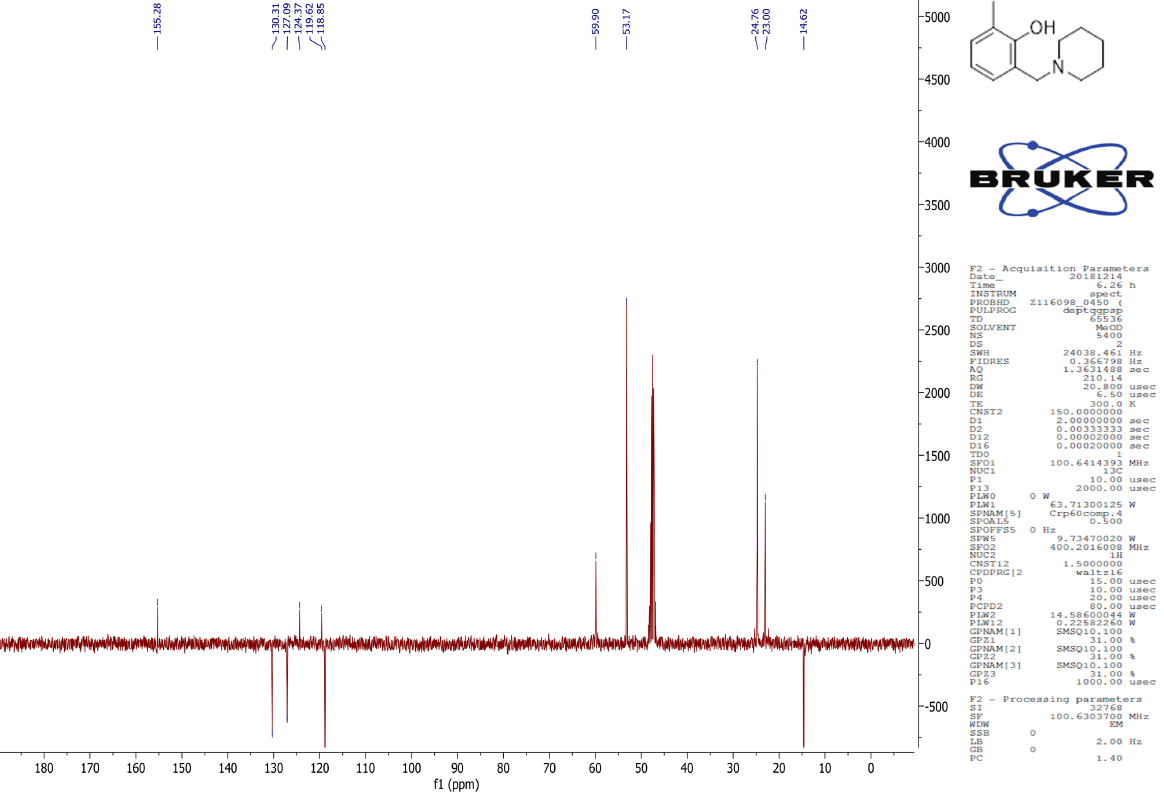


**Figure S42.** qDEPT spectra of **22**


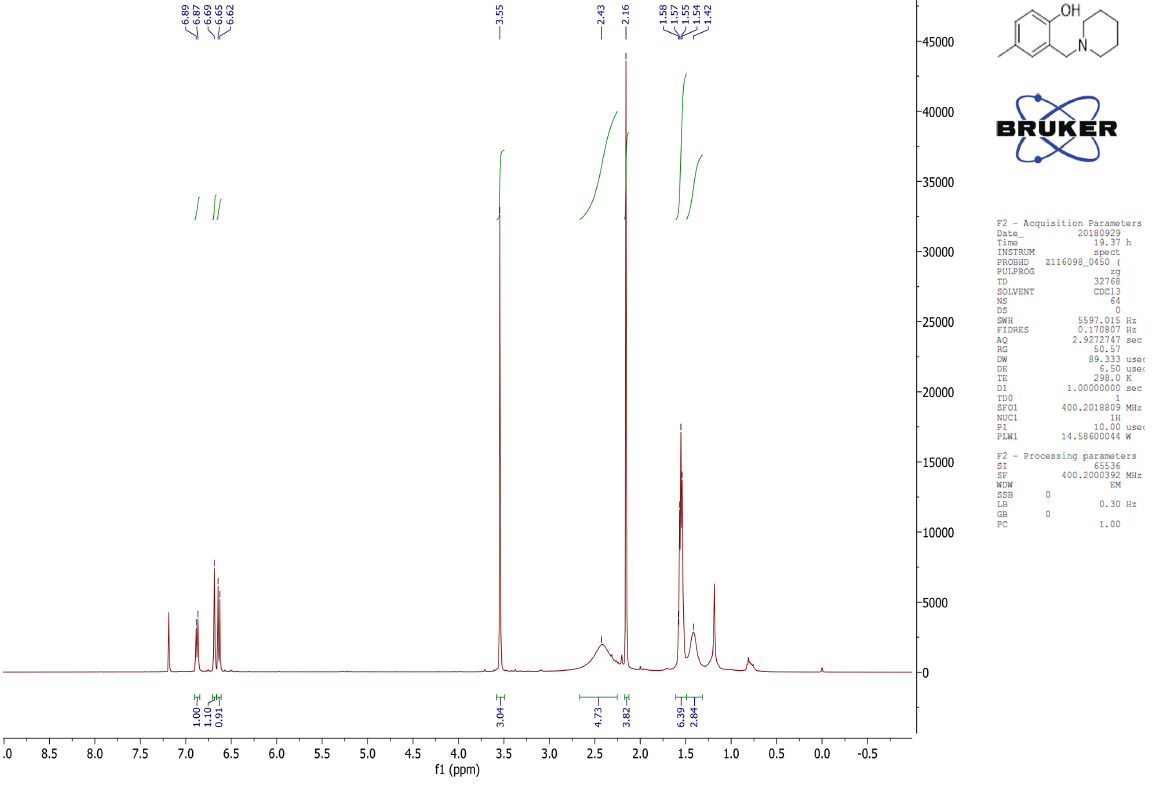


**Figure S43.** ^1^H spectra of **23**


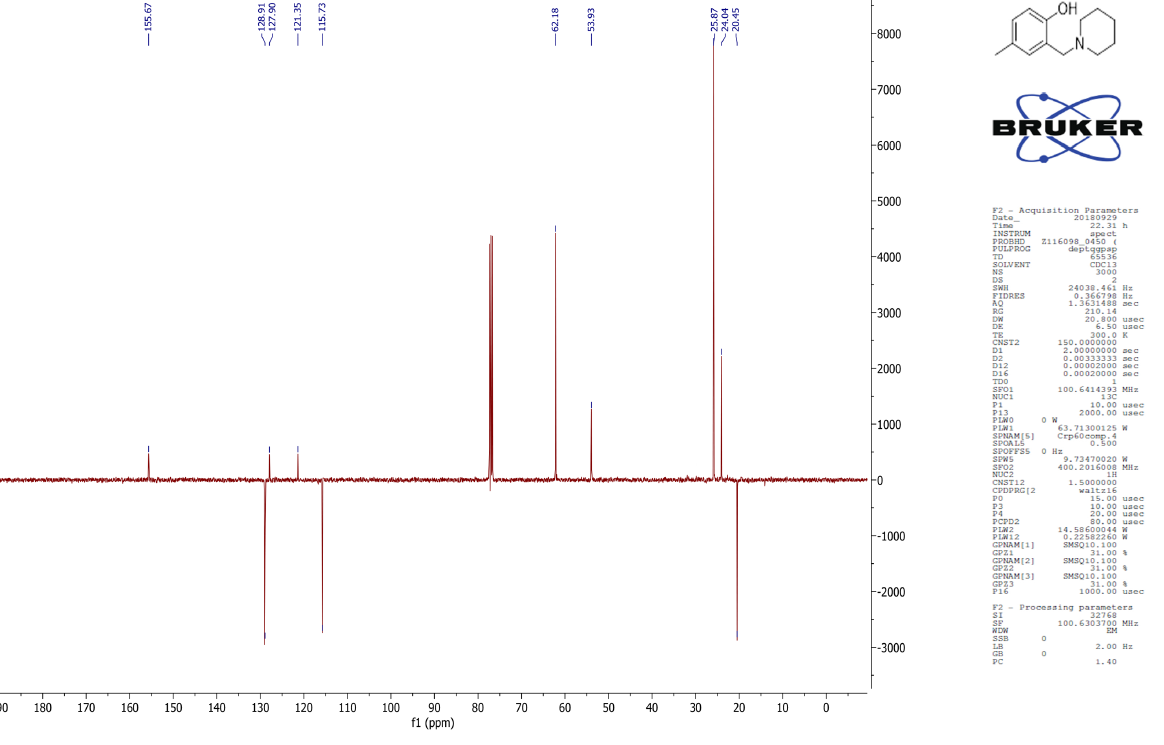


**Figure S44.** qDEPT spectra of **23**


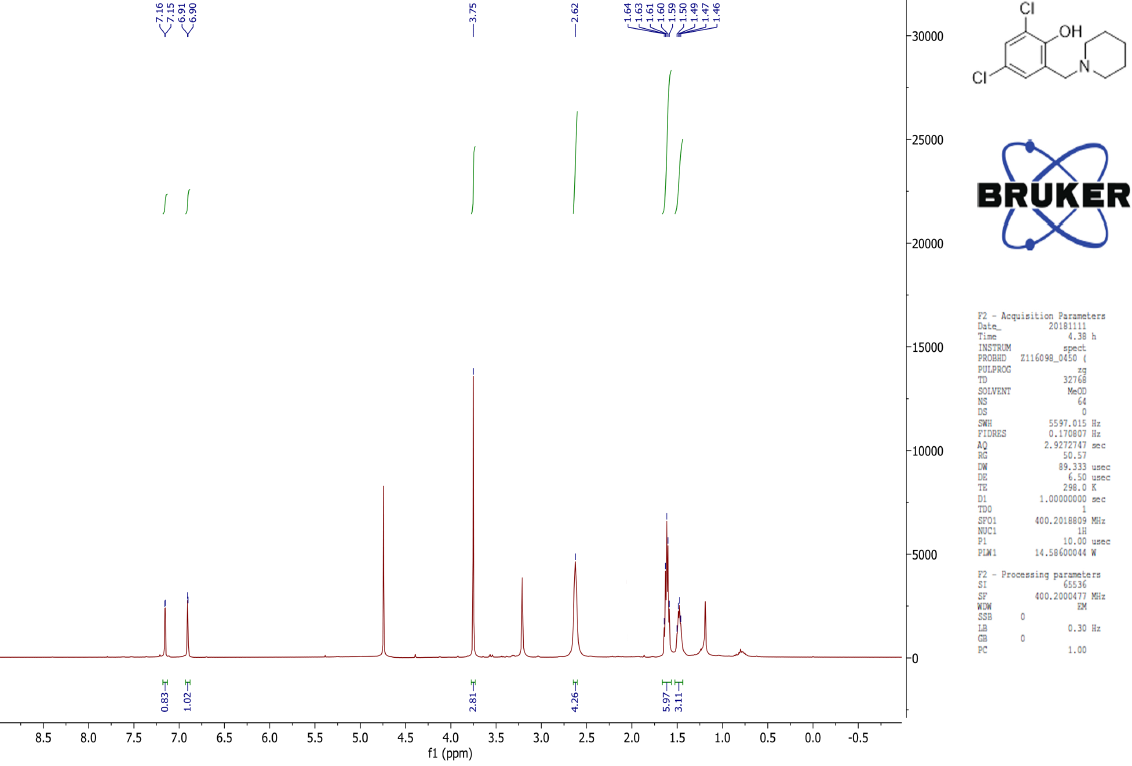


**Figure S45.** ^1^H spectra of **24**


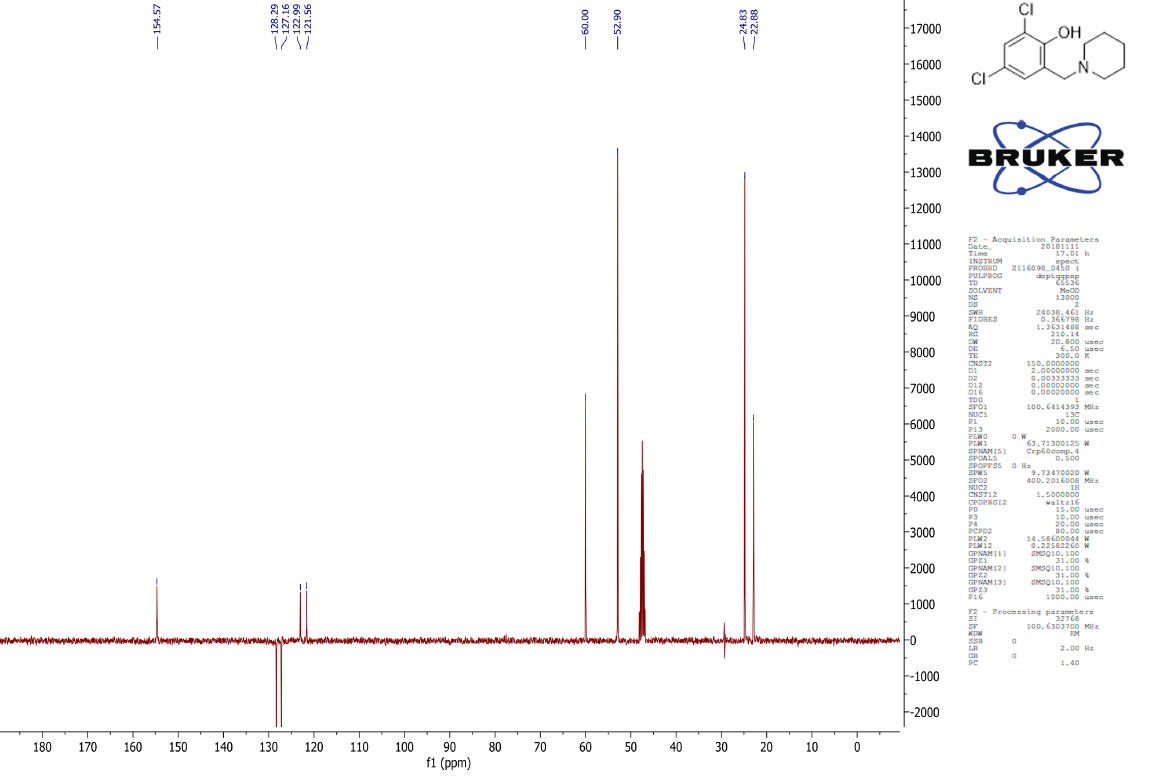


**Figure S46.** qDEPT spectra of **24**


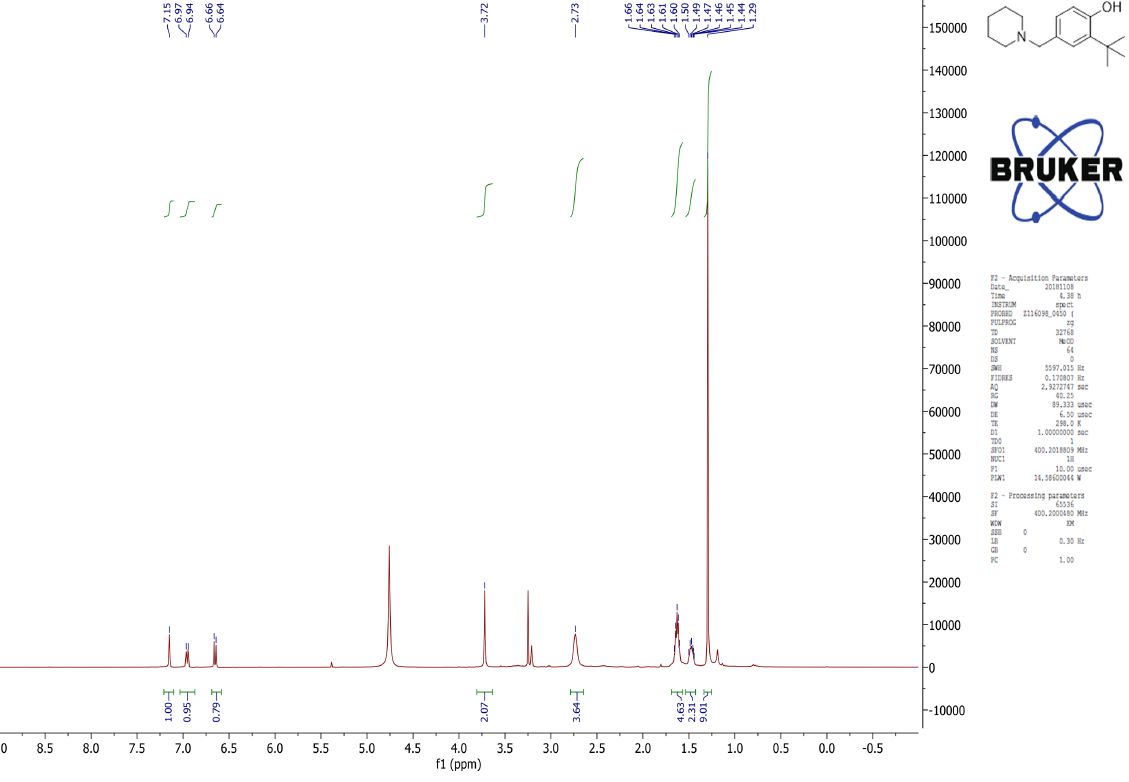


**Figure S47.** ^1^H spectra of **25**


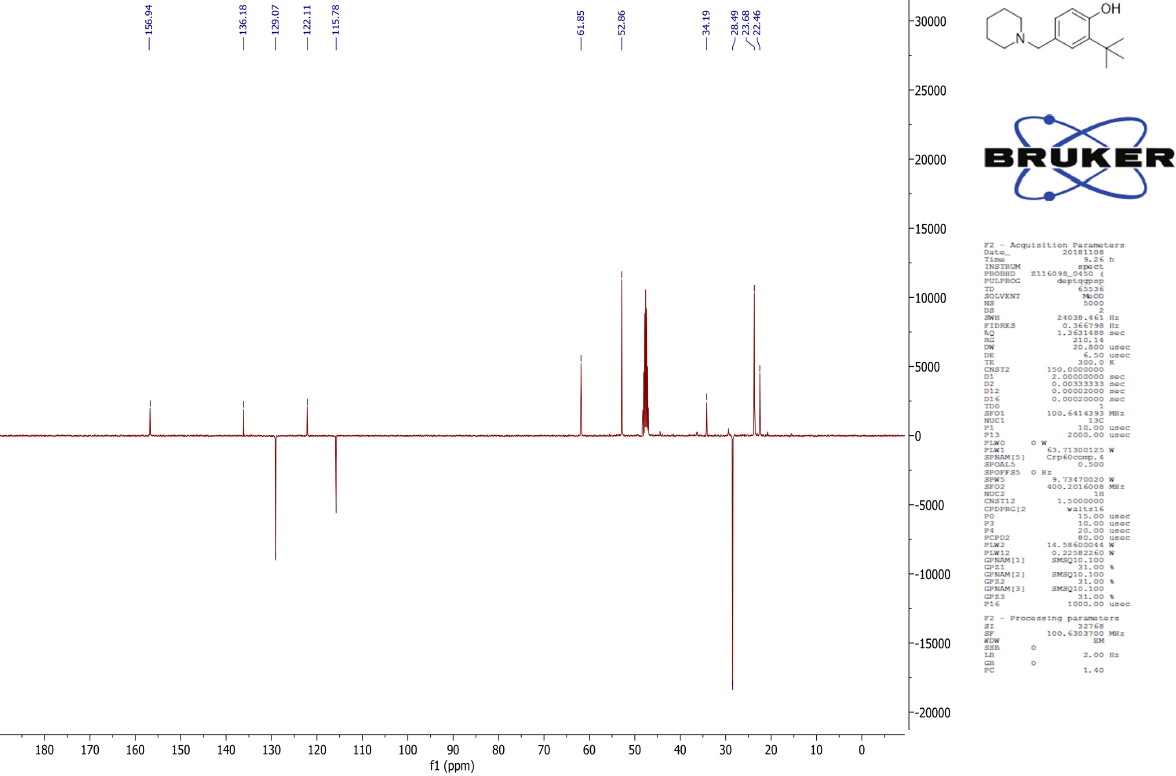


**Figure S48.** qDEPT spectra of **25**


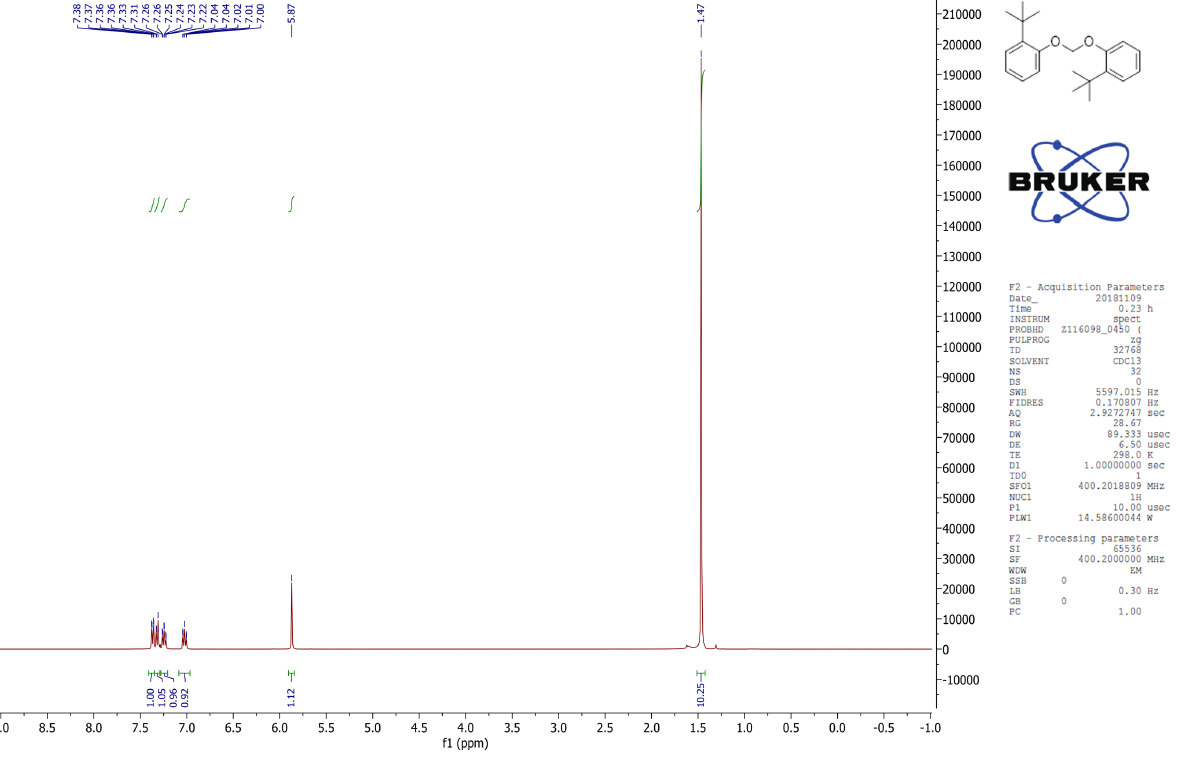


**Figure S49.** ^1^H spectra of **26**


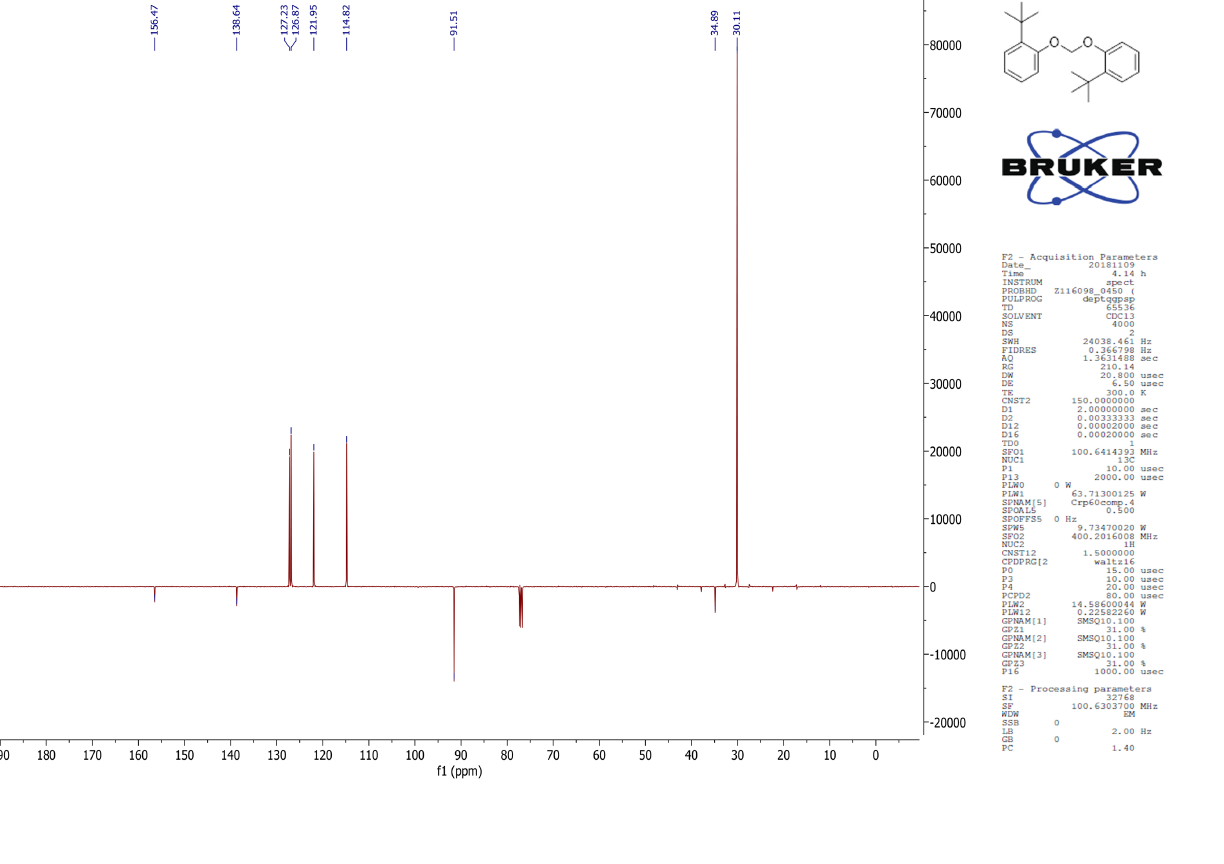


**Figure S50.** qDEPT spectra of **26**


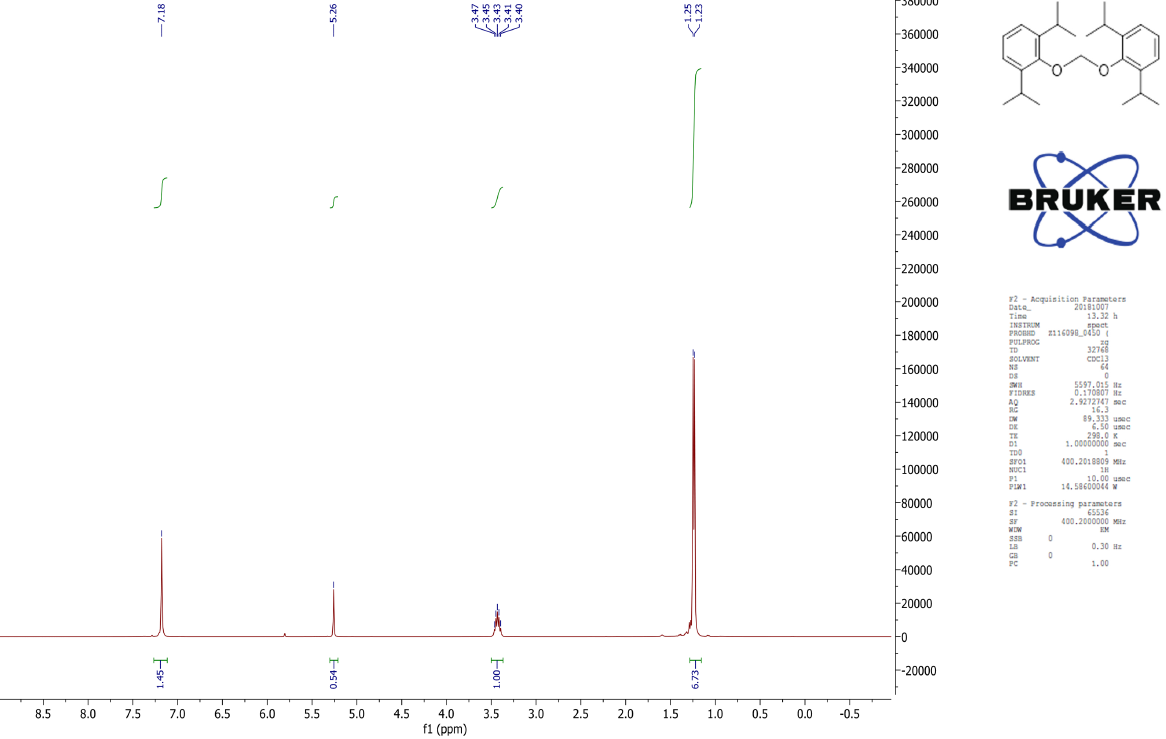


**Figure S51.** ^1^H spectra of **27**


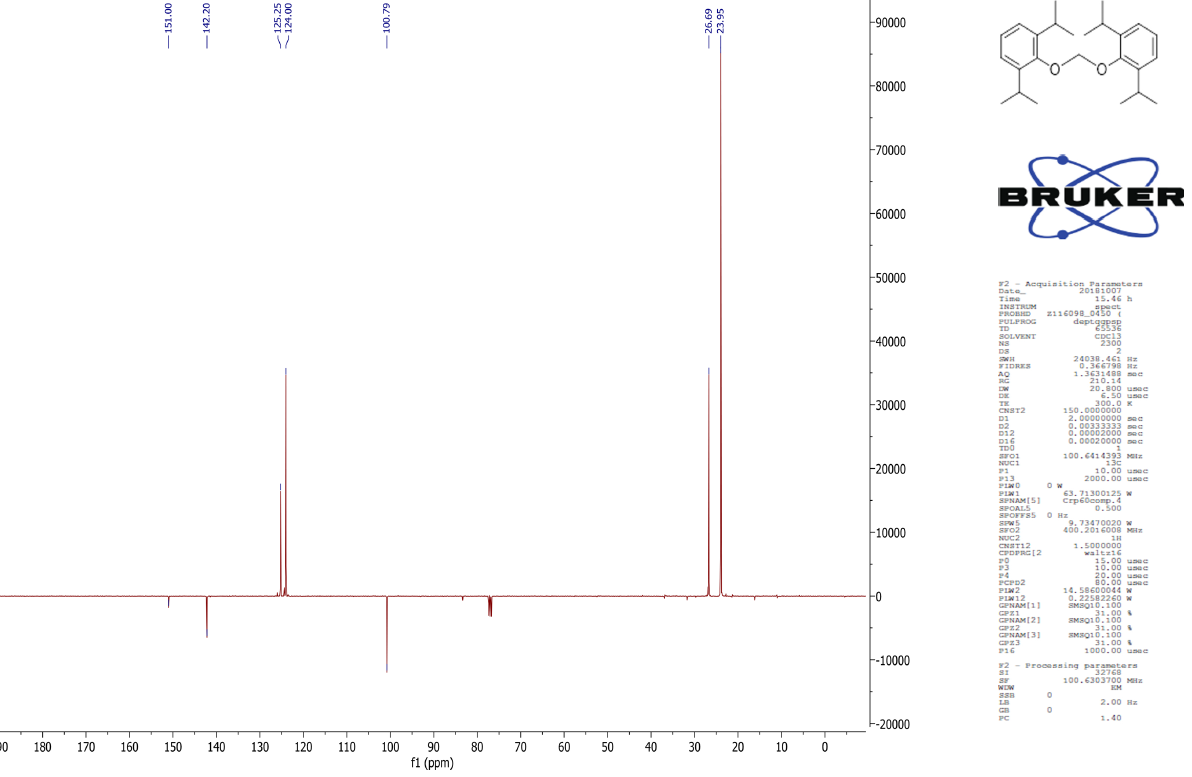


**Figure S52.** qDEPT spectra of **27**


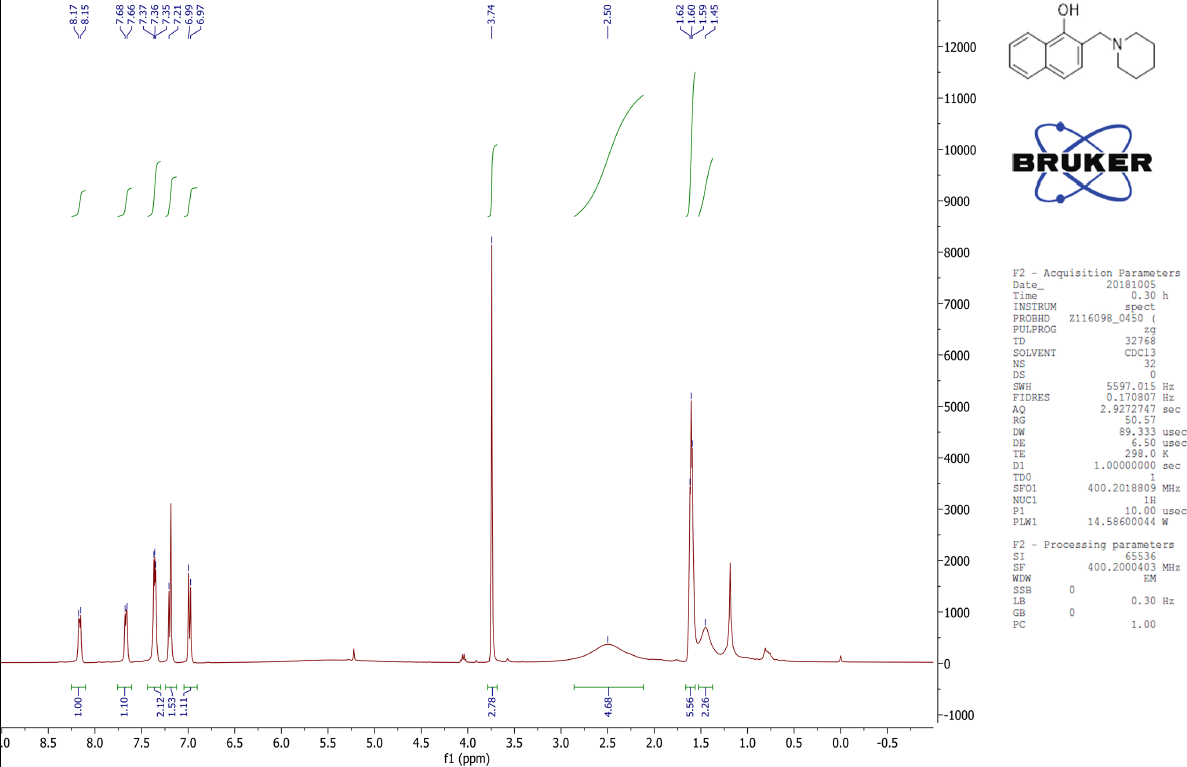


**Figure S53.** ^1^H spectra of **28**


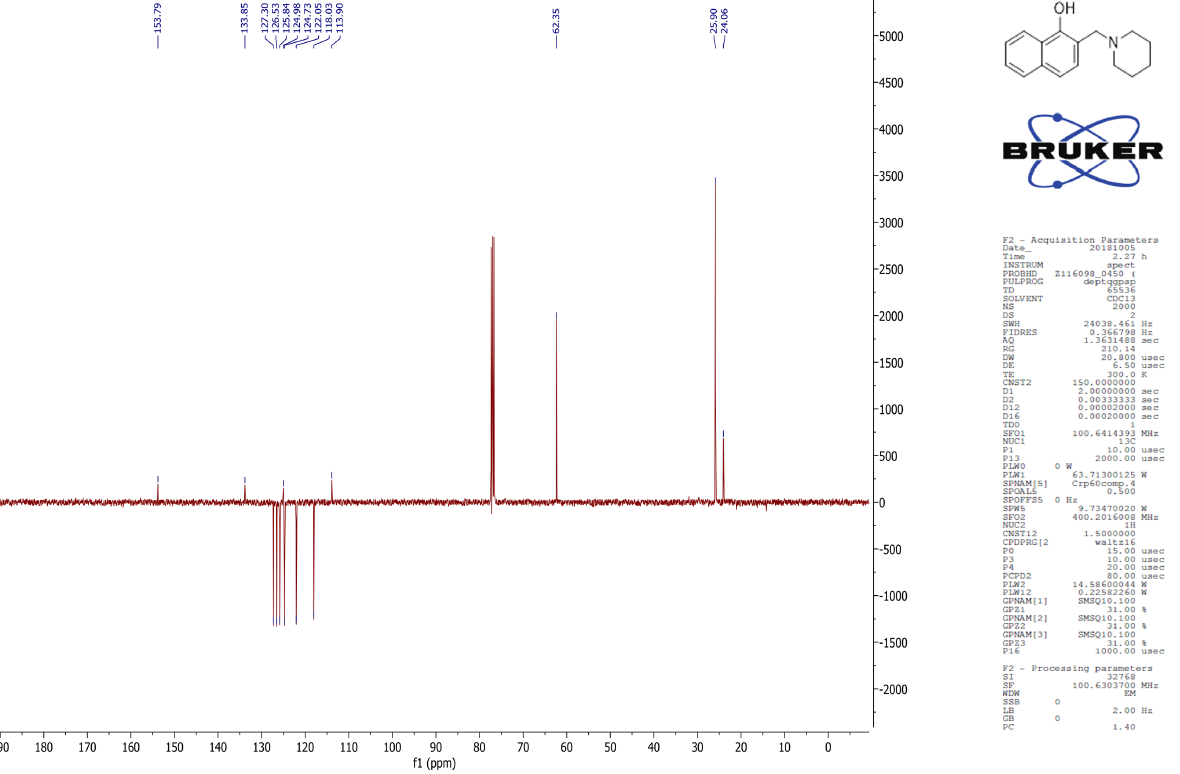


**Figure S54.** qDEPT spectra of **28**


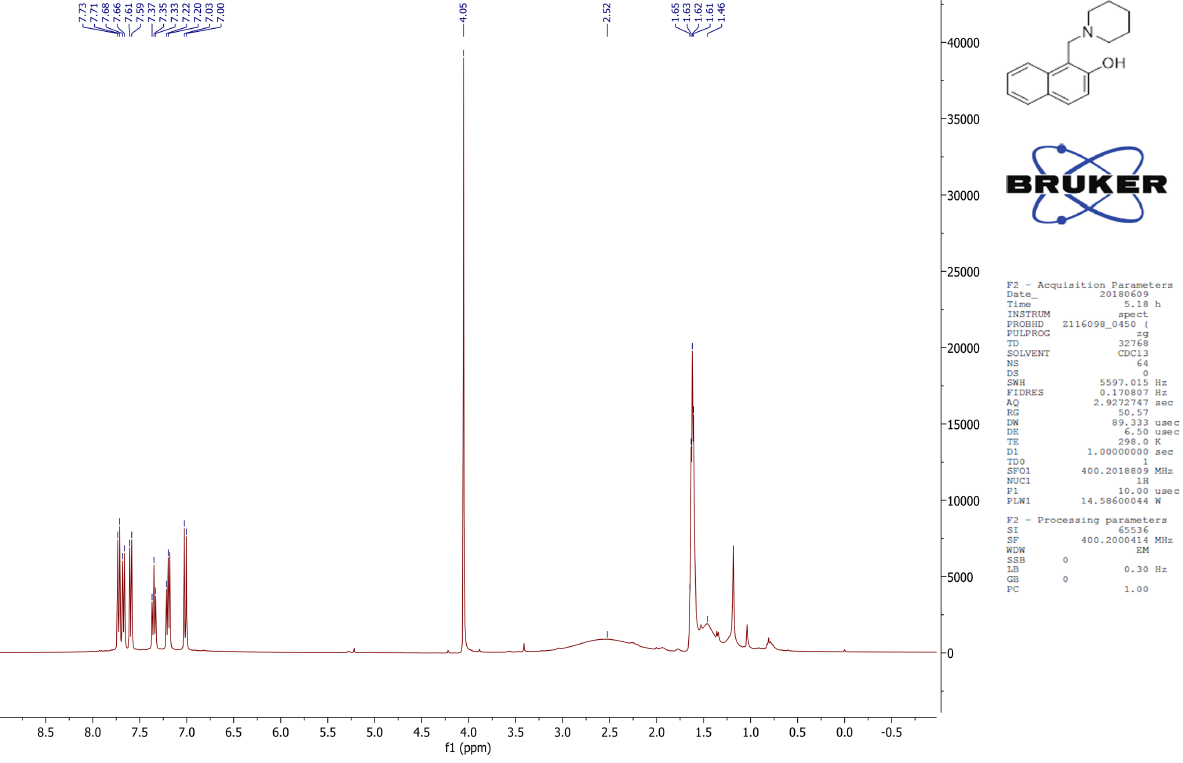


**Figure S55.** ^1^H spectra of **29**


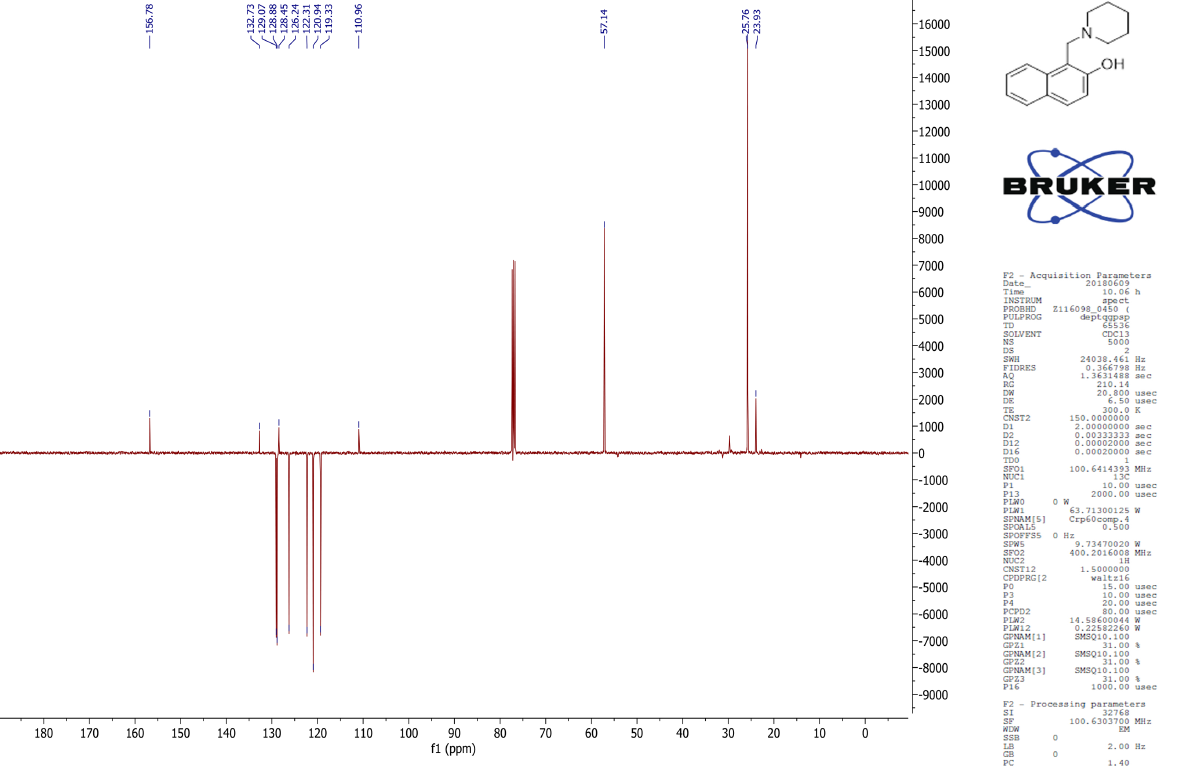


**Figure S56.** qDEPT spectra of **29**


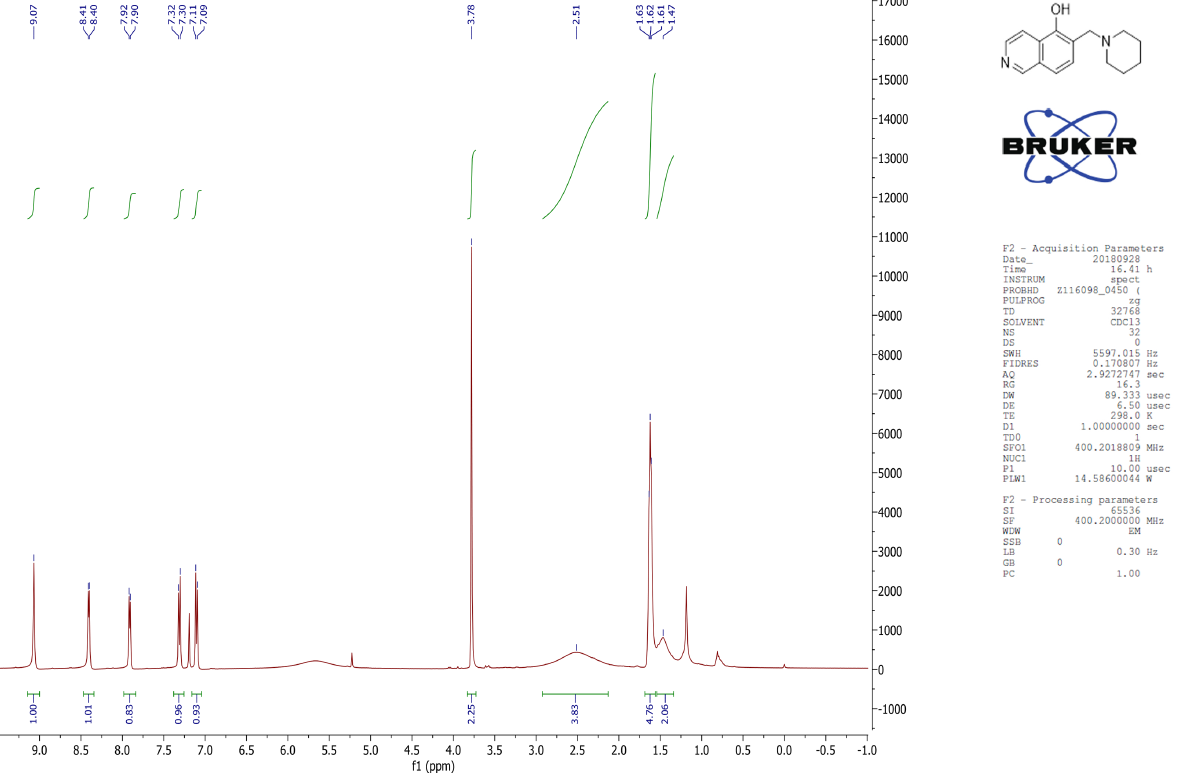


**Figure S57.** ^1^H spectra of **30**


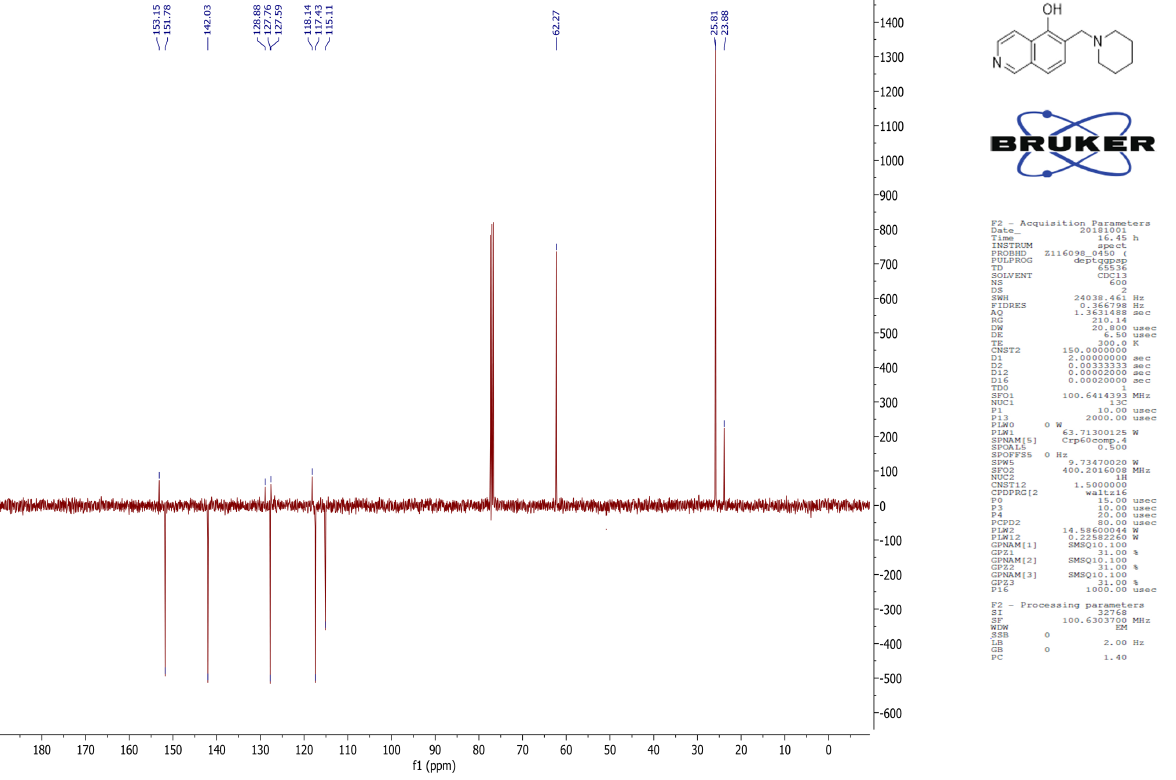


**Figure S58.** qDEPT spectra of **30**


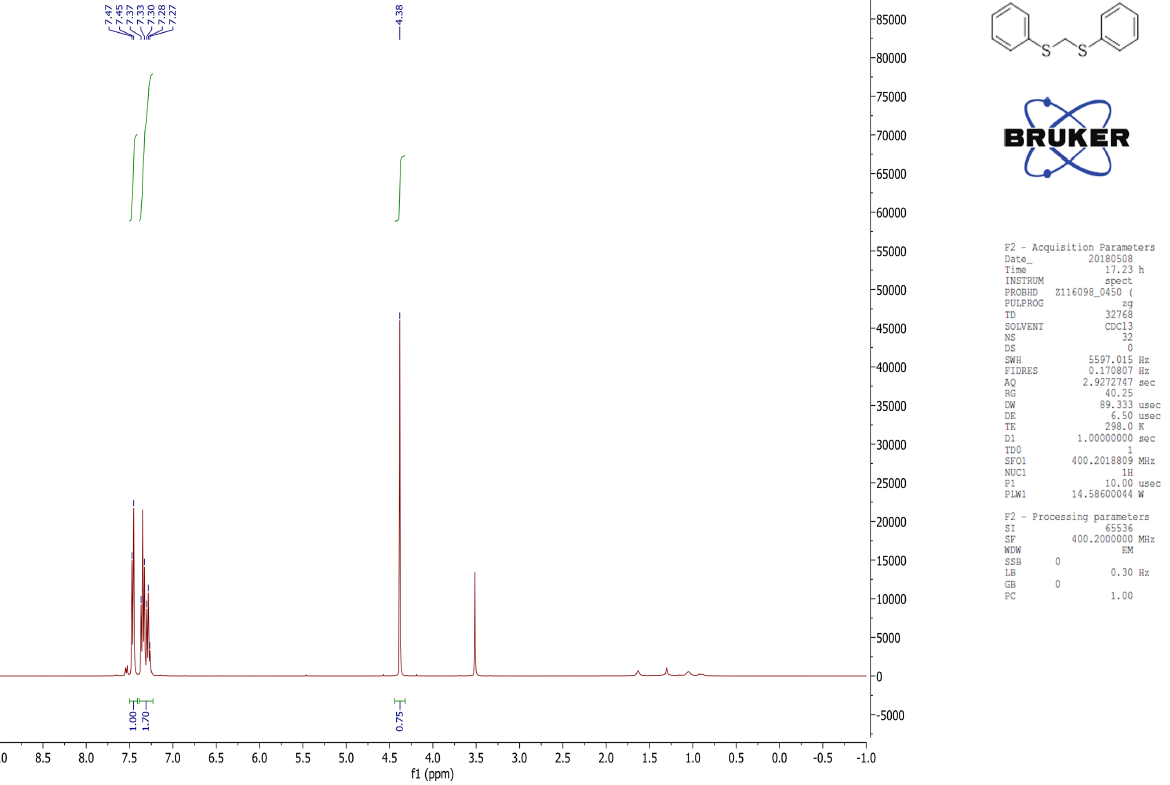


**Figure S59.** ^1^H spectra of **31**


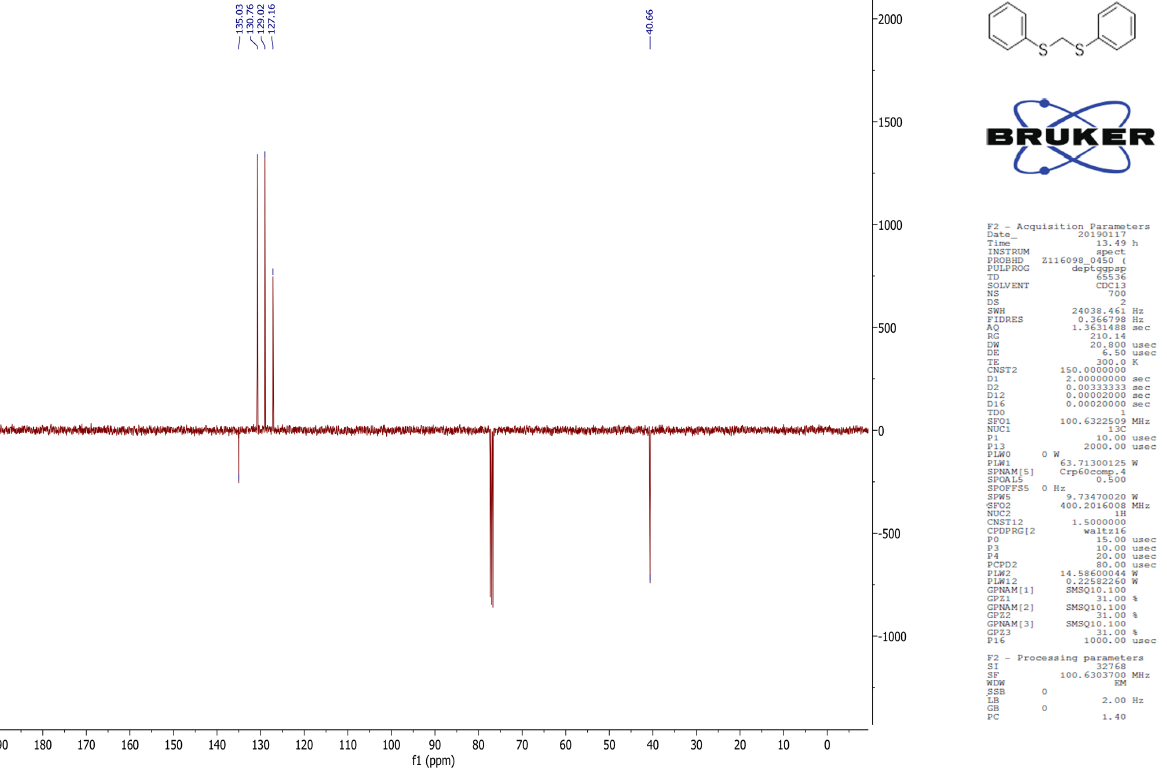


**Figure S60.** qDEPT spectra of **31**
